# Carbohydrate‐Directed Labeling Probes for UV‐Independent Chemoproteomic Profiling of Carbohydrate–Protein Interactions

**DOI:** 10.1002/anie.1584222

**Published:** 2026-09-18

**Authors:** Matthew C. Russolillo, Wariya Nirachonkul, Nora Ross, Rahul M. Hedau, Zachary Clark, Michaella J. Rekowski, Michael P. Washburn, Mark P. Farrell

**Affiliations:** ^1^ Department of Medicinal Chemistry University of Kansas Lawrence Kansas USA; ^2^ Mass Spectrometry and Proteomics Core University of Kansas Medical Center Kansas City Kansas USA; ^3^ Department of Cancer Biology University of Kansas Medical Center Kansas City Kansas USA

**Keywords:** carbohydrate‐binding proteins, carbohydrate–protein interactions, chemoproteomics, glycoscience, lectins, probes

## Abstract

Weak and transient carbohydrate–protein interactions hinder the detection of carbohydrate‐binding proteins (CBPs) in native settings. Here, we introduce carbohydrate‐directed labeling probes (CDLPs), modular ligand‐directed covalent probes composed of a glycan ligand, an acyl‐transfer electrophile, and a distal azide handle. Glycan binding positions the electrophile for proximity‐enhanced, glycan‐releasing acyl transfer, leaving an azide‐bearing acyl tag on the protein. Benchmark CDLPs capture carbohydrate–protein interactions with monovalent *K*_D_ values extending into the millimolar range. In Daudi B cells, a 6′‐sialyllactose CDLP preferentially captures CD22 (Siglec‐2), demonstrating glycan‐dependent labeling in live cells. Quantitative chemoproteomics using 6′‐sialyllactose‐ and lactose‐CDLPs yields complementary competition‐sensitive CDLP‐capture profiles (6′SL‐CDLP: 95; Lac‐CDLP: 113; 21 shared), including canonical CBPs, glycan‐processing proteins, and candidate proteins lacking prior glycan‐related annotations. CDLPs provide an accessible, UV‐independent route to durable covalent readouts for targeted validation and proteome‐scale candidate discovery.

## Graphical abstract

Modular carbohydrate‐directed labeling probes (CDLPs) convert weak carbohydrate–protein interactions into durable covalent tags in live cells without the need for multivalency or UV irradiation. Ligand binding triggers proximity‐enabled, glycan‐releasing acyl transfer to install a click handle for protein tagging and chemoproteomic profiling.

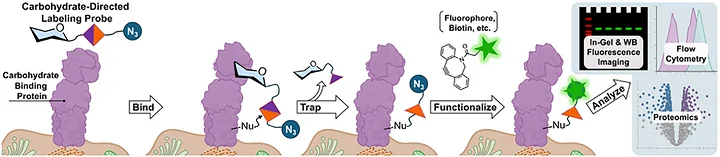

## 1 Introduction

Carbohydrate‐binding proteins orchestrate cell–cell communication, immune recognition, and pathogen adhesion [1, 2]. However, monovalent carbohydrate–protein interactions are often low‐affinity (K_D_ ≈ mid‐µM to mM) [3, 4], placing many physiologically relevant binding events beyond conventional affinity enrichment [5, 6, 7, 8, 9, 10]. Multivalent glycomaterials improve avidity and enable CBP profiling [11, 12, 13, 14], but their specialized synthesis can limit broad adoption. Photoaffinity carbohydrate probes enable covalent capture upon UV activation [3, 15]. While multivalent photoaffinity probes have successfully mapped carbohydrate–protein interactions [16, 17, 18, 19], monovalent variants often suffer from low crosslinking yields for weak interactions, and photoactivation is decoupled from binding occupancy, permitting quenching or nonspecific crosslinking [3, 20]. Although monovalent photoaffinity probes can be used for higher‐affinity interactions [21, 22], UV exposure and phototoxicity remain practical constraints in live‐cell studies [23, 24, 25]. Thus, simple monovalent probes that convert weak glycan binding into covalent capture without UV would be valuable.

Ligand‐directed acyl transfer offers an alternative approach [26]. Unlike photoaffinity probes, acyl transfer can couple labeling to productive ligand engagement, while still retaining intrinsic electrophile reactivity: probes bound in a favorable geometry can acylate proximal nucleophiles. Prior applications in glycobiology have focused mainly on high‐affinity CBPs, multivalent assemblies, or isolated proteins [27, 28, 29, 30, 31, 32, 33, 34, 35]. Extending this strategy to weak carbohydrate recognition in cells remains challenging because endogenous glycans compete for binding.

Here, we introduce carbohydrate‐directed labeling probes (CDLPs), which undergo binding‐promoted, glycan‐releasing acyl transfer to install an azide‐bearing acyl tag without UV activation (Figure 1). Productive glycan binding positions a dibromophenyl benzoate ester in proximity to protein nucleophiles, thereby releasing the carbohydrate ligand during acyl transfer while preserving a click‐compatible protein tag. Because labeling requires both binding and a suitably positioned nucleophile, CDLPs may favor targets with accessible nucleophiles near the glycan‐binding interface. We use 6′‐sialyllactose (6′SL) and lactose (Lac) as benchmark ligands, rather than to define exhaustive interactomes: 6′SL tests the weak, cis‐masked CD22 system, whereas lactose provides a non‐sialylated comparator. Thus, CDLPs complement photoaffinity and multivalent glycan tools as a UV‐independent strategy for targeted validation and proteome‐scale nomination of candidate CBPs.

**FIGURE 1:**
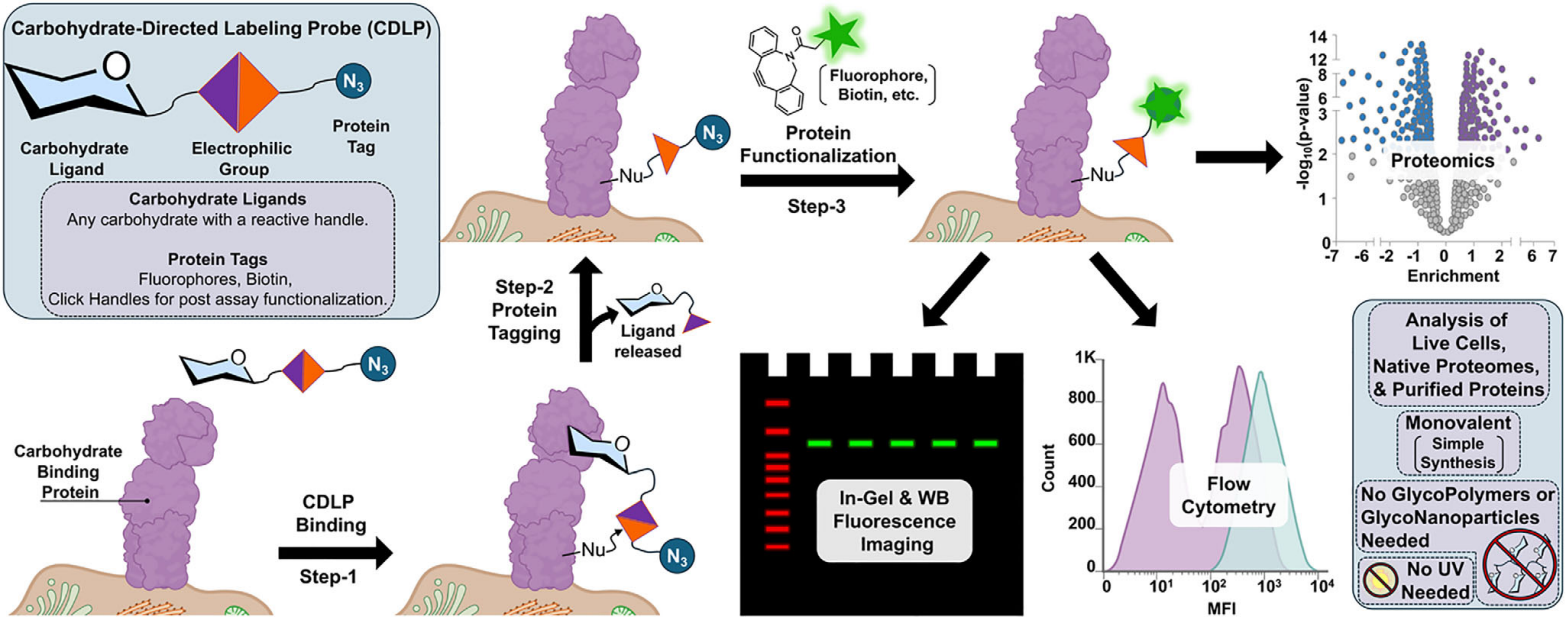
CDLP workflow for UV‐independent, ligand‐directed covalent capture of carbohydrate‐binding proteins. Productive glycan engagement positions the dibromophenyl benzoate acyl‐transfer electrophile near protein nucleophiles. Acyl transfer releases the carbohydrate ligand and leaves the protein covalently modified with an azide‐bearing acyl tag. The installed azide can then be functionalized by SPAAC with fluorophores for gel/flow readouts or with enrichment tags for chemoproteomic analysis. In the schematic, “ligand released” denotes the designed displacement of the carbohydrate ligand during acyl transfer; the protein retains the azide‐bearing acyl tag.

## 2 Results and Discussion

To translate this concept into practice, we developed an efficient route to a CDLP scaffold (**5**) based on a dibromophenyl benzoate aryl ester electrophile. This electrophilic motif was selected on the basis of prior reports describing a useful balance of reactivity and stability in biological settings [36]. Upon ligand binding, proximity can accelerate acyl transfer to nearby nucleophilic residues, converting transient recognition into a covalent adduct bearing the distal azide handle [37]. Structurally, the CDLPs follow a three‐component design in which a protein‐tagging element and a carbohydrate ligand are linked via a reactive ester. This architecture facilitates straightforward installation of both carbohydrate ligands and tagging handles, thereby providing a flexible platform for profiling carbohydrate–protein interactions. Azide‐bearing CDLPs enable copper‐free tagging via strain‐promoted azide‐alkyne cycloaddition (SPAAC) and are highlighted here [38]. The same scaffold also accommodates alternative bioorthogonal handles, a feature used below with an alkyne‐bearing 6′SL‐CDLP analog.

The bifunctional CDLP core scaffold **5** was assembled by coupling 3,5‐dibromo‐4‐hydroxybenzoic acid (**1**) with propargyl‐PEG3‐amine, followed by installation of an azido‐PEG4 tagging arm (Scheme 1A). Azido‐functionalized glycans were appended to the scaffold alkyne by copper(I)‐catalyzed azide‐alkyne cycloaddition (CuAAC) to afford glycan‐CDLPs (**6a**–**f**), leaving a distal azide handle for post‐labeling tagging. While many glycosyl azides are commercially available, for the monosaccharide probes, we used anomeric azidopropyl glycosides to distance the CuAAC‐generated triazole from the carbohydrate recognition elements, which are concentrated within a single sugar unit. In contrast, Lac‐ and sialyllactose‐CDLPs were prepared from reducing‐end glycosyl azides generated directly from the corresponding native oligosaccharides using Shoda's reagent [39, 40]. This shorter synthetic route was chosen to preserve the accessibility of the platform and was considered appropriate for the 6′SL/CD22 benchmark because the principal CD22‐recognition determinants reside in the terminal sialic acid and underlying galactose rather than the reducing‐end glucose [41]. To minimize nonspecific interactions with bulky reporters, the distal azide remained unmodified during labeling [42, 43, 44, 45, 46, 47]. Post‐labeling functionalization of the azide enables the flexible installation of fluorophores or enrichment tags, thereby increasing versatility.

**SCHEME 1:**
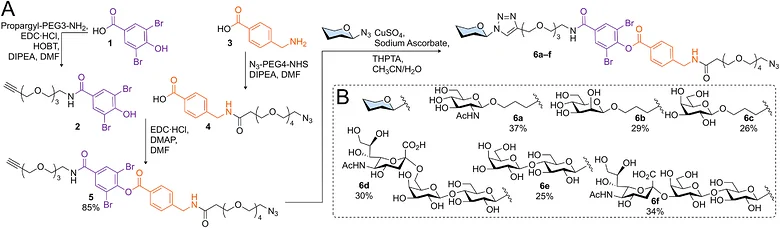
Modular synthesis of the CDLP core scaffold and glycan variants. (A) Synthesis of bifunctional CDLP scaffold **5** from 3,5‐dibromo‐4‐hydroxybenzoic acid (**1**), propargyl‐PEG3‐amine, and an azido‐PEG4 tagging arm. Isolated yields are shown for purified intermediates and products; intermediates **2** and **4** were used without purification, and scaffold **5** was isolated in 85% yield. (B) Glycan‐CDLPs **6a**–**f** prepared by CuAAC between scaffold **5** and the corresponding azide‐functionalized glycan. Isolated yields for each purified probe after HPLC are shown below the corresponding structure: **6a**, 37%; **6b**, 29%; **6c**, 26%; **6d**, 30%; **6e**, 25%; **6f**, 34%.

Before protein labeling, we evaluated the stability of representative CDLPs under assay‐relevant conditions. Lac‐CDLP **6e** showed little loss in PBS at 37°C over 24 h (Figure S1). Consistent with slow hydrolysis under these conditions, the 24 h PBS data suggest an apparent parent‐probe half‐life longer than the 24 h observation window. In the presence of N‐acetyllysine methyl ester, **6e** showed modest consumption, with ∼1% degradation after 4 h and ∼17% after 24 h, accompanied by LC–MS evidence of lysine‐adduct formation (Figure S2).

These data indicate that the aryl ester is chemically competent toward amine nucleophiles but is not rapidly consumed by bulk hydrolysis over the early labeling window. RNase B accelerated **6e** consumption by HPLC, with approximately 54% parent‐probe loss after 24 h, but produced only weak RNase B‐associated fluorescence after click tagging, indicating that probe consumption in proteinaceous samples does not necessarily translate into strong nonspecific bulk protein labeling (Figure S3). Under live‐cell‐medium conditions, 6′SL‐CDLP **6d** was consumed faster in serum‐free RPMI, with approximately 73% parent‐probe loss by 4 h, likely in part via soluble amino acid α‐amino groups, as evidenced by LC–MS‐detected amino acid adducts and hydrolysis products (Figure S4). Together, these data show that CDLP stability is assay‐context dependent: probes are comparatively stable in buffer, but medium components can promote turnover during extended live‐cell incubations. Because only representative probes were analyzed under each condition, these measurements should be interpreted as assay‐context benchmarking rather than a complete intrinsic reactivity profile for every CDLP. We therefore interpret long‐incubation chemoproteomic datasets as condition‐specific CDLP‐capture profiles.

CDLP labeling was tested with purified lectins (Figure 2A). Purified WGA (75 µM) was incubated with GlcNAc–CDLP 6a (0–300 µM), followed by addition of DBCO–MB488 to tag labeled protein via SPAAC. GlcNAc–CDLP 6a labeled WGA in a concentration‐dependent manner, as detected by DBCO–MB488 tagging, despite the weak monovalent WGA–GlcNAc interaction (*K*_D_ ≈ 1.8 mM, Figure 2B) [38, 48]. A DBCO–MB488‐only control was included to assess nonspecific fluorophore labeling. Free GlcNAc competition experiments were performed with WGA held at 75 µM and **6a** at 150 µM, while free GlcNAc was varied as indicated. Free GlcNAc inhibited **6a** labeling dose‐dependently, supporting carbohydrate‐recognition‐dependent capture rather than bulk probe reactivity alone (Figure 2C). The approach extended to additional lectin–glycan pairs: Man‐CDLP **6b** labeled ConA (*K*_D_ ≈ 0.47 mM), and ConA labeling was inhibited by free mannose (Figure 2D,E), while Gal‐CDLP **6c** labeled PNA (*K*_D_ ≈ 1 mM; Figure S5) [49, 50]. Additional purified‐protein benchmarking showed that labeling kinetics were probe‐ and target‐dependent (Figure S6). Matched lectin–probe pairs accumulated signal over time, while WGA/GlcNAc–CDLP **6a** and ConA/Man–CDLP **6b** showed preferential, competitor‐sensitive labeling compared with mismatched CDLPs and noncognate protein controls.

**FIGURE 2:**
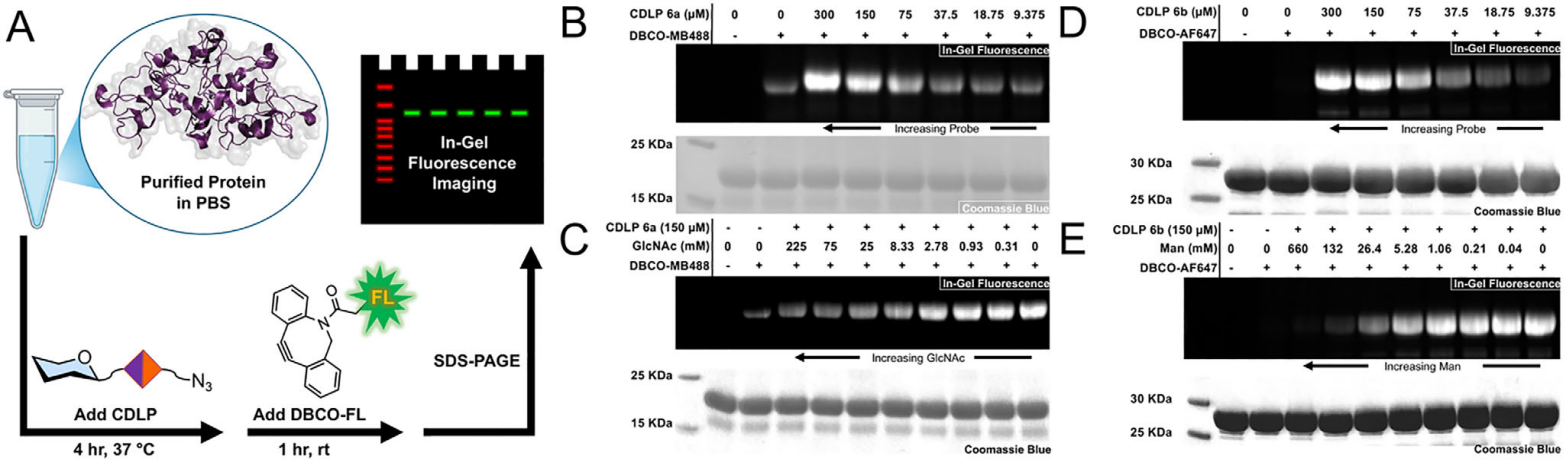
CDLPs covalently capture lectin–glycan interactions in vitro. (A) Purified lectins were incubated with matched glycan‐CDLP probes, functionalized after labeling with DBCO‐MB488 or DBCO‐AF647, and analyzed by SDS‐PAGE/in‐gel fluorescence followed by Coomassie staining. (B) Concentration‐dependent labeling of WGA by GlcNAc‐CDLP **6a** (WGA, 75 µM final lectin concentration). Fluorescence increases with probe concentration, while Coomassie staining shows comparable protein loading. DBCO‐only lanes are shown as negative controls. (C) Free GlcNAc competitively inhibits WGA labeling (WGA, 75 µM; **6a**, 150 µM), supporting carbohydrate‐recognition‐dependent capture. (D) Concentration‐dependent labeling of ConA by Man‐CDLP 6b (ConA, 75 µM final lectin concentration). (E) Free mannose competitively inhibits ConA labeling (ConA, 75 µM; **6b**, 150 µM).

CDLPs were next evaluated in live cells using CD22 (Siglec‐2) as a stringent benchmark. CD22 binds monomeric 6′SL weakly (*K*_D_ ≈ 300 µM) and engages in extensive cis‐interactions with sialoglycan‐decorated proteins, often masking its binding site (Figure S7) [41, 51]. Even high‐affinity synthetic ligands can show limited engagement with CD22 on B cells due to cis‐masking, making CD22 an exacting live‐cell benchmark for CBP‐labeling tools [52, 53]. Because CDLPs are designed to convert brief glycan encounters into covalent adducts, we reasoned that they could capture CD22 during transient windows of binding‐site accessibility without requiring multivalency or UV activation.

Daudi (CD22^+^) and Jurkat (CD22^−^) cells were treated with 6′SL–CDLP **6d** (50–500 µM), tagged proteins were fluorescently functionalized with DBCO–AF647 (200 µM), and lysates were analyzed by fluorescence gel imaging and immunoblotting. A dose‐dependent fluorescent band overlapped with CD22 in Daudi but not Jurkat cells, with β‐actin confirming comparable loading (Figure 3A,B). Flow cytometry similarly showed stronger dose‐dependent labeling in Daudi cells than in Jurkat cells (Figure 3C,D), and free 6′SL reduced **6d** labeling, supporting capture through engagement of the CD22 carbohydrate‐binding site (Figure 3E). Similar labeling and competition results with an alkyne‐bearing 6′SL–CDLP analog **S5** further demonstrated handle modularity (Figure S8).

**FIGURE 3:**
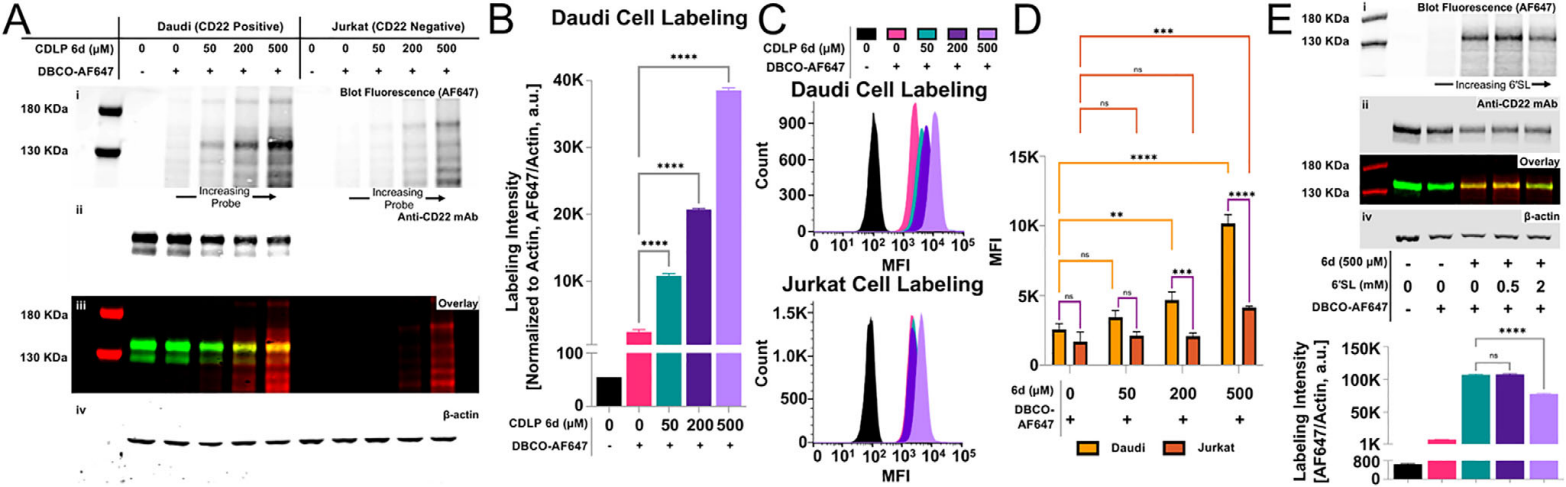
Live‐cell labeling and covalent CD22 capture by 6′SL‐CDLP **6d**. (A) Daudi (CD22^+^) and Jurkat (CD22^−^) cells were treated with 6′SL–CDLP **6d** at the indicated concentrations for 16 h, functionalized with DBCO–AF647, and analyzed by AF647 fluorescence imaging of CDLP‐labeled proteins, anti‐CD22 immunoblotting, fluorescence/CD22 overlay, and β‐actin loading control. A dose‐dependent CDLP‐derived fluorescent band overlaps with anti‐CD22 signal in Daudi cells, whereas Jurkat cells lack detectable CD22 and do not show a corresponding CD22‐associated CDLP band. (B) Quantification of CD22‐associated AF647 fluorescence normalized to β‐actin, plotted as individual biological replicates with mean ± SEM (*n* = 3). (C, D) Flow cytometry analysis showing stronger dose‐dependent 6′SL–CDLP labeling in Daudi cells than in Jurkat cells; MFI values are plotted as individual biological replicates, with mean ± SEM (*n* = 3). (E) Total‐lysate competition experiment showing that free 6′SL reduces 6′SL–CDLP‐derived labeling at the CD22‐associated band, supporting engagement of the CD22 carbohydrate‐binding site. Statistical analysis: panel B, ordinary one‐way ANOVA followed by Dunnett's multiple‐comparisons test; panels C,D, ordinary two‐way ANOVA followed by Dunnett's multiple‐comparisons test; panel E, ordinary one‐way ANOVA followed by Dunnett's multiple‐comparisons test. For all panels: *p < 0.05, **p < 0.01, ***p < 0.001, ****p < 0.0001; ns, not significant.

Ligand and scaffold controls further clarified the origin of the live‐cell signal. Lac‐CDLP **6e** and 3′SL‐CDLP **6f**, serving as glycan‐bearing ligand controls, failed to yield robust CD22 labeling in Daudi cells and showed minimal signal in Jurkat cells under the same conditions (Figure S9). We also evaluated unglycosylated scaffold **5** to assess scaffold‐derived background. Removing the glycan substantially increased calculated lipophilicity (Figure S10A). Consistent with this increased hydrophobicity, scaffold **5** produced broad labeling in both CD22^+^ Daudi and CD22^−^ Jurkat cells rather than a discrete CD22‐associated signal (Figure S10B,C). Because broad background bands were also observed to varying degrees in glycan‐CDLP‐treated lysates, we interpret **5** as a scaffold background control, whereas Lac‐CDLP **6e** and 3′SL–CDLP **6f** serve as more appropriate glycan‐bearing ligand controls for sialic‐acid‐dependent CD22 capture.

Time‐course studies showed dose‐ and time‐dependent accumulation of 6′SL–CDLP labeling in Daudi cells and low Jurkat signal; after probe washout, Daudi signal decayed over 24–72 h but remained detectable at early post‐wash time points (Figure S11), indicating that the covalent adduct can persist after probe removal. To assess CD22‐associated capture from the broader‐labeled proteome, Daudi cell proteins were functionalized with a desthiobiotin‐affinity tag, enriched on streptavidin beads, and analyzed by streptavidin‐AF647 detection of desthiobiotin‐tagged proteins together with anti‐CD22 immunoblotting. CD22 was preferentially recovered from 6′SL–CDLP **6d**‐treated cells; recovery decreased in the presence of the free 6′SL competitor and was weaker with Lac‐CDLP **6e** or 3′SL–CDLP **6f** (Figure S12). Thus, 6′SL–CDLP preferentially captured CD22, while total CDLP labeling also includes additional cellular proteins.

LC–MS/MS analysis of a dedicated CD22‐focused **6d** labeling experiment identified CD22 peptides bearing the CDLP‐derived acyl mass shift and localized modification to S304, K306, K421, and K667, providing residue‐level evidence for covalent CD22 tagging in cells (Appendix 1). Representative annotated MS/MS spectra are shown for K*VTTVIQNPMPIR and VQHSGAYWCQGTNSVGK*GR in Figure S13; in K*VTTVIQNPMPIR, the modified b1 ion at m/z 1254.48 supports modification of the N‐terminal Lys residue. These peptide‐level site‐mapping data were obtained from the CD22‐focused experiment, whereas the comparative chemoproteomics experiment was analyzed at the protein‐enrichment level.

In the present study, we interpret these residue‐level data primarily as evidence of target engagement and covalent acyl transfer. CD22 models in the Supporting Information are provided as illustrative visual references for the distribution of modified residues (Figure S14) [54]. As only a single target was examined at this level, and because CD22 conformational flexibility and intermolecular labeling within cis‐associated CD22 clusters cannot be excluded, we do not draw structural or conformational conclusions from these mappings [55]. The observed nominal ∼1126 Da mass shift is consistent with the expected SPAAC‐functionalized acyl tag (Figure S15), indicating glycan release during transfer. Thus, CDLPs record CD22 engagement through an azide‐bearing acyl tag rather than permanently appending the carbohydrate ligand, converting weak CD22–glycan recognition into durable live‐cell adducts.

The CD22 assignments described above, together with the broader dibromophenyl benzoate modification dataset, suggest that CDLP capture is nucleophile‐dependent and likely lysine‐favored, though not restricted to lysine. In the broader peptide‐level dataset, assigned adducts were predominantly localized to lysine residues, with smaller numbers assigned to serine, threonine, tyrosine, and rare histidine‐containing sites (Table S2). These observations should not be overinterpreted as a complete intrinsic residue‐reactivity hierarchy because peptide detectability and localization confidence also shape the observed distribution. A practical consequence is that CDLPs are not expected to provide chemically exhaustive coverage of all carbohydrate‐binding events. Instead, they provide a complementary strategy to photoaffinity labeling that may trade some breadth for UV‐independent, ligand‐directed covalent capture.

We next used competition‐based quantitative chemoproteomics to test whether CDLP glycan identity produces distinct ligand‐sensitive capture profiles in live Daudi cells [56, 57, 58, 59]. The 6′SL–CDLP **6d** served as a sialylated benchmark for CD22/Siglec capture, whereas Lac–CDLP **6e** served as both a non‐sialylated comparator for sialic acid‐dependent capture and a complementary benchmark with its own free‐lactose competition condition. These experiments were not designed to define exhaustive interactomes of free 6′SL or lactose, but to evaluate whether a common monovalent acyl‐transfer scaffold can nominate different classes of ligand‐sensitive targets under live‐cell labeling conditions.

Daudi cells were treated with either 6′SL–CDLP **6d** or Lac–CDLP **6e** (500 µM, 16 h) in the absence or presence of free glycan competitor (30 mM), followed by SPAAC‐mediated desthiobiotin tagging, streptavidin enrichment, and label‐free LC–MS/MS quantification (*n* = 3 biological replicates; Figure S16A) [60]. As CDLP labeling rates varied across probe–target pairs in both purified‐lectin benchmarking and live‐cell CD22 time‐course experiments, the 16 h incubation served as a discovery‐oriented chemoproteomics enrichment condition rather than a defined, universal labeling endpoint (Figures S6 and S11). We interpret these datasets as condition‐specific, competition‐sensitive CDLP‐capture profiles, not exhaustive interactomes of free 6′SL or lactose. Full protein‐level quantification is provided in Appendix 2. Competition‐sensitive enrichment was calculated as log_2_[(probe)/(probe + competitor)], yielding ligand‐sensitive profiles for candidate prioritization. DepMap transcript abundance showed limited correlation with probe enrichment (Figure S16B,C) [61, 62]. This limited correlation suggests that CDLP enrichment is not simply explained by transcript‐inferred protein expression, supporting competition‐sensitive enrichment rather than abundance alone for prioritization.

The 6′SL–CDLP **6d** enriched CD22, SIGLEC‐10, CD33/SIGLEC‐3, and immune/adhesion‐associated receptors (Figure 4A) [63, 64], whereas Lac–CDLP 6e enriched glycoprotein quality‐control and glycan‐processing proteins, including CANX, UGGT1, and EDEM3 (Figure 4B). The resulting profiles were distinct but partially overlapping (Figure 4C–F). The subcellular localization profiles in Figure 4E should not be attributed solely to ligand identity: 6′SL–CDLP **6d** and Lac–CDLP **6e** differ in glycan structure, size, charge, and likely lipophilicity, which may influence membrane partitioning, uptake, trafficking, and intracellular accessibility during the 16 h labeling window [65]. Thus, Figure 4E reflects probe‐dependent localization shaped by ligand recognition, physicochemical properties, and incubation time. Both profiles included canonical CBPs and glycan‐processing enzymes, but 84 6′SL–CDLP‐ and 91 Lac–CDLP‐enriched proteins lacked prior carbohydrate‐related annotation (Tables S3 and S4) [66]. We treat these as competition‐sensitive CDLP‐captured candidates, some of which may represent glycan‐engaged targets, as many target affinities are unknown and long incubations may allow cumulative background labeling or incomplete competition with very weak interactions.

**FIGURE 4:**
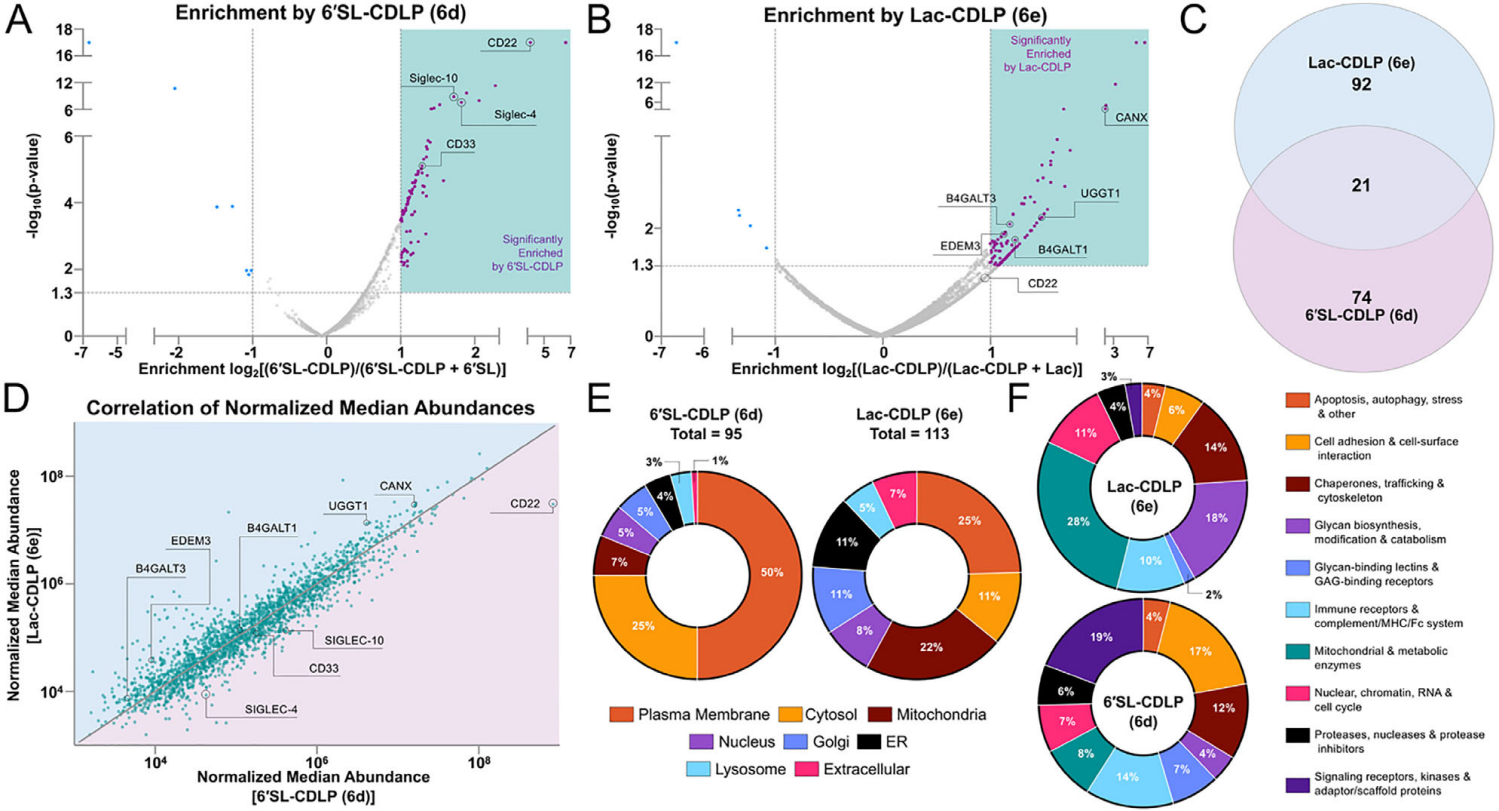
Comparative chemoproteomic profiling of proteins captured by 6′SL‐ and Lac‐CDLPs. (A, B) Volcano plots of competition‐sensitive enrichment for 6′SL–CDLP **6d** (A) and Lac‐CDLP **6e** (B) in Daudi cells treated with probe alone or probe plus the corresponding free glycan competitor (500 µM probe, 30 mM competitor, 16 h; *n* = 3 biological replicates). Proteins were prioritized as competition‐sensitive candidate proteins using log_2_[(probe)/(probe + competitor)] ≥ 1 and nominal *p* ≤ 0.05; protein‐level p‐values were not adjusted for multiple testing. (C) Overlap of competition‐sensitive candidate proteins recovered by the two probes. Numbers indicate the counts of unique and shared competition‐sensitive candidates. (D) Correlation of normalized median protein abundances measured with 6′SL–CDLP **6d** and Lac–CDLP **6e**; the diagonal indicates equal abundance. (E) Subcellular localization annotations for competition‐sensitive candidate proteins captured by each probe. These distributions reflect proteins recovered under the applied chemoproteomic conditions and should not be attributed solely to ligand identity; they may also be influenced by ligand recognition, probe size, charge, lipophilicity, uptake/trafficking, intracellular accessibility, and the 16 h labeling window. (F) Functional classification of proteins captured by 6′SL–CDLP **6d** and Lac–CDLP **6e**. These profiles nominate competition‐sensitive CDLP‐captured candidate proteins and do not by themselves prove direct glycan binding for every enriched protein.

STRING/stringApp analysis in Cytoscape using the CDLP‐detected proteome as background showed significant protein–protein interaction enrichment and probe‐selective annotation signatures: 6′SL–CDLP profiles were enriched for surface, adhesion, and immune terms, whereas Lac–CDLP profiles were enriched for glycan‐processing and metabolic terms (Figure S18) [67, 68, 69]. These analyses prioritize structured candidate sets rather than proving direct glycan binding by every enriched protein.

Selected candidates were corroborated by immunoblotting using the same desthiobiotin/streptavidin enrichment workflow. CD22 and selected 6′SL–CDLP candidates, including TFRC and GOT2, as well as Lac–CDLP candidates, including CANX/calnexin and FH/fumarate hydratase, were recovered from enriched fractions (Figure S19), providing independent immunoblot‐based validation of representative CDLP‐captured proteins from both datasets.

## 3 Conclusion

In summary, CDLPs establish a monovalent, UV‐independent platform for converting weak glycan recognition into durable covalent readouts across purified proteins, live cells, and native proteomes. Here, 6′SL and lactose serve as benchmark ligands to test whether glycan identity within a common acyl‐transfer scaffold can direct distinct competition‐sensitive capture profiles, not to define exhaustive interactomes of these glycans. Because capture depends on productive glycan binding, the availability of a proximal nucleophile, the probe's physicochemical properties, and non‐zero intrinsic aryl‐ester reactivity, CDLP datasets should be interpreted as ligand‐sensitive enrichment profiles that nominate candidates for follow‐up validation. Future work should prioritize physicochemically matched probe series, shorter labeling windows, broader residue‐level mapping, and orthogonal validation of candidate targets.

## Author Contributions

**Matthew C. Russolillo**: conceptualization, investigation, writing – original draft, methodology, validation, writing – review and editing, formal analysis, data curation. **Wariya Nirachonkul**: investigation, writing – original draft, methodology, visualization, formal analysis, data curation, writing – review and editing. **Nora Ross**: conceptualization, investigation, writing – review and editing, formal analysis. **Rahul M. Hedau**: conceptualization, investigation, data curation, formal analysis. **Zachary Clark**: investigation, data curation, formal analysis, methodology. **Michaella J. Rekowski**: investigation, writing – review and editing, methodology, formal analysis, data curation, visualization. **Michael P. Washburn**: investigation, funding acquisition, writing – review and editing, data curation. **Mark P. Farrell**: conceptualization, investigation, funding acquisition, writing – original draft, writing – review and editing, visualization, formal analysis, project administration, resources, supervision, data curation.

## Conflicts of Interest

The authors declare no conflicts of interest.

## Supporting information




**Supporting File**: The authors have cited additional references within the Supporting Information [70, 71, 72, 73, 74, 75, 76, 77]. **Supporting File 1**: anie74765‐sup‐0001‐SuppMat.pdf.

**Supporting File 2**: Appendix 1: anie74765‐sup‐0002‐SuppMat.xlsx.

**Supporting File 3**: Appendix 2: anie74765‐sup‐0003‐SuppMat.xlsx.

## Data Availability

The data that support the findings of this study are openly available in MassIVE at https://massive.ucsd.edu/, reference number MSV000098943.
