# Supplementary material for: Carbohydrate‐Directed Labeling Probes for UV‐Independent Chemoproteomic Profiling of Carbohydrate–Protein Interactions
Source: Angew Chem Int Ed Engl. 2026 Sep 18;65(44):e1584222. doi: 10.1002/anie.1584222 (PMC13703801; doi:10.1002/anie.1584222)
Supplement: Supplementary file 1 [file ANIE-65-e1584222-s001.pdf]

Carbohydrate-Directed Labeling Probes for UV-Independent Chemoproteomic Profiling of Carbohydrate–Protein Interactions

Matthew C. Russolillo‡,[a] Wariya Nirachonkul‡,[a] Nora Ross,[a] Rahul M. Hedau,[a] Zachary Clark,[b, c] Michaella J. Rekowski,[b, c] Michael P. Washburn,[b, c] Mark P. Farrell*[a]

[a] Dr. M. C. Russolillo, Dr. W. Nirachonkul, Dr. N. Ross, Mr. R. M. Hedau, Prof. Dr. M. P. Farrell
University of Kansas
2034 Becker Drive
Lawrence, KS 66047 (USA)
E-mail: markfarrell@ku.edu

[b] Mr. Z. Clark, Prof. Dr. M. J. Rekowski, Prof. Dr. M. P. Washburn
Mass Spectrometry and Proteomics Core
University of Kansas Medical Center
3901 Rainbow Boulevard
Kansas City, KS 66160 (USA)

[c] Prof. Dr. M. J. Rekowski, Prof. Dr. M. P. Washburn
Department of Cancer Biology
University of Kansas Medical Center
3901 Rainbow Boulevard
Kansas City, KS 66160 (USA)

‡ Both authors contributed equally to this work.

Table of Contents

ABBREVIATIONS **4**

SUPPLEMENTARY FIGURES **6**

Supplementary Figure S1 .... *6*
Supplementary Figure S2 .... *7*
Supplementary Figure S3 .... *8*
Supplementary Figure S4 .... *9*
Supplementary Figure S5 .... *10*
Supplementary Figure S6 .... *11*
Supplementary Figure S7 .... *12*
Supplementary Figure S8 .... *13*
Supplementary Figure S9 .... *14*
Supplementary Figure S10 .... *15*
Supplementary Figure S11 .... *16*
Supplementary Figure S12 .... *16*
Supplementary Figure S13 .... *17*
Supplementary Figure S14 .... *18*
Supplementary Figure S15 .... *19*
Supplementary Figure S16 .... *20*
Supplementary Figure S17 .... *21*
Supplementary Figure S18 .... *22*
Supplementary Figure S19 .... *23*
Full Gel and Blot Images: Figure S20–23 .... *24*

MATERIALS AND INSTRUMENTATION **28**

Chemistry Reagents and Solvents *28*
Cell Culture and Reagents *28*

EXPERIMENTAL PROCEDURES **29**

SYNTHETIC PROCEDURES 29
General Procedure for Shoda Reagent Activation *30*
Activated Carbon Column Purification *30*
Synthesis of Monomeric Carbohydrate Ligands *30*
General Click Procedure *30*
Synthesis of 6'-Sialyllactose Alkyne CDLP (S5) *33*

LECTIN LABELING **36**

Lectin Labeling Competition Experiments *36*
Lectin–CDLP Labeling Time-Course Assays *36*
Lectin Selectivity and Competition Assays *36*
General RP-HPLC Method for CDLP Stability and Degradation Assays *37*
Stability of Lac-CDLP 6e in PBS and in the Presence of N-Acetyllysine Methyl Ester *37*
Degradation of Lac-CDLP 6e in the Presence of RNase B *37*
Degradation of 6'SL-CDLP 6d in Serum-Free RPMI Medium *37*
LC-MS Assignment of CDLP Degradation Products *38*

CELL ASSAYS AND SAMPLE ANALYSIS **39**

IMMUNOBLOTTING ANALYSIS OF CDLP BINDING PROTEINS 39
FLOW CYTOMETRY ANALYSIS OF 6'SL-CDLP BINDING PROTEINS 40
LIVE-CELL 6'SL-CDLP TIME-COURSE AND WASHOUT-PERSISTENCE ASSAYS 40

PROTEOMIC PROFILING **41**

Cell Treatment and Lysate Preparation 41
Streptavidin-Desthiobiotin Enrichment of CDLP-Binding Proteins 41
Sample Preparation for LC-MS/MS Analysis 41
LC-MS/MS Detection and Data Analysis 42
Data Availability Statement *42*
Assessment of Prior Carbohydrate-Related Annotation *42*
STRING Network Analysis of CDLP-Captured Protein Profiles *43*

SUPPLEMENTARY TABLES **44**

Table S1. LC-MS assignments of 6'SL-CDLP 6d degradation products formed in media *44*
Table S2. Residue distribution of dibromophenyl benzoate-modified peptide entries *45*
Table S3. Competition-Sensitive Protein Candidates from 6'SL-CDLP 6d Profiling *46*
Table S4. Competition-Sensitive Protein Candidates from Lac-CDLP 6e Profiling *49*

NMR SPECTRA **52**

Abbreviations

°C: Degrees Celsius
3′SL: α2,3-Sialyllactose
6′SL: α2,6-Sialyllactose
7-AAD: 7-aminoactinomycin D
a.u.: arbitrary unit
AF647: Alexa Fluor 647 Dye
Aq.: Aqueous
BCA: Bicinchoninic Acid
BSA: Bovine Serum Albumin
$CaCl_2$: Calcium Chloride
calcd: Calculated
CBP: Carbohydrate-Binding Protein
CD22: Siglec-2
CD_3CN: Deuterated Acetonitrile
$CDCl_3$: Deuterated Chloroform
CD_3OD: Methanol-d4
CDLP: Carbohydrate-Directed Labeling Probe
CH_2Cl_2: Dichloromethane
CH_3CN: Acetonitrile
CH_3OH: Methanol
cm: Centimeter
CO_2: Carbon Dioxide
ConA: Concanavalin A
CuAAC: Copper-Catalyzed Azide-Alkyne Cycloaddition
$CuSO_4$: Copper (II) Sulfate
D_2O: Deuterium Oxide
DBCO-AF647: Dibenzocyclooctyne-Alexa Fluor 647 Dye
DBCO-MB488: Dibenzocyclooctyne-MB488
DIPEA: N,N-Diisopropylethylamine
DMAP: 4-Dimethylaminopyridine
DMC: 2-Chloro-1,3-dimethylimidazolinium Chloride
DMF: Dimethylformamide
DMSO: Dimethyl Sulfoxide
DTT: Dithiothreitol
EDC·HCl: 1-Ethyl-3-(3-Dimethylaminopropyl) Carbodiimide Hydrochloride
equiv.: Equivalent
ER: Endoplasmic Reticulum
ESI: Electrospray Ionization
FA: Formic Acid
FAIMS: Field Asymmetric Ion Mobility Spectrometry
FBS: Fetal Bovine Serum
g: Gram
Gal: Galactose
GlcNAc: N-Acetylglucosamine
h: Hour
H_2O: Water
HBSS: Hanks' Balanced Salt Solution
HCD: Higher Energy Collision Dissociation
HCl: Hydrochloric Acid
HEPES Buffer: 4-(2-hydroxyethyl)-1-piperazineethanesulfonic acid
HOBt: 1-Hydroxybenzotriazole
HRMS: High-Resolution Mass Spectrometry
IAA: Iodoacetamide
K_D: Dissociation Constant
kDa: Kilodalton
Lac: Lactose
LC-MS/MS: Liquid Chromatography-Tandem Mass Spectrometry
M: Molar
m/z: Mass-to-Charge Ratio
mAb: Monoclonal Antibody
Man: Mannose
MFI: Median Fluorescence Intensity
mg: Milligram
MHz: Megahertz
min: Minute
mL: Milliliter
mm: Millimeter
mM: Millimolar
mmol: Millimole
MW: Molecular Weight
MWCO: Molecular Weight Cutoff
N_3: Azide
Na_2CO_3: Sodium Carbonate
Na_2SO_4: Sodium Sulfate
NaCl: Sodium Chloride
NCE: Normalized Collisional Energy

ng: Nanogram
NHS: N-Hydroxysuccinimide
NMR: Nuclear Magnetic Resonance
Nu: Nucleophile
PBS: Phosphate Buffered Saline
PEG: Polyethylene Glycol
PNA: Peanut Agglutinin
QDa: Quadrupole Dalton
RIPA Buffer: Radioimmunoprecipitation Assay Buffer
RNase B: Ribonuclease B
RP: Reverse Phase
RPM: Revolutions Per Minute
RPMI: Roswell Park Memorial Institute 1640 Medium
s: Seconds
SD: Standard Deviation
SDS-PAGE: Sodium Dodecyl Sulfate–Polyacrylamide Gel Electrophoresis
SEM: Standard Error of the Mean
SPAAC: Strain-Promoted Azide-Alkyne Cycloaddition
TBST: Tris-Buffered Saline with Tween-20
TCEP: Tris(2-carboxyethyl)phosphine Hydrochloride
TEAB: Triethylammonium Bicarbonate Buffer
TFA: Trifluoroacetic Acid
THF: Tetrahydrofuran
THPTA: Tris(3-hydroxypropyltriazolylmethyl)amine
TLC: Thin-Layer Chromatography
TOF: Time-Of-Flight
U: Unit
UPLC: Ultra Performance Liquid Chromatography
UV: Ultraviolet
WB: Western Blot
WGA: Wheat Germ Agglutinin
α: Alpha
β: Beta
µg: Microgram
µL: Microliter
µm: Micrometer
µM: Micromolar

Supplementary Figures

Supplementary Figure S1

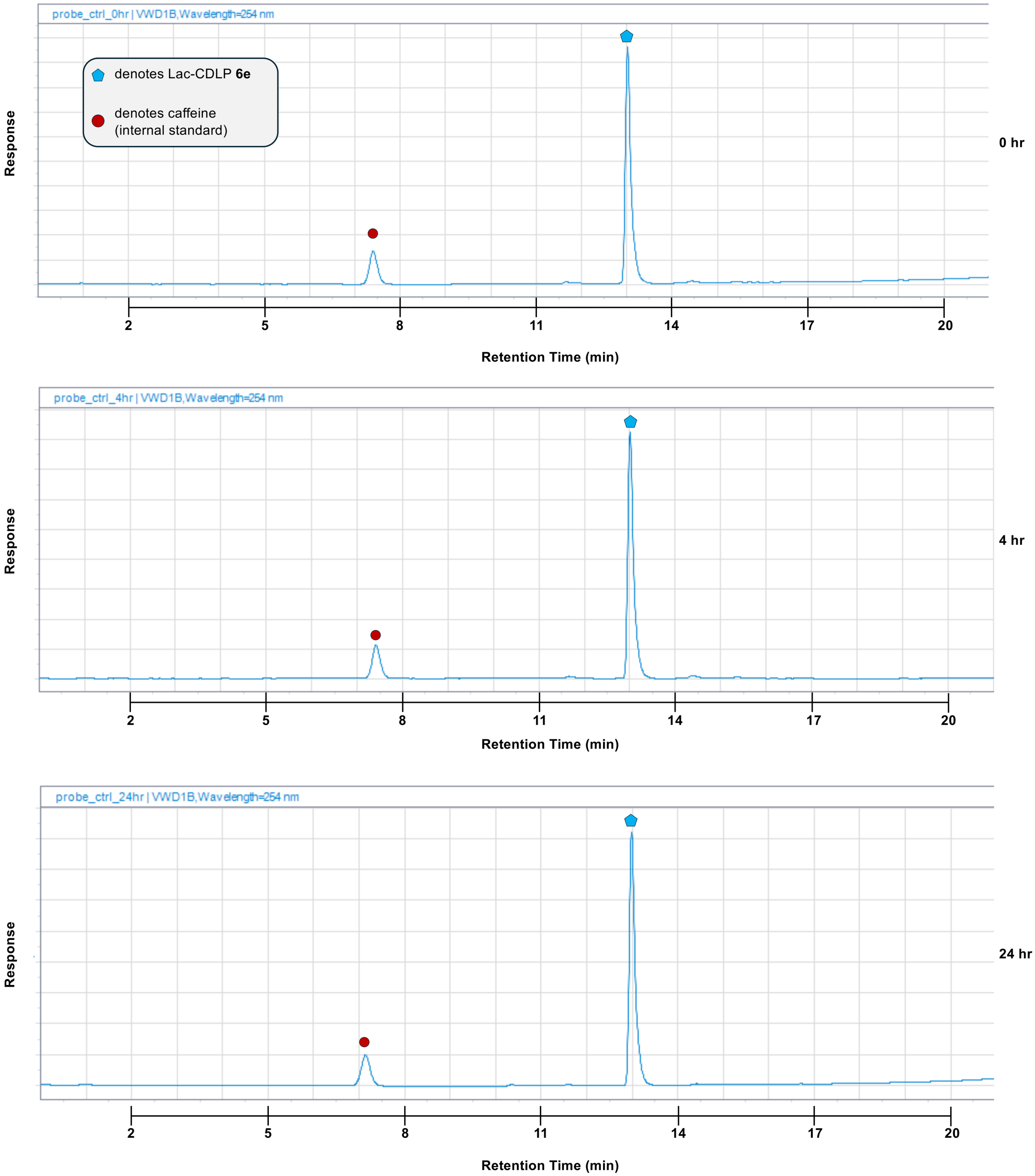

Figure S1. Stability of Lac-CDLP **6e** in PBS. Representative HPLC chromatograms monitoring Lac-CDLP **6e** in PBS at 0, 4, and 24 h. The Lac-CDLP **6e** peak is indicated by the blue pentagon, and the caffeine internal standard is indicated by the red circle. Minimal change in the parent probe peak relative to the internal standard over 24 h indicates that Lac-CDLP **6e** is stable under PBS control conditions.

Supplementary Figure S2

A.

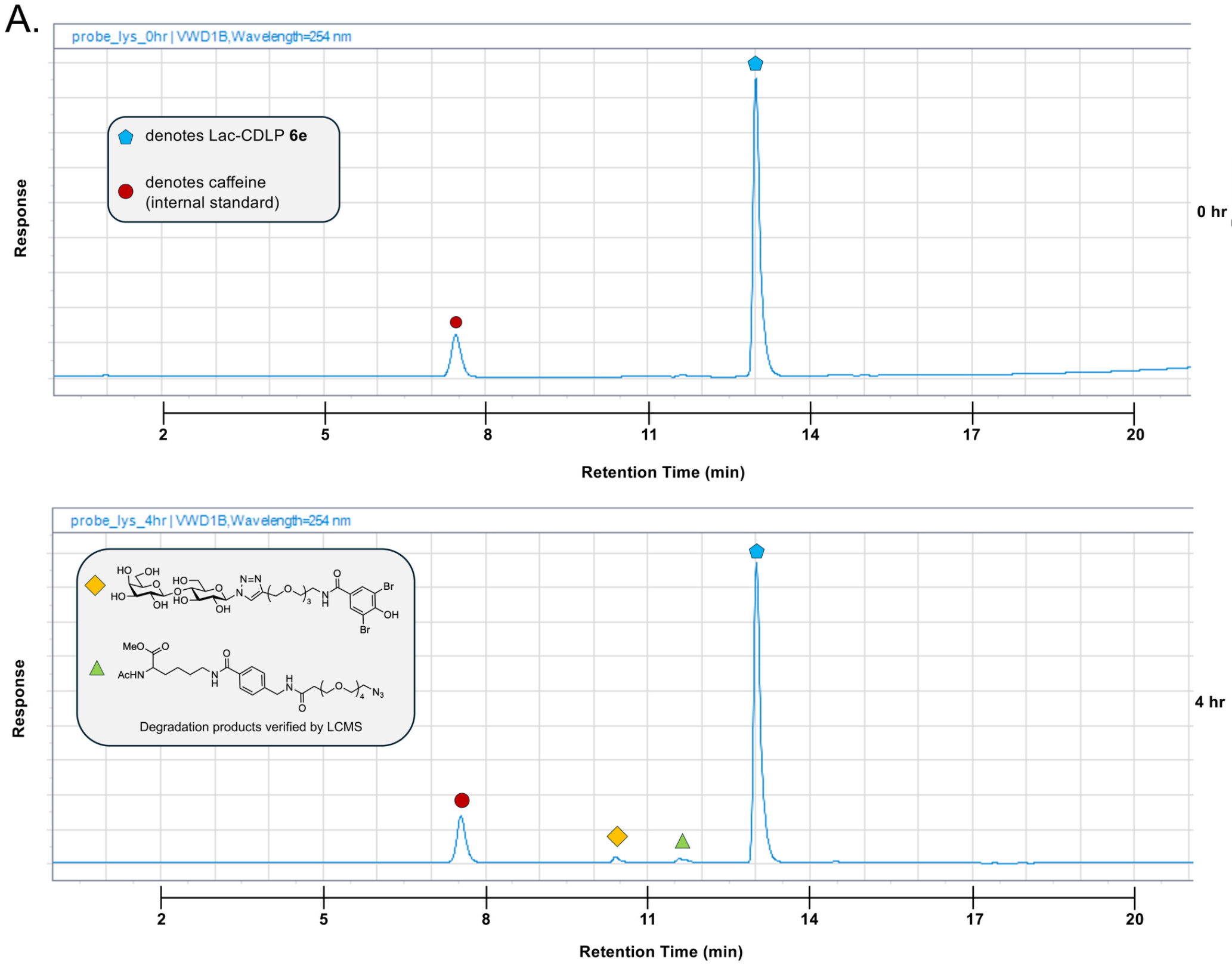

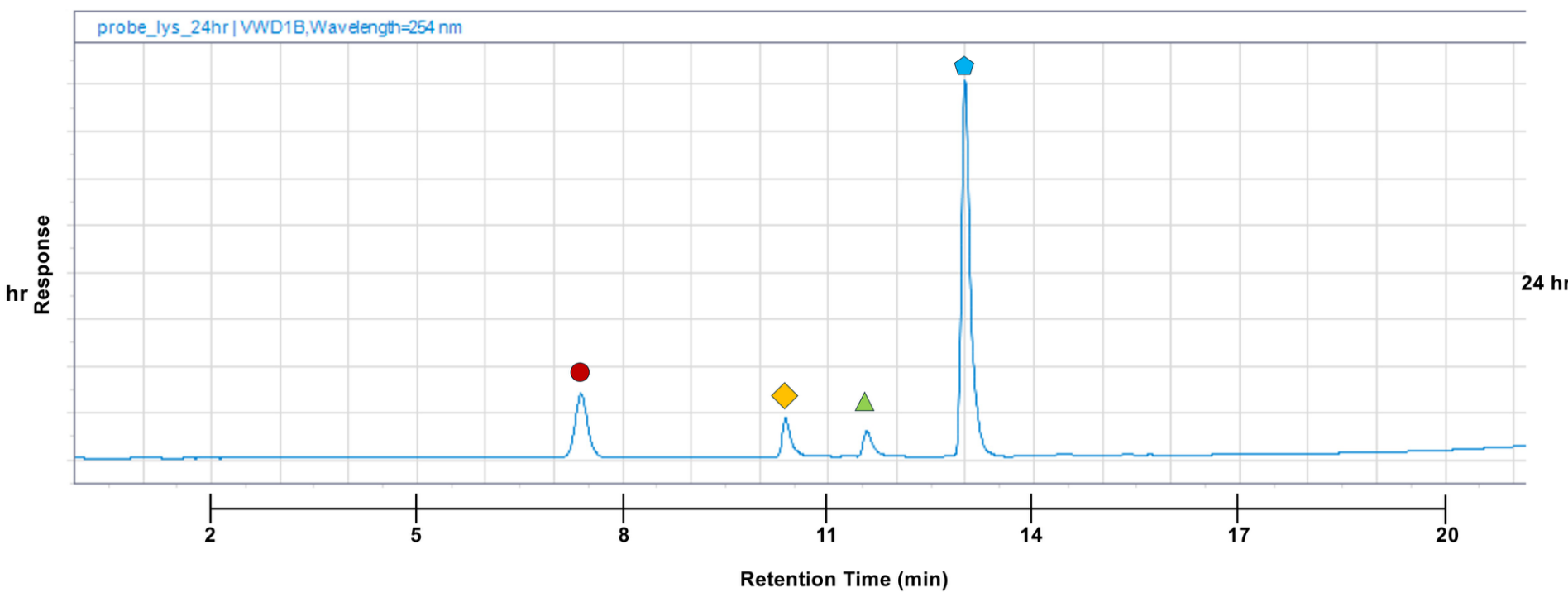

B.

0 hr

#	Name	Signal description	RT (min)	Area (mAU·s)	Area%	Height (mAU)	Height%	Start time (min)	End time (min)
1	●	VWD1B,Wavelength=254 nm	7.458	30.496	16.227	2.399	12.34	7.144	7.898
2	⬟	VWD1B,Wavelength=254 nm	13.016	157.438	83.773	17.041	87.66	12.551	13.652

4 hr

#	Name	Signal description	RT (min)	Area (mAU·s)	Area%	Height (mAU)	Height%	Start time (min)	End time (min)
1	●	VWD1B,Wavelength=254 nm	7.559	38.324	16.111	3.332	13.29	7.269	8.109
2	⬟	VWD1B,Wavelength=254 nm	13.023	199.553	83.889	21.737	86.71	12.589	13.676

24 hr

#	Name	Signal description	RT (min)	Area (mAU·s)	Area%	Height (mAU)	Height%	Start time (min)	End time (min)
1	●	VWD1B,Wavelength=254 nm	7.404	34.049	16.852	2.728	12.76	7.102	8.075
2	◆	VWD1B,Wavelength=254 nm	10.391	12.969	6.419	1.619	7.57	10.270	10.619
3	▲	VWD1B,Wavelength=254 nm	11.573	8.587	4.250	0.999	4.67	11.470	11.797
4	⬟	VWD1B,Wavelength=254 nm	13.009	146.439	72.479	16.032	74.99	12.775	13.615

$$\%\ \text{Degradation} = \left| \frac{(\text{Area Ratio}_{\text{initial}}) - (\text{Area Ratio}_{\text{final}})}{(\text{Area Ratio}_{\text{initial}})} \right| \times 100\%$$

$$\left| \frac{(157.438/30.496) - (199.553/38.324)}{(157.438/30.496)} \right| \times 100\% \quad = \sim 1\%\ \text{degradation after 4 hr}$$

$$\left| \frac{(157.438/30.496) - (146.439/34.049)}{(157.438/30.496)} \right| \times 100\% \quad = \sim 17\%\ \text{degradation after 24 hr}$$

Figure S2. Stability of Lac-CDLP 6e in the presence of N-acetyllysine methyl ester. **A**) Representative HPLC chromatograms of Lac-CDLP **6e** incubated with N-acetyllysine methyl ester at 0, 4, and 24 h. The parent Lac-CDLP **6e** peak is indicated by the blue pentagon, caffeine internal standard by the red circle, and LC-MS-verified degradation products by the yellow diamond and green triangle. **B**) Peak integration tables and internal standard-normalized calculations show limited degradation after 4 h and increased degradation by 24 h, corresponding to approximately 1% and 17% degradation, respectively.

Supplementary Figure S3

A

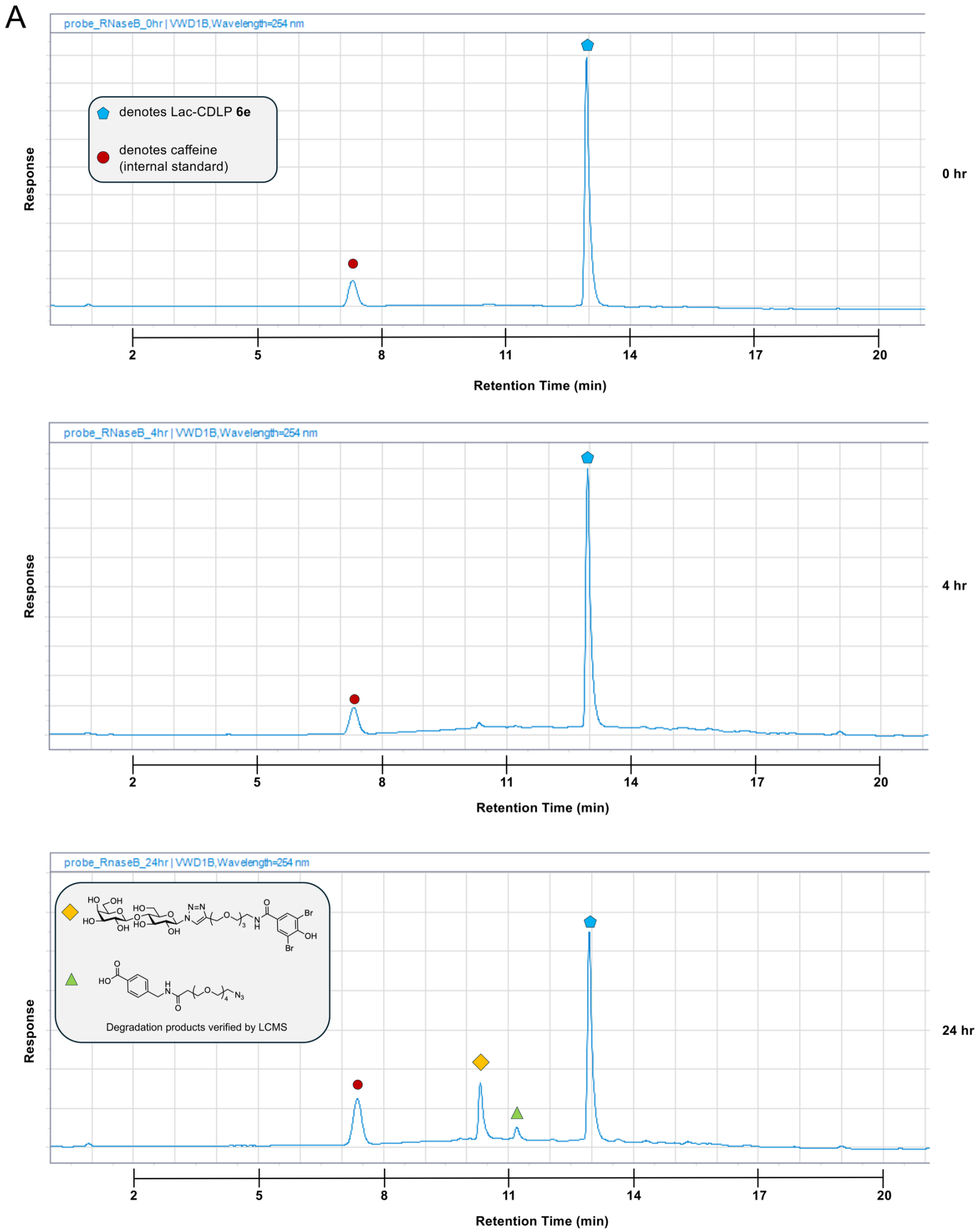

B

#	Name	Signal description	RT (min)	Area (mAU·s)	Area%	Height (mAU)	Height%	Start time (min)	End time (min)
1	●	VWD1B,Wavelength=254 nm	7.313	33.910	15.695	2.321	9.45	7.000	7.790
2	⬟	VWD1B,Wavelength=254 nm	12.954	182.151	84.305	22.239	90.55	12.487	13.563

0 hr

#	Name	Signal description	RT (min)	Area (mAU·s)	Area%	Height (mAU)	Height%	Start time (min)	End time (min)
1	●	VWD1B,Wavelength=254 nm	7.384	34.174	23.625	2.323	14.15	7.006	7.803
2	◆	VWD1B,Wavelength=254 nm	10.335	22.518	15.567	2.880	17.55	10.199	10.743
3	▲	VWD1B,Wavelength=254 nm	11.208	3.854	2.665	0.577	3.51	11.111	11.358
4	⬟	VWD1B,Wavelength=254 nm	12.949	84.102	58.143	10.633	64.79	12.799	13.446

24 hr

$$\% \text{ Degradation} = \left| \frac{(\text{Area Ratio}_{\text{initial}}) - (\text{Area Ratio}_{\text{final}})}{(\text{Area Ratio}_{\text{initial}})} \right| \text{X } 100\%$$

$$\left| \frac{(182.151/33.910) - (84.102/34.174)}{(182.151/33.910)} \right| \text{X } 100\% \quad = \text{~}54\% \text{ degradation after 24 hr}$$

C

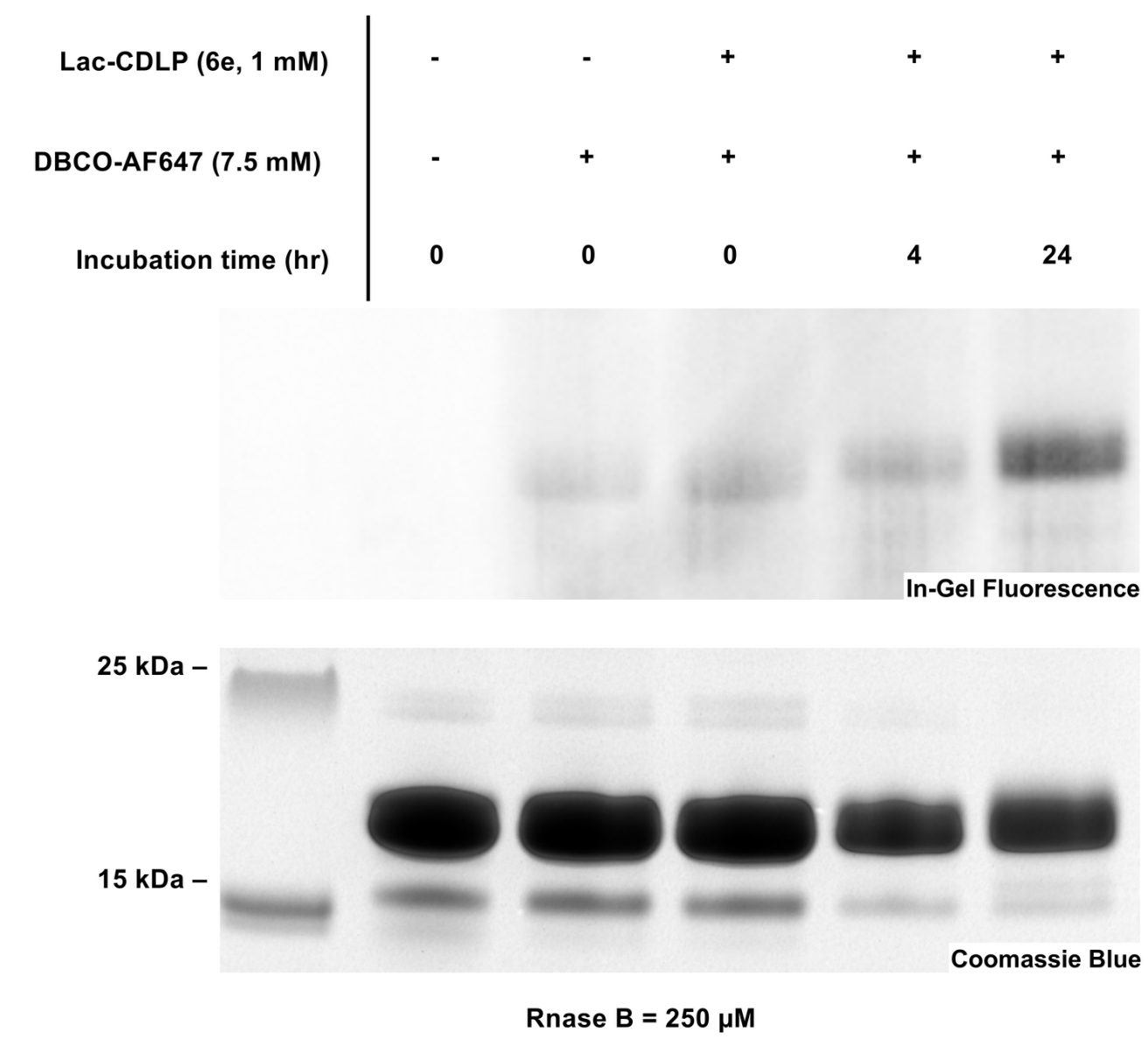

Figure S3. Stability and protein labeling of Lac-CDLP **6e** in the presence of RNase B. **A**) Representative RP-HPLC chromatograms of Lac-CDLP **6e** incubated with RNase B in PBS at 37 °C for 0, 4, and 24 h. Caffeine was included as an internal standard. The parent Lac-CDLP **6e** peak is indicated by the blue pentagon, caffeine by the red circle, and LC-MS-verified degradation products by the yellow diamond and green triangle. Over time, the parent Lac-CDLP 6e peak decreased, and additional product peaks appeared, indicating consumption/degradation of the probe under protein-containing conditions. **B**) Quantification of parent Lac-CDLP **6e** loss by internal-standard-normalized RP-HPLC peak integration. Percent degradation was calculated from the parent Lac-CDLP/caffeine peak-area ratio at the indicated time points. Under these conditions, Lac-CDLP **6e** showed approximately 54% degradation/consumption after 24 h in the presence of RNase B. **C**) In-gel fluorescence analysis of RNase B labeling by Lac-CDLP **6e**. Aliquots from the same incubation were treated with DBCO-AF647 and analyzed by SDS-PAGE, followed by in-gel fluorescence imaging and Coomassie staining. Low RNase B-associated fluorescence increased over time in probe-treated samples, consistent with covalent protein labeling by the azide-bearing acyl-transfer product. Coomassie staining shows RNase B loading.

Supplementary Figure S4

A

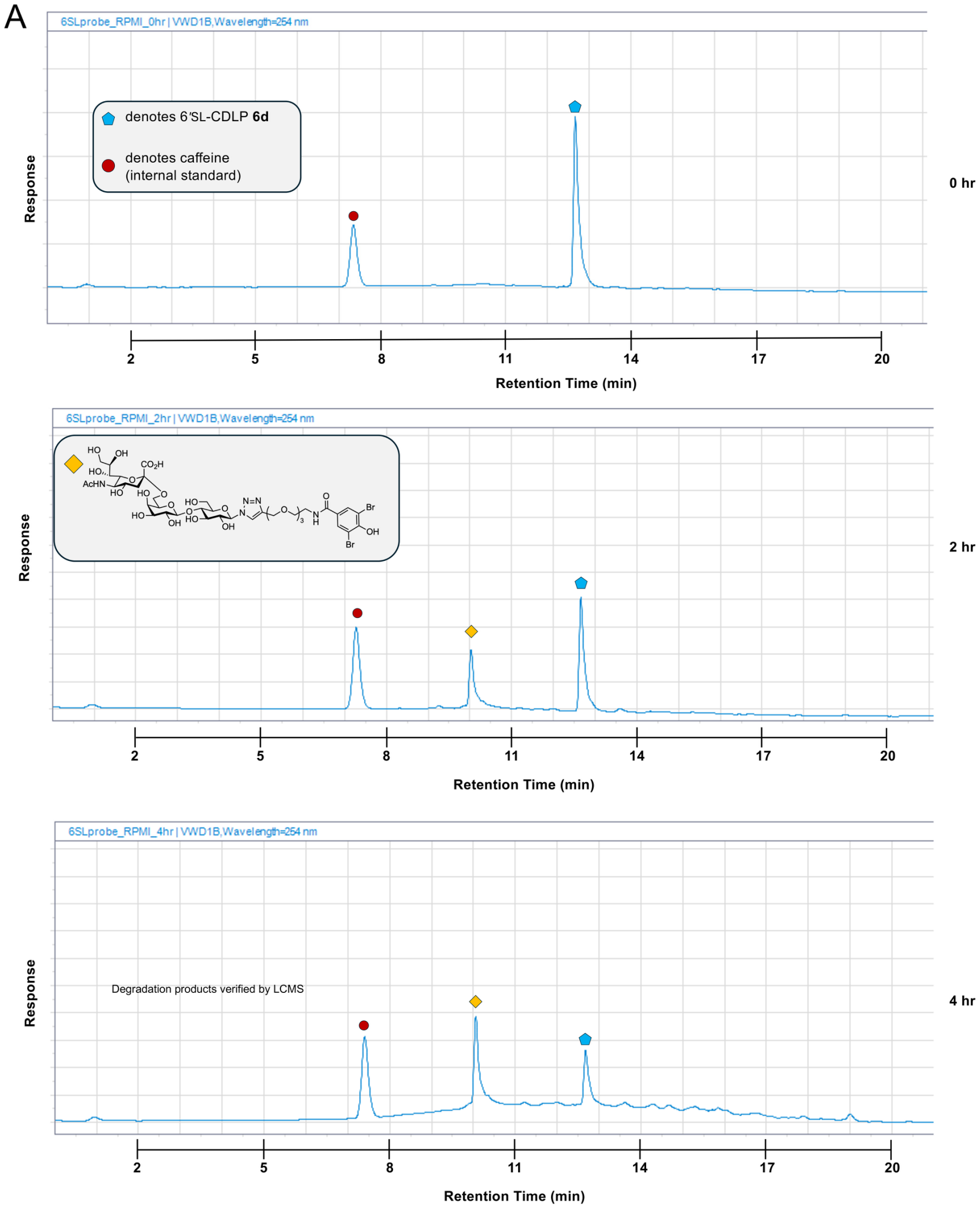

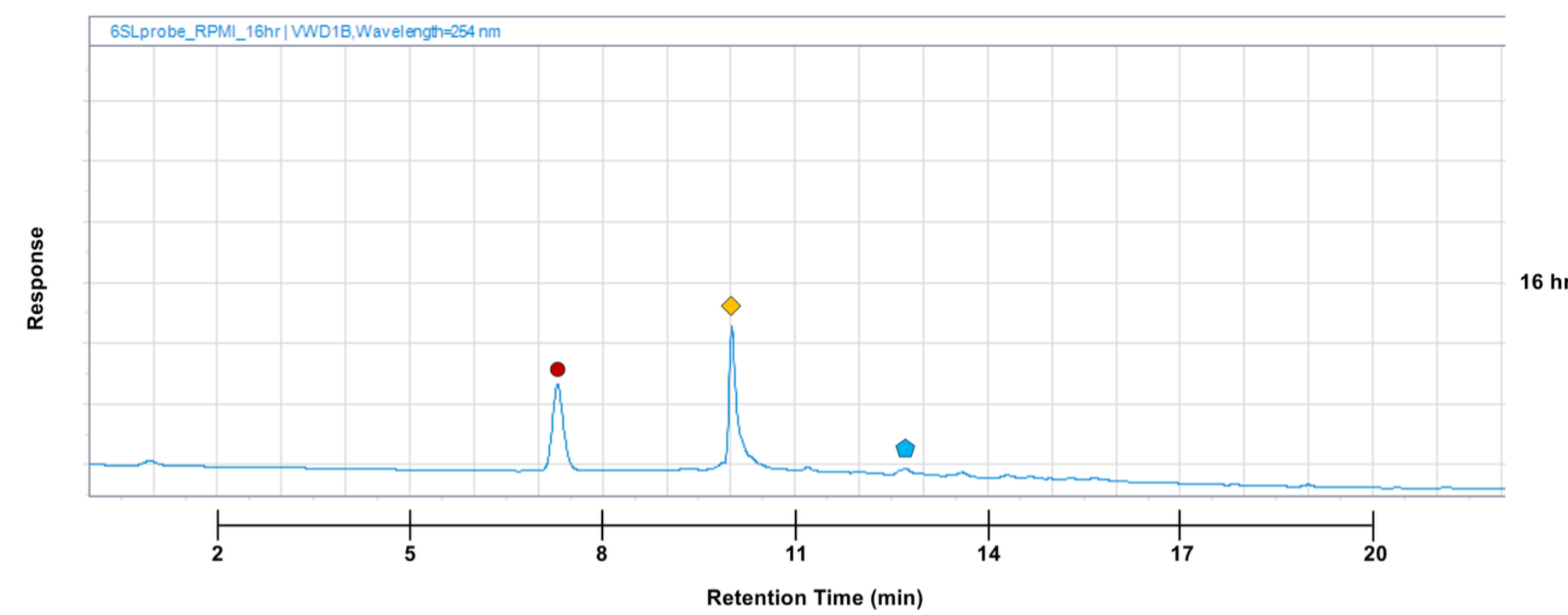

B

0 hr

#	Name	Signal description	RT (min)	Area (mAU·s)	Area%	Height (mAU)	Height%	Start time (min)	End time (min)
1	● (red)	VWD1B,Wavelength=254 nm	7.357	33.442	31.552	2.815	26.64	7.081	7.844
2	⬟ (blue)	VWD1B,Wavelength=254 nm	12.680	72.549	68.448	7.750	73.36	12.467	13.427

4 hr
73% Degradation

#	Name	Signal description	RT (min)	Area (mAU·s)	Area%	Height (mAU)	Height%	Start time (min)	End time (min)
1	● (red)	VWD1B,Wavelength=254 nm	7.417	33.855	38.573	2.962	35.62	7.152	7.799
2	◆ (yellow)	VWD1B,Wavelength=254 nm	10.069	34.516	39.326	3.332	40.08	9.539	10.766
3	⬟ (blue)	VWD1B,Wavelength=254 nm	12.693	19.397	22.101	2.021	24.30	12.439	13.372

Figure S4. Degradation of 6′SL-CDLP 6d in RPMI medium. **A**) Representative HPLC chromatograms of 6′SL-CDLP **6d** incubated in RPMI medium over time. The parent 6′SL-CDLP **6d** peak is indicated by the blue pentagon, caffeine internal standard by the red circle, and LC-MS-verified consumption/degradation products by the yellow diamond. The parent probe decreases rapidly, accompanied by the formation of consumption/degradation products. **B**) Peak integration tables show substantial loss of 6′SL-CDLP **6d** in RPMI, with approximately 73% consumption/degradation by 4 h based on internal standard-normalized peak areas.

Supplementary Figure S5

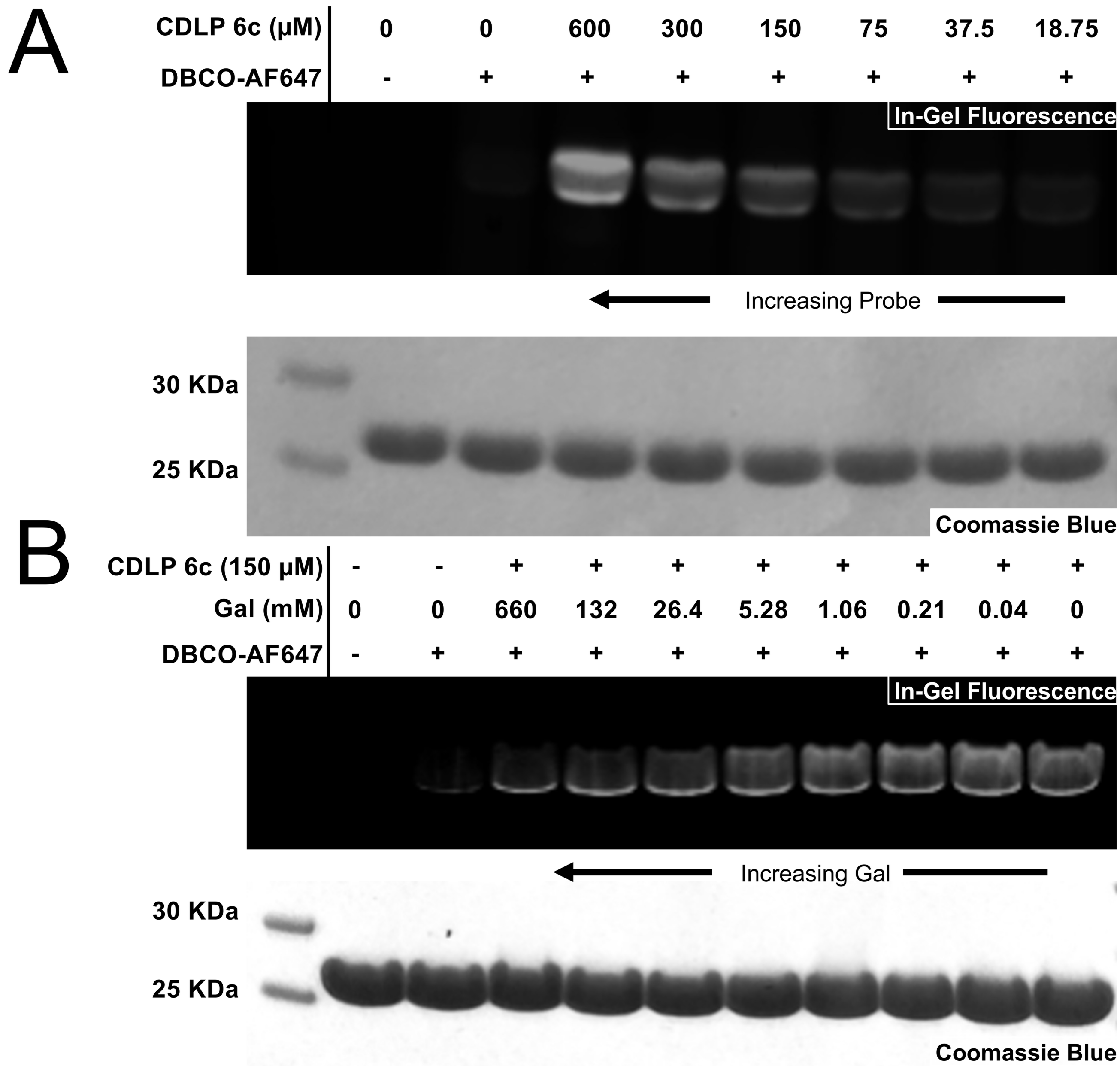

Figure S5. CDLPs covalently capture the PNA–Gal interaction in vitro. **A**) Concentration-dependent labeling of PNA (75 μM final lectin concentration) by Gal-CDLP **6c** (0–600 μM). Fluorescence increases with Gal-CDLP **6c** concentration, while Coomassie staining shows comparable protein loading. **B**) Free galactose (0–660 mM) competitively inhibits PNA labeling (PNA, 75 μM; **6c**, 150 μM), supporting carbohydrate-recognition-dependent capture.

Supplementary Figure S6

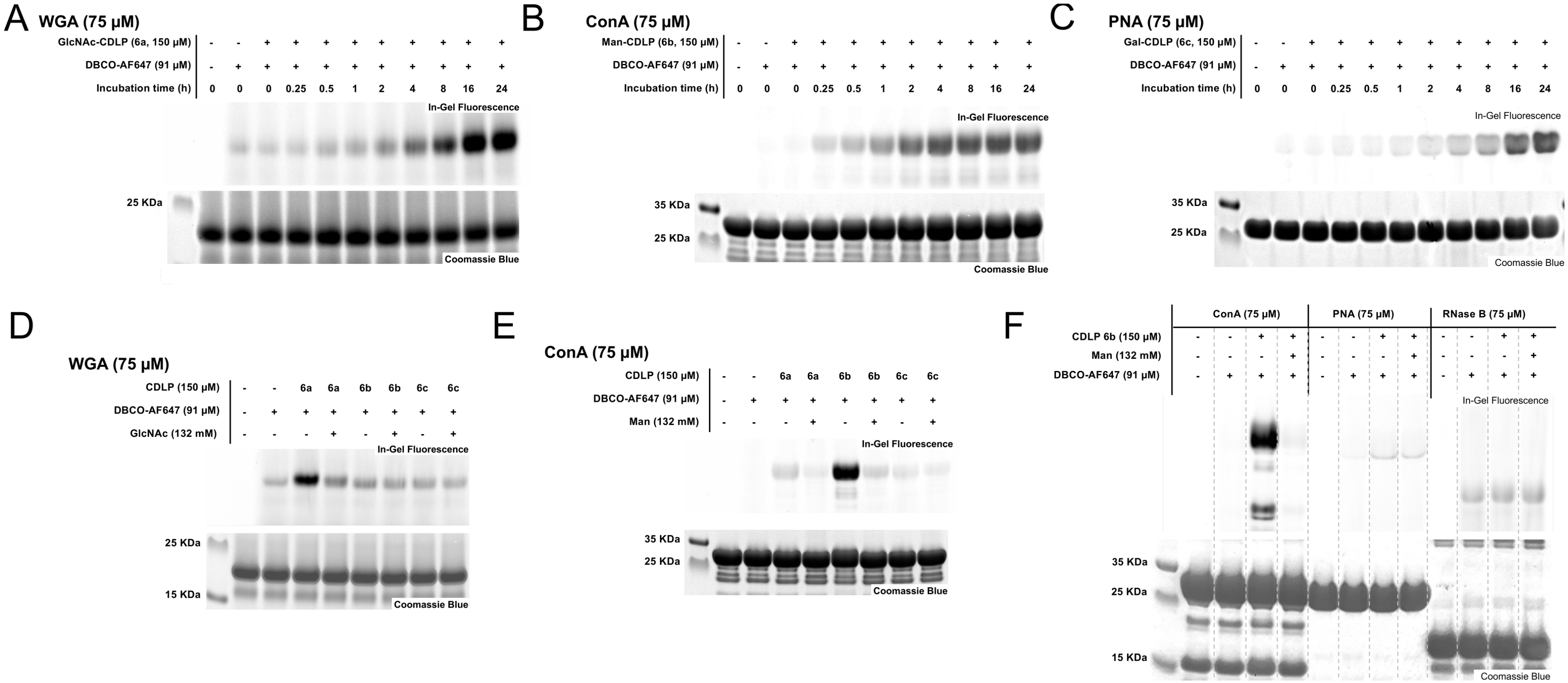

Figure S6. Purified-protein benchmarking of time-dependent and ligand-dependent CDLP labeling. **A**–**C**) Time-dependent labeling of purified lectins by matched CDLP probes. WGA, ConA, or PNA was incubated with GlcNAc-CDLP **6a**, Man-CDLP **6b**, or Gal-CDLP **6c**, respectively, at 37 °C for the indicated times. Aliquots were then treated with DBCO-AF647 and analyzed by SDS-PAGE/in-gel fluorescence, followed by Coomassie staining. Matched lectin–probe pairs showed time-dependent labeling, with ConA/Man-CDLP **6b** producing strong signal by approximately 4 h and WGA/GlcNAc-CDLP **6a** and PNA/Gal-CDLP **6c** showing slower signal accumulation under these conditions. **D**–**E**) Matched and mismatched CDLP labeling of WGA and ConA. WGA or ConA was incubated with GlcNAc-CDLP **6a**, Man-CDLP **6b**, or Gal-CDLP **6c** in the absence or presence of excess cognate monosaccharide competitor. WGA showed preferential labeling by GlcNAc-CDLP **6a**, while ConA showed preferential labeling by Man-CDLP **6b**. Labeling of the matched lectin–probe pairs was reduced by the corresponding free monosaccharide competitor. **F**) Protein-selectivity control for Man-CDLP **6b**. ConA, PNA, or RNase B was treated with Man-CDLP **6b** in the absence or presence of excess mannose. Man-CDLP **6b** labeled ConA more strongly than noncognate PNA or the non-lectin glycoprotein RNase B, and ConA labeling was reduced by free mannose. Coomassie staining shows protein loading.

Supplementary Figure S7

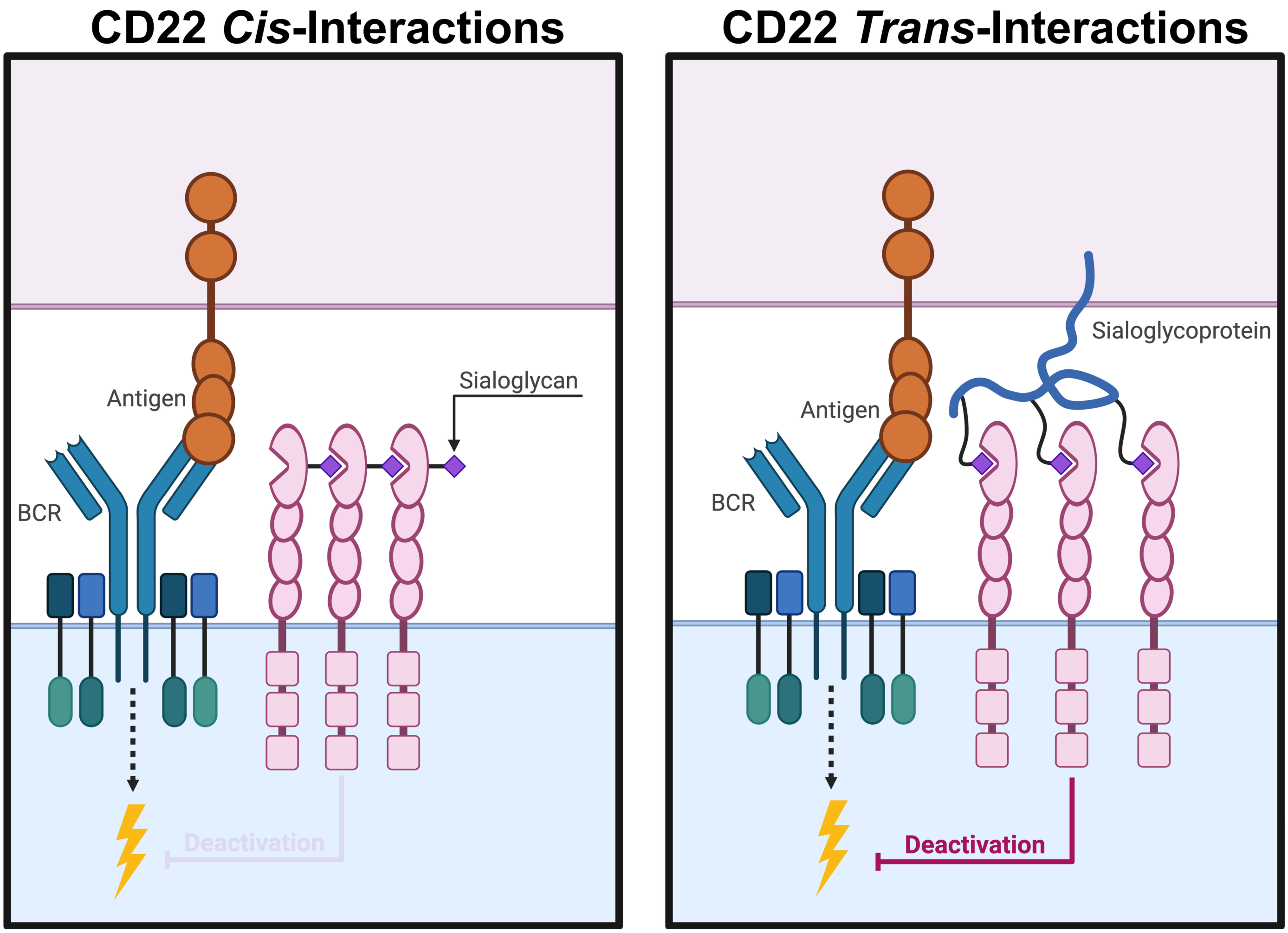

Figure S7. CD22 cis-masking and trans engagement shape inhibitory control of BCR signaling. Although CD22 binds weakly to monomeric α2,6-sialyllactose ($K_D \approx 300$ μM), its lectin site is typically masked by extensive cis-interactions with sialoglycan-decorated proteins on the same B-cell surface (left). Multivalent trans sialylated ligands (e.g., sialoglycoproteins) can compete with cis binding to engage CD22 and promote BCR deactivation (right).

Supplementary Figure S8

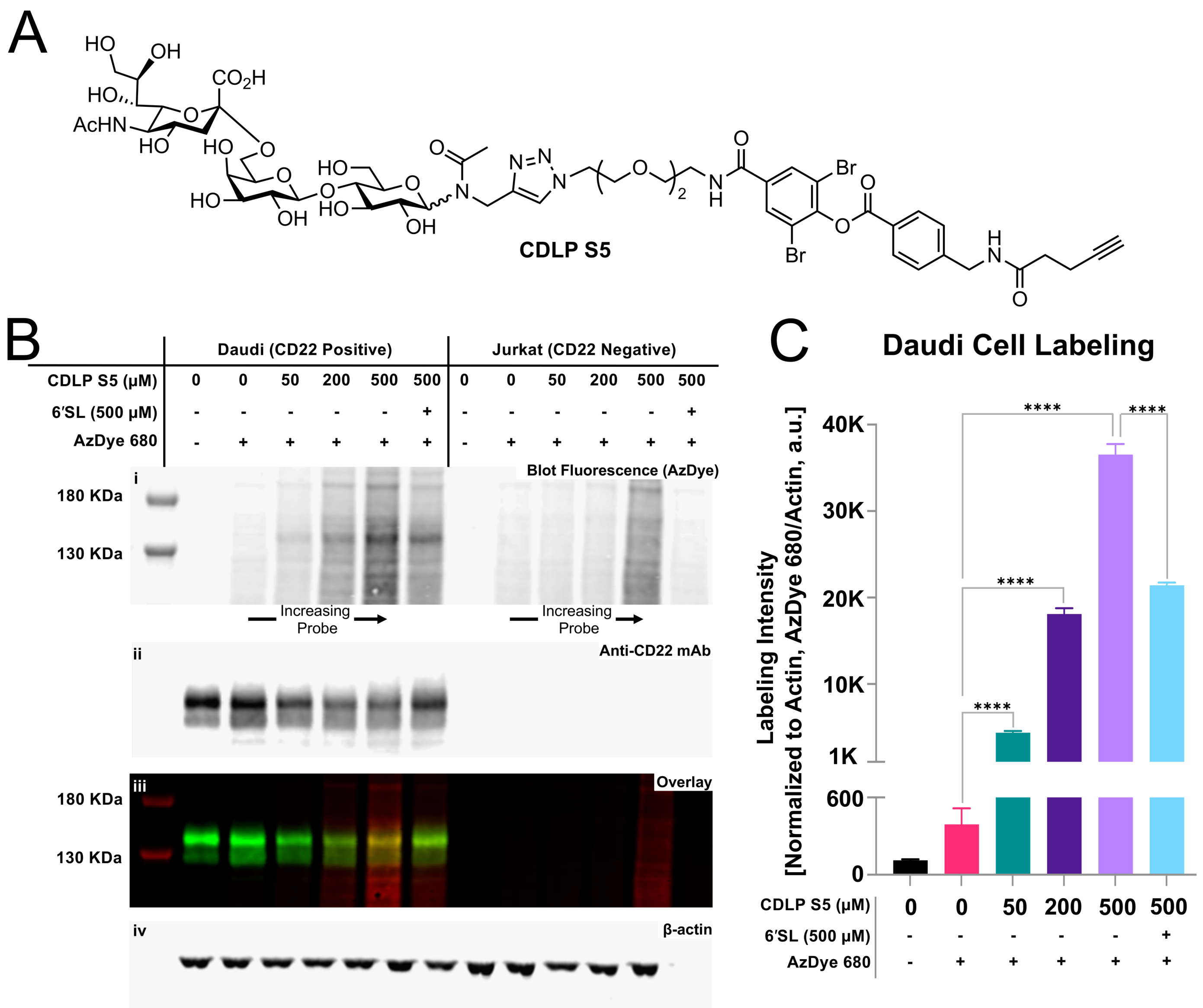

Figure S8. Live-cell labeling of CD22 by alkyne-bearing 6′SL-CDLP analog **S5**. A) Structure of **S5**. B) Daudi ($CD22^+$) and Jurkat ($CD22^-$) cells were treated with **S5** at the indicated concentrations, functionalized with AzDye 680 azide by CuAAC, and analyzed by fluorescence imaging and anti-CD22 immunoblotting. In the competition condition, cells were treated with **S5** (500 μM) in the presence of free 6′SL competitor (500 μM); this condition corresponds to the final lane in each cell-line series. The CDLP-derived fluorescent signal overlaps with CD22 in Daudi cells and is reduced by free 6′SL competitor, while signal is reduced or absent at the corresponding CD22 region in Jurkat cells, supporting 6′SL-dependent CD22 labeling and handle modularity of the 6′SL-CDLP design. C) Quantification of CD22-associated fluorescence normalized to β-actin, plotted as individual biological replicates with mean ± SEM (n = 3). Data were analyzed using an ordinary one-way ANOVA followed by Dunnett's multiple-comparisons test; $F(5,12) = 1735$, $p < 0.0001$. For all panels: *$p < 0.05$, **$p < 0.01$, ***$p < 0.001$, ****$p < 0.0001$; ns, not significant.

Supplementary Figure S9

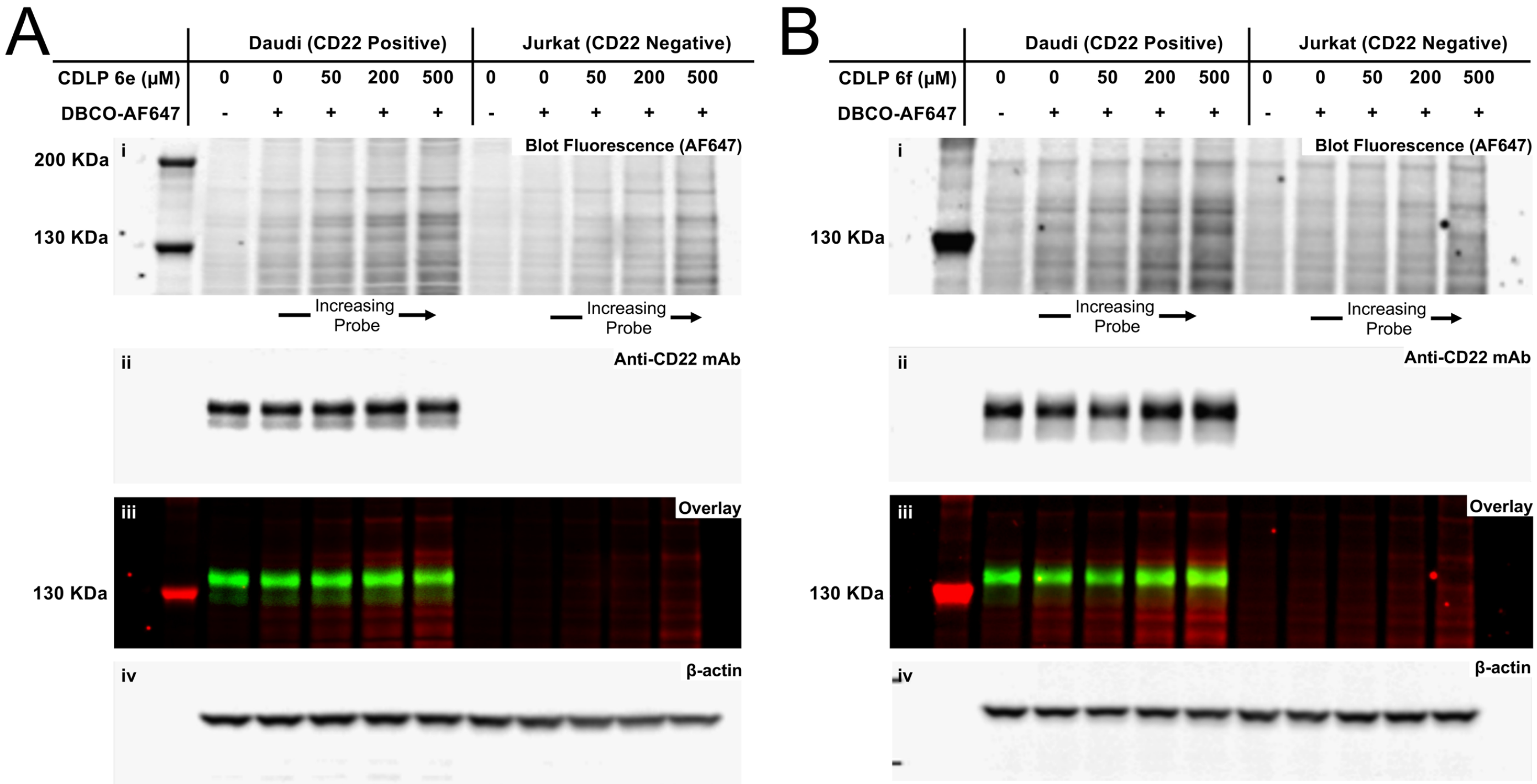

Figure S9. Lactose-CDLP **6e** and 3′SL-CDLP **6f** show minimal CD22 labeling. Daudi ($CD22^{+}$) and Jurkat ($CD22^{-}$) cells were treated with increasing concentrations (0–500 μM) of lactose-CDLP **6e** (A) or 3′SL-CDLP **6f** (B) and visualized using DBCO-AF647. AF647 signal (i), anti-CD22 immunoblot (ii), and the merged overlay (iii) reveal no robust CD22-associated labeling in Daudi cells and minimal CD22-region signal in Jurkat cells under the same conditions; actin is shown as a loading control (iv), supporting ligand-dependent capture.

Supplementary Figure S10

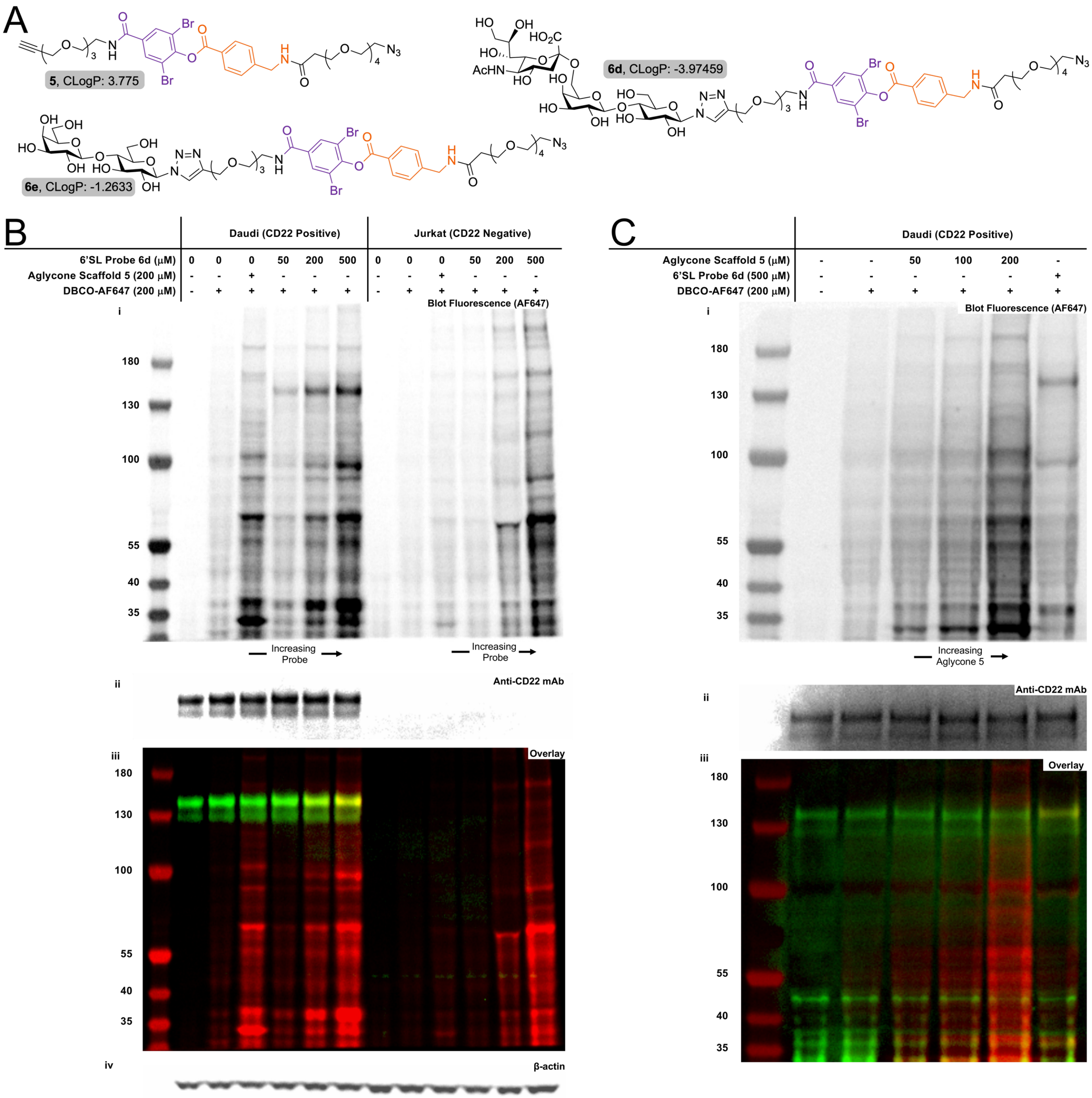

Figure S10. Aglycone scaffold control for live-cell CD22 labeling. **A**) Structures and calculated logP values for aglycone scaffold **5**, 6′SL-CDLP **6d**, and Lac-CDLP **6e**. ClogP values were calculated using ChemDraw. Removal of the glycan substantially increases calculated lipophilicity, distinguishing scaffold **5** from the glycan-bearing CDLPs. **B**) Daudi CD22$^+$ and Jurkat CD22$^-$ cells were treated with 6′SL-CDLP **6d** at the indicated concentrations or aglycone scaffold **5**, functionalized with DBCO-AF647, and analyzed by SDS-PAGE/in-gel fluorescence, anti-CD22 immunoblotting, overlay, and β-actin loading control. Scaffold **5** produced broad cellular labeling, including in CD22-negative Jurkat cells, rather than a discrete CD22-associated signal. C) Daudi CD22$^+$ cells were treated with increasing concentrations of aglycone scaffold **5** or 6′SL-CDLP **6d**, functionalized with DBCO-AF647, and analyzed by in-gel fluorescence, anti-CD22 immunoblotting, and overlay. Scaffold **5** produced broad, concentration-dependent labeling that did not selectively overlap with CD22. Broad background labeling was also observed to varying extents in glycan-CDLP-treated samples, consistent with contribution from the aryl-ester/linker core. These data support interpretation of scaffold **5** as a scaffold-background control rather than a ligand-matched negative control, and support the use of glycan-bearing Lac-CDLP **6e** and 3′SL-CDLP **6f** as more appropriate ligand controls for sialic-acid-dependent CD22 capture.

Supplementary Figure S11

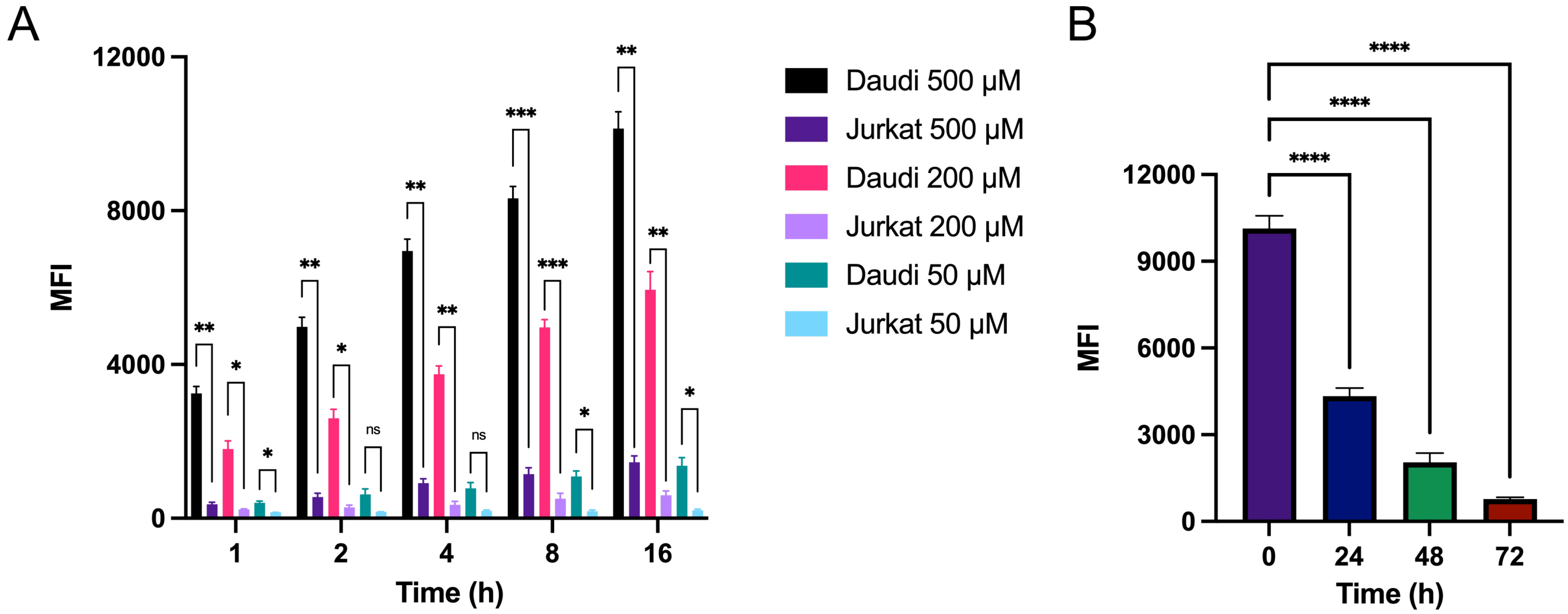

Figure S11. Flow-cytometry analysis of 6′SL-CDLP labeling kinetics and post-wash persistence. **A**) Daudi and Jurkat cells were incubated with 6′SL-CDLP at the indicated concentrations for 1–16 h, functionalized with DBCO-AF647, and analyzed by flow cytometry. Labeling is reported as MFI with individual biological replicates and mean ± SEM (n = 3). Data were analyzed using a two-way repeated-measures ANOVA with Geisser–Greenhouse correction, followed by Tukey's multiple-comparisons test within each time point; time, condition, and time × condition effects were significant, all $p < 0.0001$. **B**) Daudi cells were labeled with 6′SL-CDLP, washed, returned to probe-free medium, and analyzed at 0, 24, 48, and 72 h after washout. MFI is shown as individual biological replicates with mean ± SEM (n = 4). Data were analyzed by one-way ANOVA followed by Dunnett's test versus 0 h; all post-wash time points were significantly reduced, adjusted $p < 0.0001$. For all panels: *$p < 0.05$, **$p < 0.01$, ***$p < 0.001$, ****$p < 0.0001$; ns, not significant.

Supplementary Figure S12

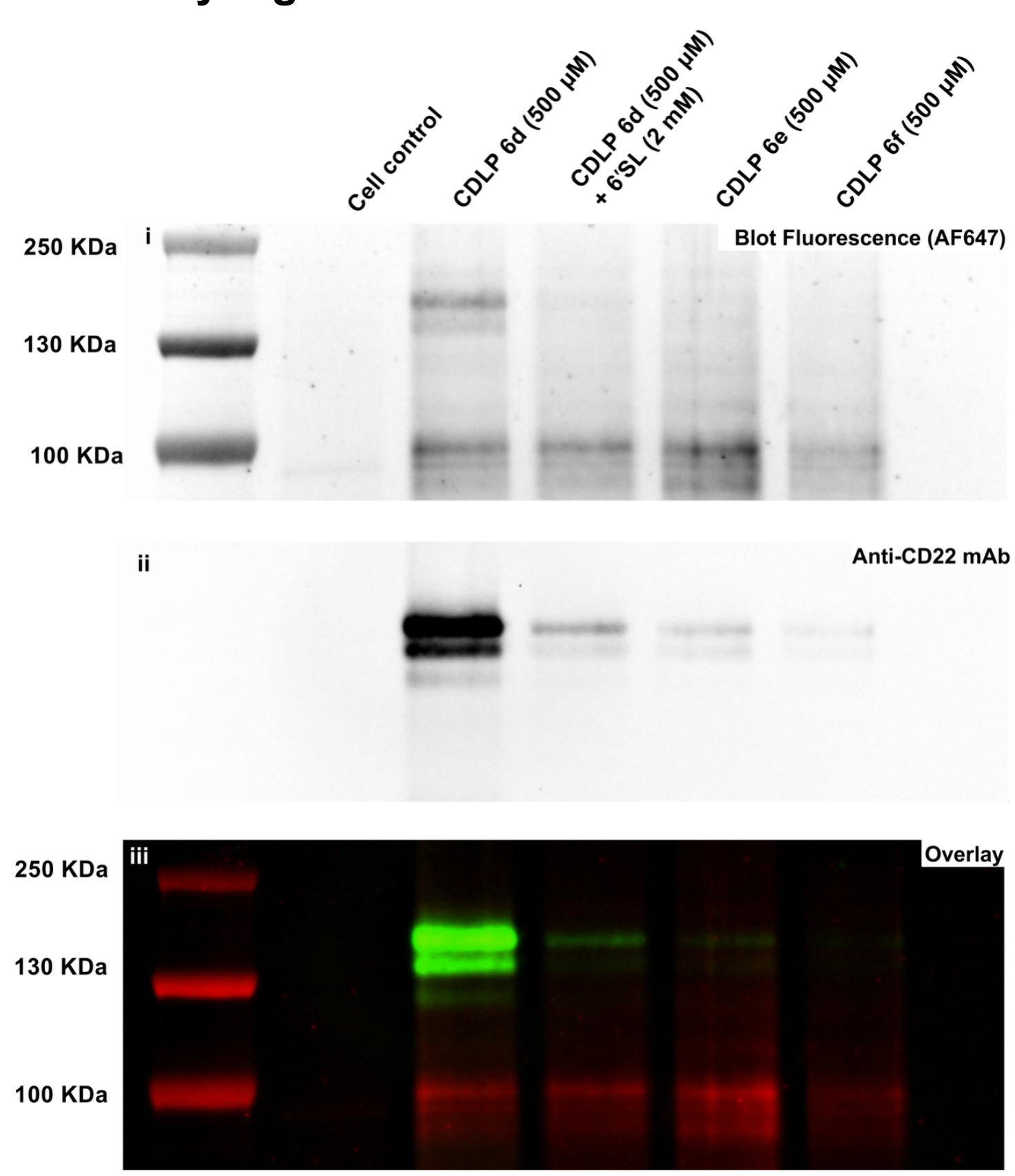

Figure S12. Streptavidin-enrichment validation of CD22 capture by 6′SL-CDLP **6d** (500 μM) in Daudi cells. Daudi cells were treated with the indicated CDLP probes, functionalized with a desthiobiotin affinity tag, enriched on streptavidin beads, and analyzed by SDS-PAGE/immunoblotting. Samples included a no-probe cell control, 6′SL-CDLP **6d** (500 μM), 6′SL-CDLP **6d** (500 μM) plus excess free 6′SL competitor (2 mM), Lac-CDLP **6e** (500 μM), and 3′SL-CDLP **6f** (500 μM). i) Streptavidin-AF647 detection of enriched desthiobiotin-tagged CDLP-labeled proteins. ii) Anti-CD22 immunoblot of enriched fractions. iii) Overlay of AF647 and anti-CD22 signals. CD22 recovery was strongest with 6′SL-CDLP **6d** (500 μM) and decreased with free 6′SL competitor; Lac-CDLP **6e** (500 μM) and 3′SL-CDLP **6f** (500 μM) showed weaker CD22 recovery under the same conditions. AF647 signal outside the CD22 region indicates that total CDLP labeling/enrichment includes additional cellular proteins.

Supplementary Figure S13

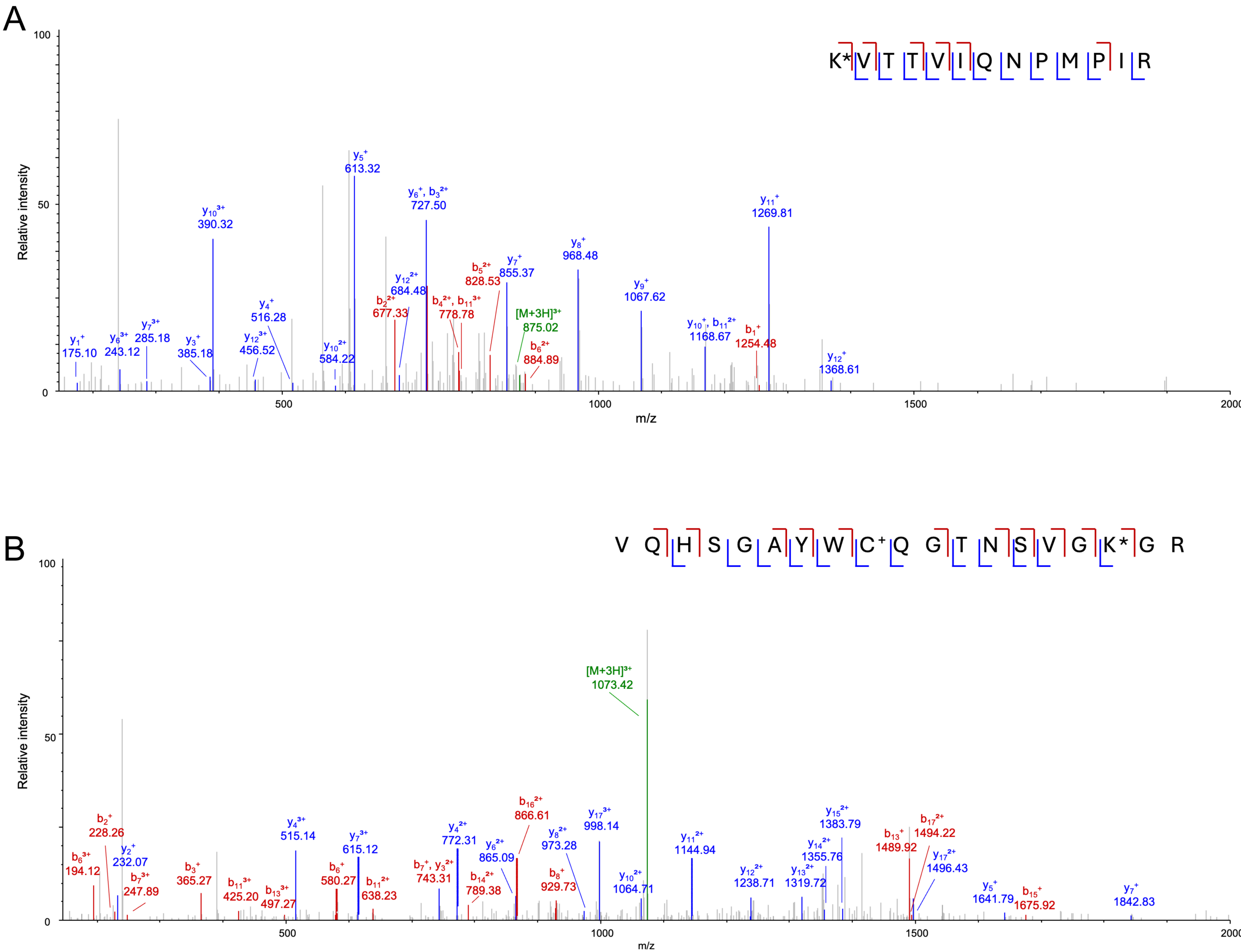

Figure S13. Annotated MS/MS spectra of CDLP-modified CD22 peptides. **A**) MS/MS spectrum of K*VTTVIQNPMPIR, with matched b- and y-ion fragments labeled according to the peptide sequence. The asterisk indicates the CDLP-derived acyl modification on the N-terminal Lys residue; the modified b1 ion at m/z 1254.48 supports localization of the modification. **B**) MS/MS spectrum of VQHSGAYWC+QGTNSVGK*GR. Matched b- and y-ion fragments support peptide assignment and localization of the CDLP-derived acyl modification to K*. The plus sign denotes carbamidomethylated cysteine introduced during sample preparation. These spectra were obtained from a dedicated CD22-focused 6d labeling experiment, whereas the comparative chemoproteomics experiment was analyzed at the protein-enrichment level.

Supplementary Figure S14

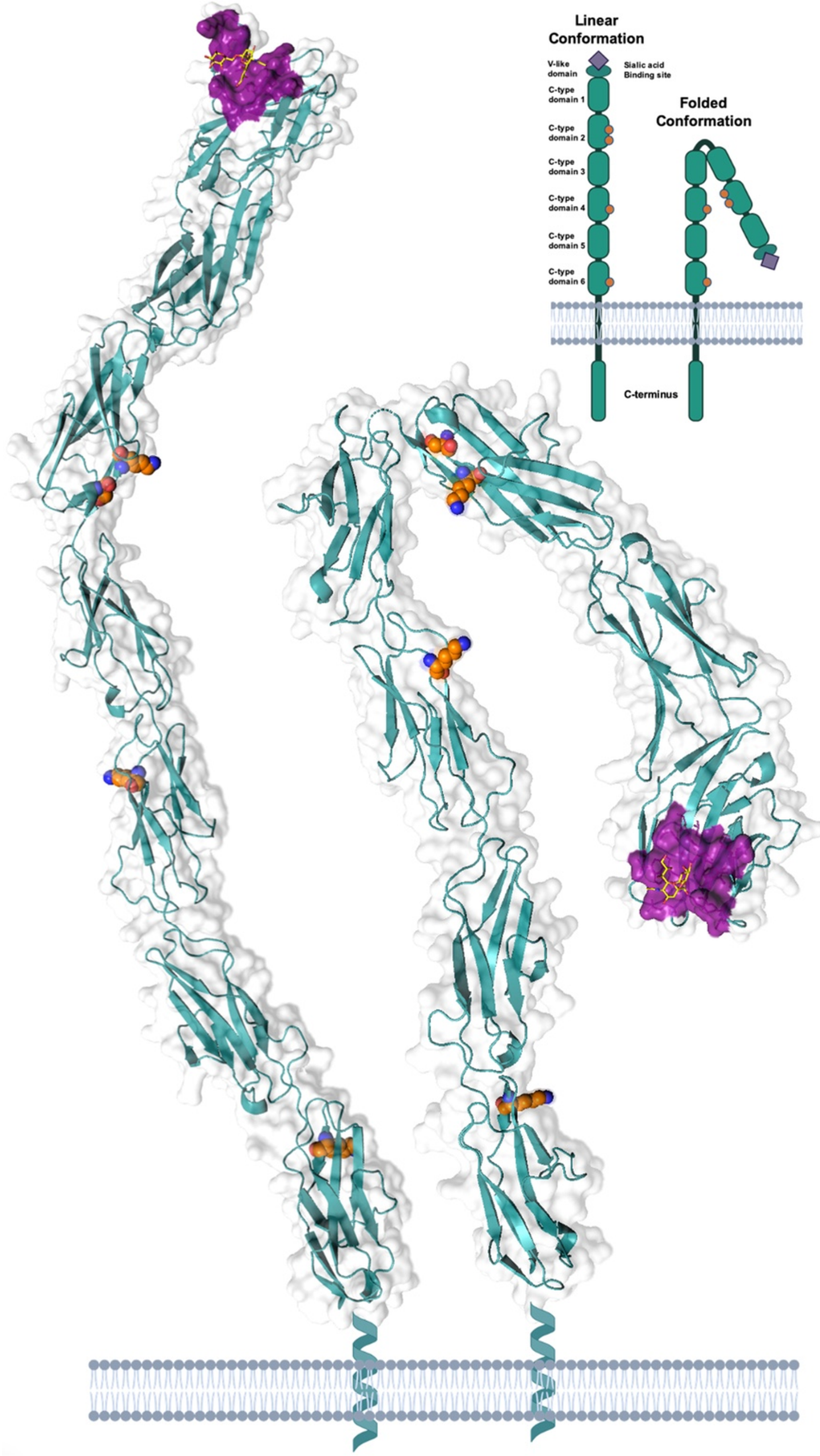

Figure S14. Illustrative mapping of CD22 residues modified by 6′SL-CDLP 6d onto example ectodomain models. An extended (“linear”) model and a folded (“U-shaped”) model of the CD22 extracellular domain are shown oriented relative to the membrane. The sialic acid-binding pocket in the N-terminal V-set domain is highlighted, and residues identified by LC-MS/MS as modified by 6′SL-CDLP 6d (S304, K306, K421, and K667) are shown as spheres. The folded model was generated using Ig-domain hinge geometry based on the strategy described by Bannister et al.[54] These models are provided solely as visual references for the distribution of modified residues on representative CD22 ectodomain arrangements. No structural or conformational conclusions are drawn from these mappings. In particular, the models do not account for glycosylation, intermolecular labeling within CD22 clusters, or the full conformational ensemble of CD22.

Supplementary Figure S15

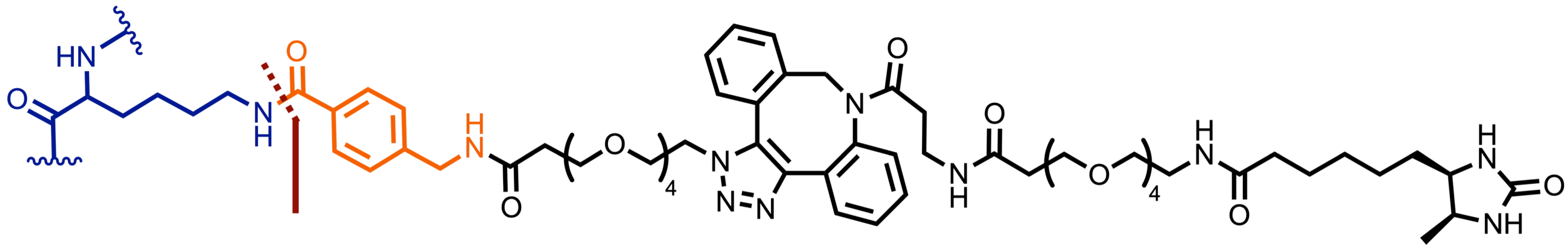

Chemical Formula: $C_{58}H_{79}N_9O_{14}$
Exact Mass: 1125.57
Molecular Weight: 1126.32

Figure S15. Structure and mass of CDLP-adduct post-functionalization with DBCO-PEG4-desthiobiotin (acyl tag + clicked reporter). The monoisotopic exact mass is 1125.57 Da; average molecular weight is 1126.32 Da.

Supplementary Figure S16

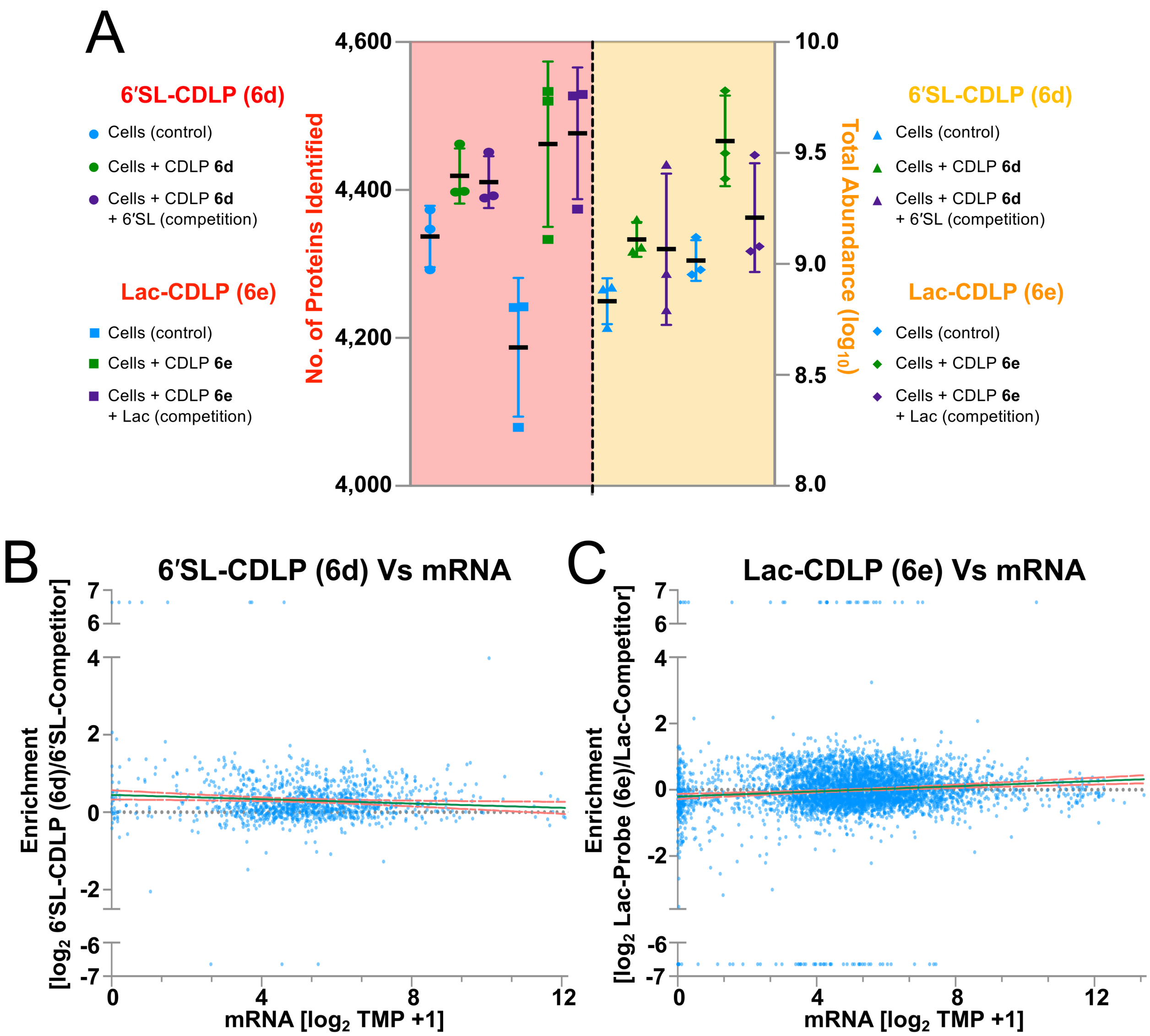

Figure S16. Quality-control assessment of comparative chemoproteomic profiling samples and relationship between probe enrichment and transcript abundance. **A**) Per-run proteome coverage and total abundance for the indicated sample conditions. Protein identifications are plotted on the left y-axis as the number of proteins identified per LC–MS/MS run; total proteomic abundance is plotted on the right y-axis as $\log_{10}$(total protein abundance). Points represent individual biological replicates and bars represent mean ± SD (n = 3). Protein-identification counts clustered around ~4.2–4.5 × 10^3 proteins across control, probe-only, and competition samples for both probe sets, and total-abundance distributions overlapped broadly between conditions, indicating stable LC–MS/MS acquisition and processing. **B,C**) Relationship between transcript abundance and probe enrichment for 6′SL-CDLP 6d (B) and Lac-CDLP 6e (C). Each point represents one protein. Transcript abundance values were obtained from DepMap Public 25Q3. The x-axis shows DepMap gene expression in Daudi cells as $\log_2$(TPM + 1), and the y-axis shows protein enrichment as $\log_2$[(probe)/(probe + corresponding free glycan competitor)]. A value of 0 indicates no enrichment, whereas values of ±1 correspond to two-fold enrichment or depletion. Lines show ordinary least-squares fits with unconstrained intercepts. For 6′SL-CDLP 6d, n = 1,194 proteins, slope = −0.02770, 95% CI = −0.04929 to −0.00611, Pearson r = −0.073, R^2 = 0.0053, p = 0.0120, and residual SD Sy.x = 0.7593. For Lac-CDLP 6e, n = 4,564 proteins, slope = +0.03878, 95% CI = 0.02479 to 0.05278, Pearson r = +0.080, R^2 = 0.0064, $p < 0.0001$, and residual SD Sy.x = 1.047. Although the fitted slopes are statistically nonzero given the large number of proteins analyzed, transcript abundance explains <1% of the variance in probe enrichment in both datasets. Thus, CDLP enrichment is not primarily explained by transcript-inferred protein abundance, supporting competition-sensitive enrichment rather than abundance alone for candidate prioritization.

Supplementary Figure S17

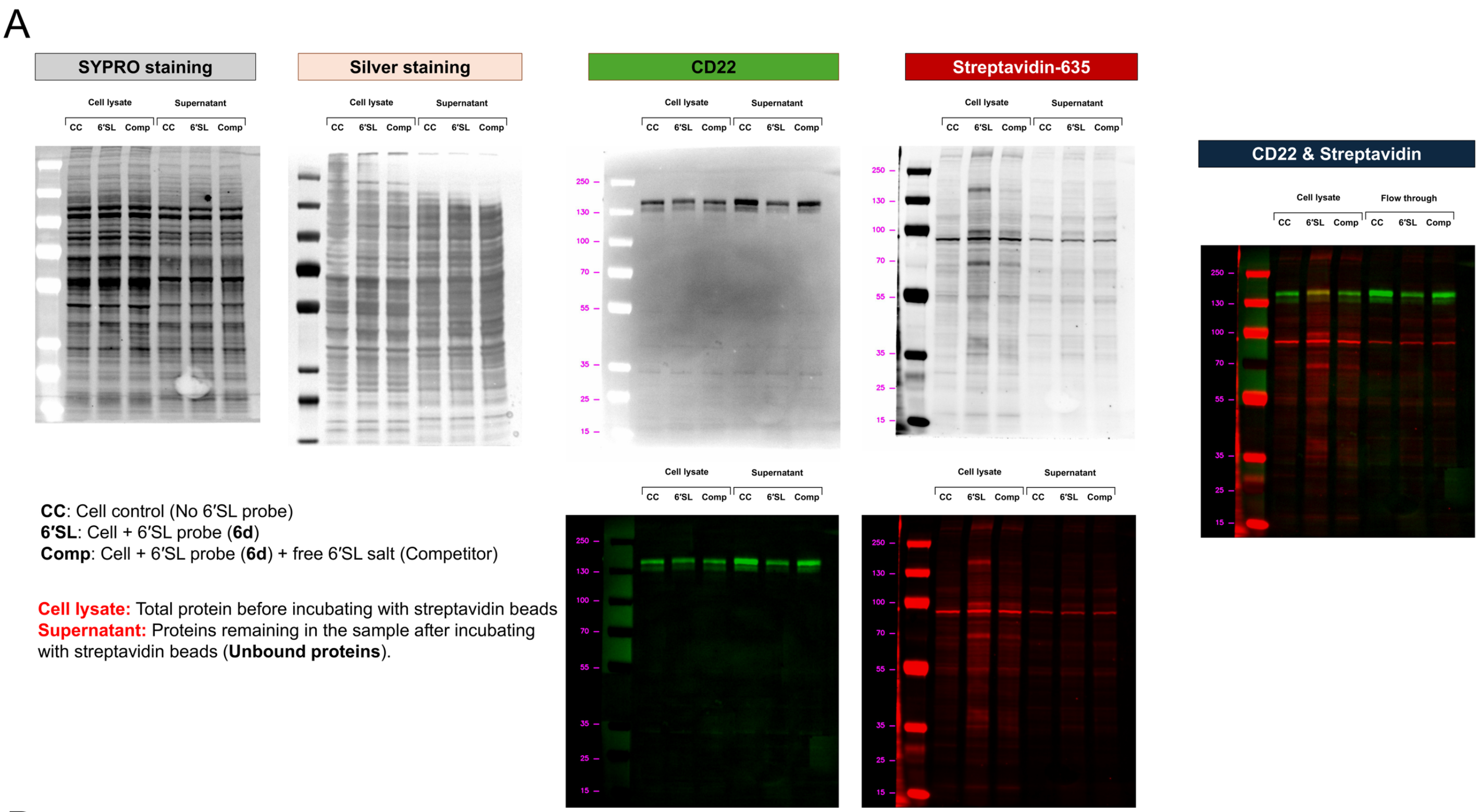

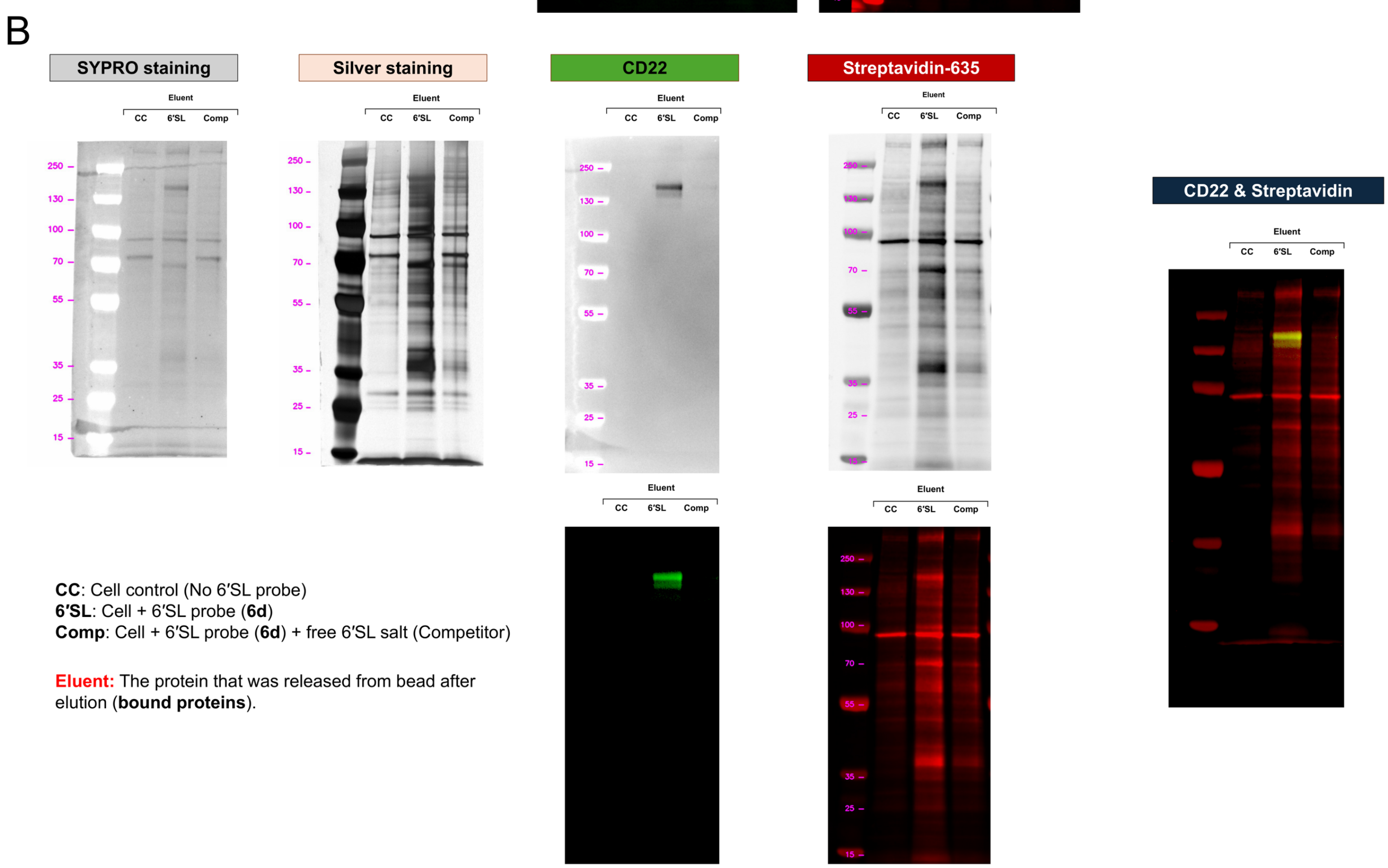

Figure S17. Examples of samples used for proteomics analysis. A) Crude lysate and supernatant samples; B) eluted/enriched fraction.

Supplementary Figure S18

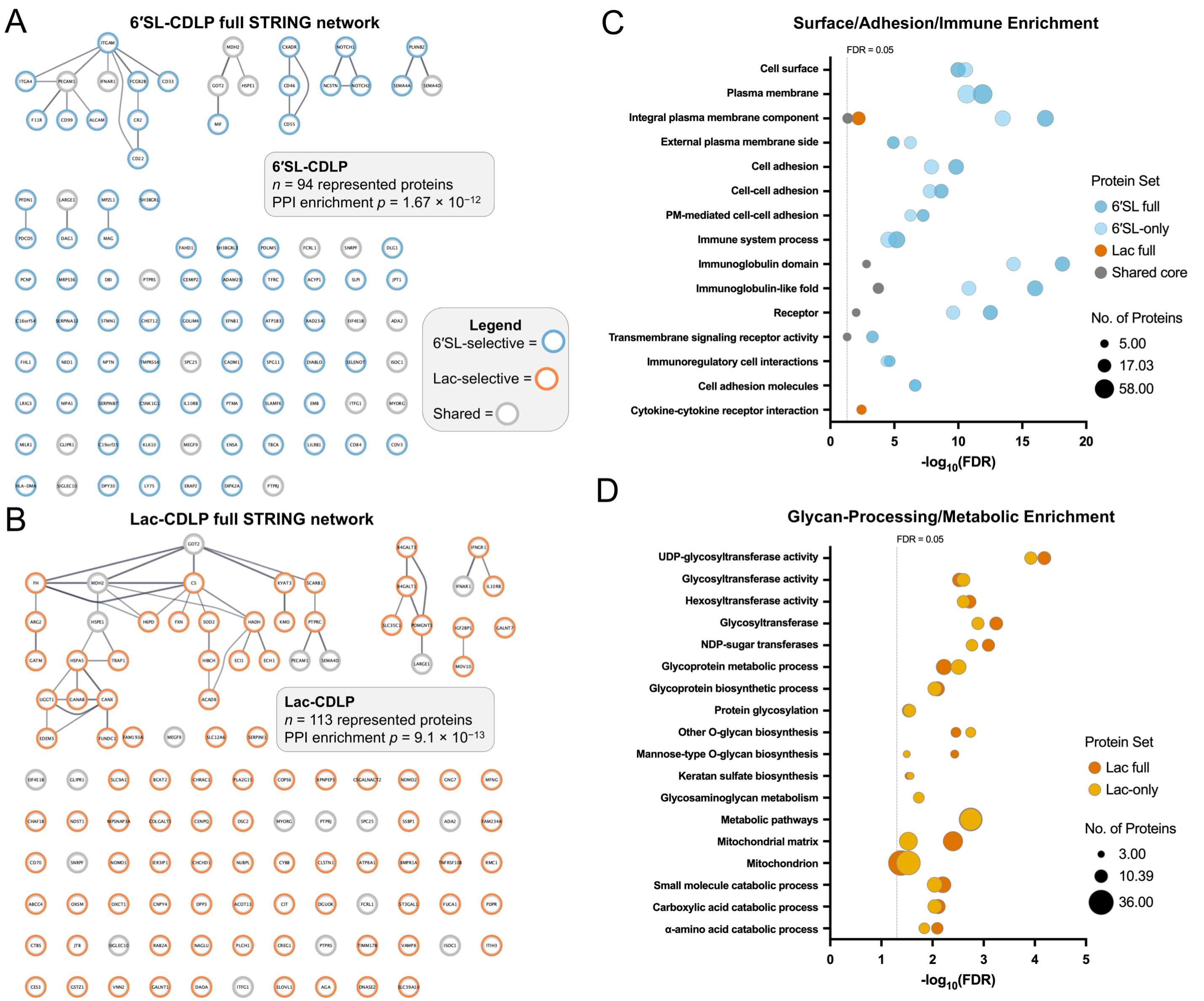

Figure S18. STRING/stringApp analysis of CDLP-captured protein profiles. **A, B**) STRING/stringApp networks of the full 6′SL-CDLP and Lac-CDLP competition-sensitive candidate protein sets generated using the full STRING functional association network, confidence cutoff 0.7, and no added interactors. Colored nodes indicate probe-specific competition-sensitive candidate proteins, and gray nodes indicate proteins shared between the 6′SL-CDLP and Lac-CDLP profiles. PPI enrichment values indicate that both full CDLP profiles are more connected than expected by chance. **C, D**) Dot-strip plots showing selected significantly enriched STRING terms using the CDLP-detected proteome as a custom background. Dot position indicates $-\log_{10}$(FDR), dot size indicates the number of proteins annotated to each term, and dot color indicates protein set. Only terms recovered as significant in the corresponding STRING enrichment export are plotted. The dashed line indicates FDR = 0.05. Surface/adhesion/immune terms were enriched predominantly in the 6′SL-CDLP full and 6′SL-only profiles, whereas glycan-processing and metabolic terms were enriched predominantly in the Lac-CDLP full

Supplementary Figure S19

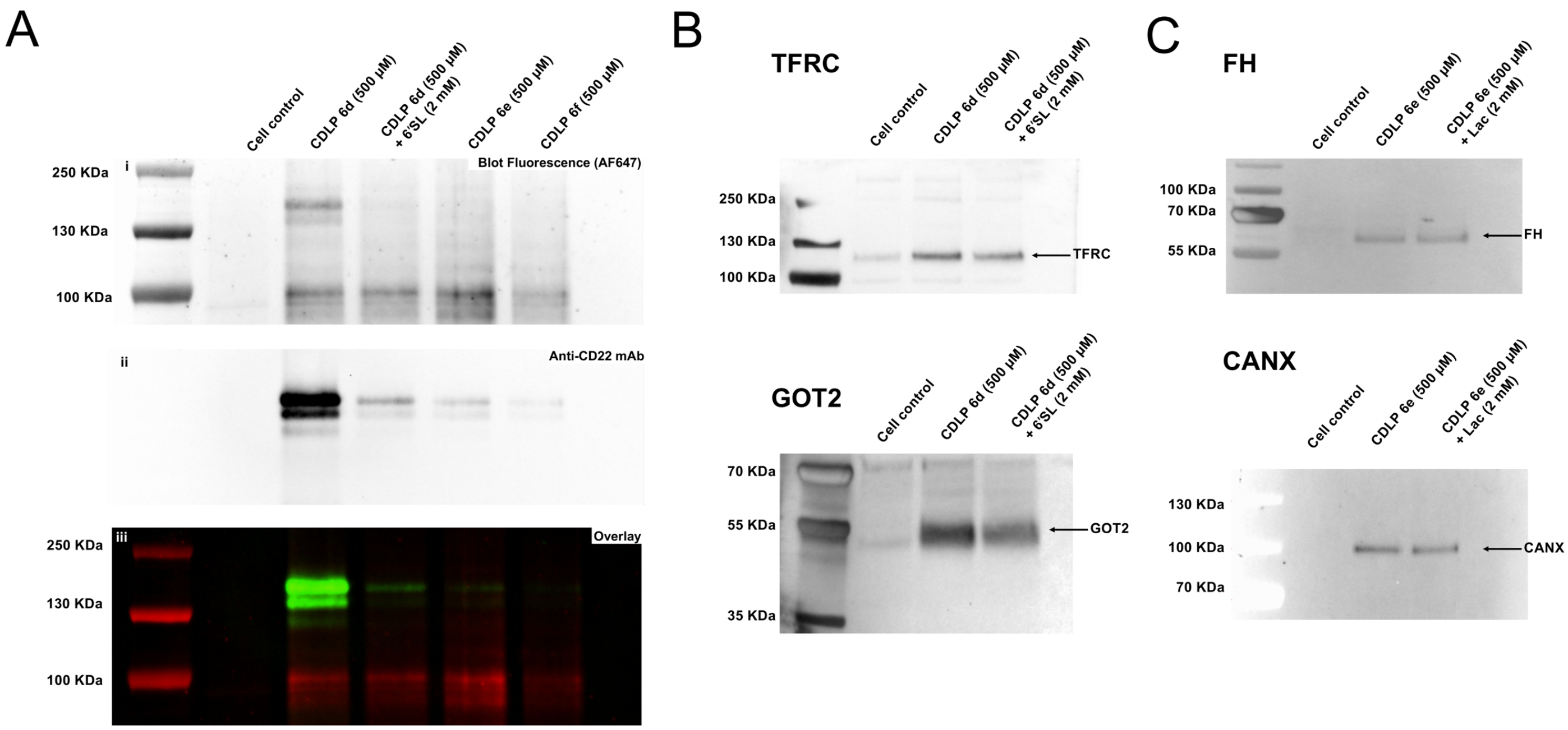

Figure S19. Immunoblot validation of representative CDLP-competition-sensitive candidate proteins. Daudi cells were treated with the indicated CDLP probes (500 µM) in the absence or presence of free glycan competitor (2 mM), functionalized with a desthiobiotin-affinity tag by SPAAC, enriched on streptavidin beads, separated by SDS-PAGE, and analyzed by streptavidin-AF647 detection of enriched desthiobiotin-tagged proteins together with target-specific immunoblotting. **A**) CD22 enrichment was strongest with 6′SL-CDLP **6d** (500 µM) and was reduced by free 6′SL competitor (2 mM); Lac-CDLP **6e** (500 µM) and 3′SL-CDLP **6f** (500 µM) showed weaker CD22 recovery. Streptavidin-AF647 detection, anti-CD22 immunoblotting, and overlay are shown. **B**) Representative 6′SL-CDLP **6d** candidates, TFRC and GOT2, were recovered from enriched fractions. **C**) Representative Lac-CDLP **6e** candidates, FH/fumarate hydratase and CANX/calnexin, were recovered from enriched fractions. Cell control indicates no-probe treatment; arrows mark the expected molecular-weight regions.

Full Gel and Blot Images: Figure S20–23

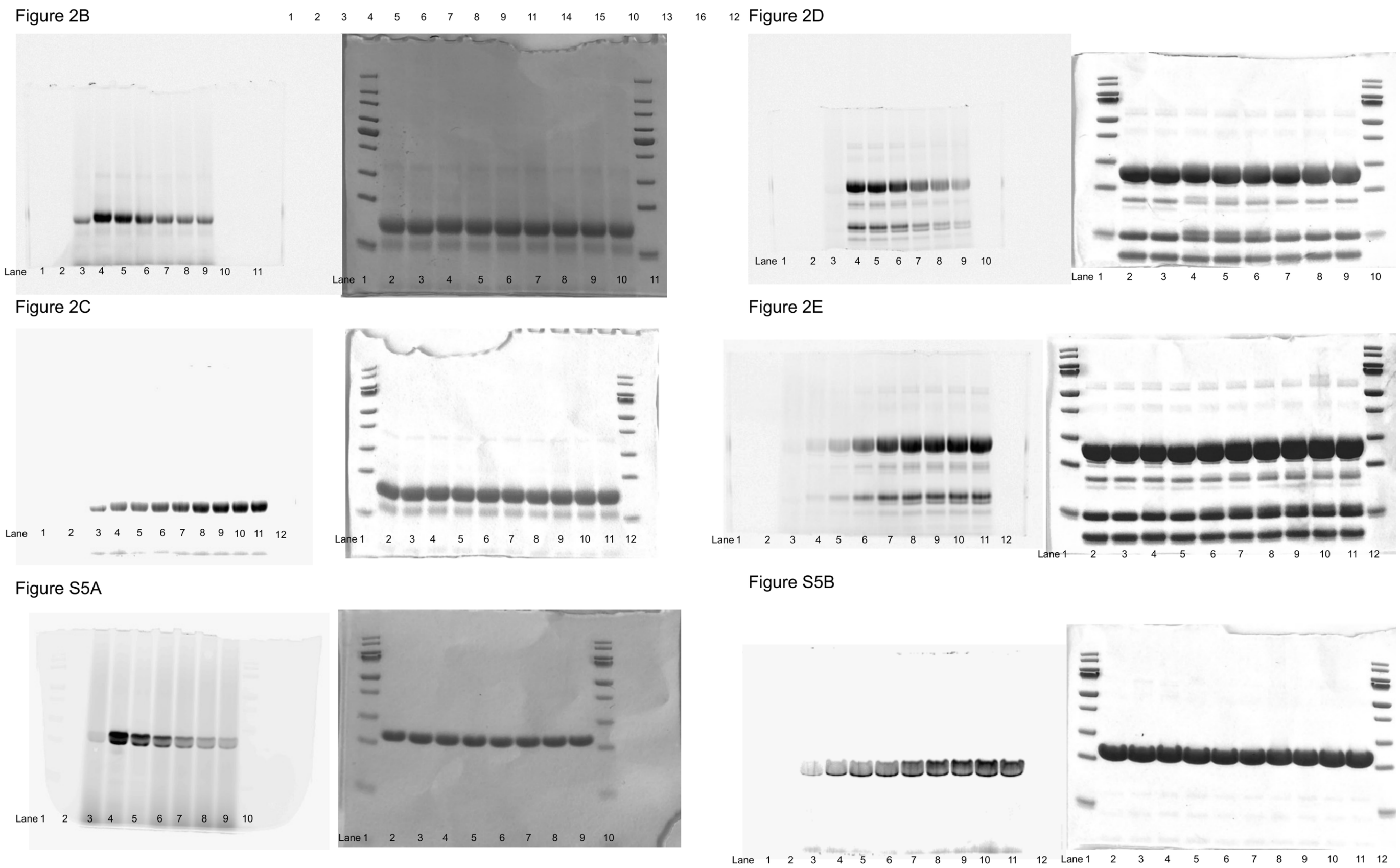

Figure S20. Full images of gels from Figures 2 and S5.

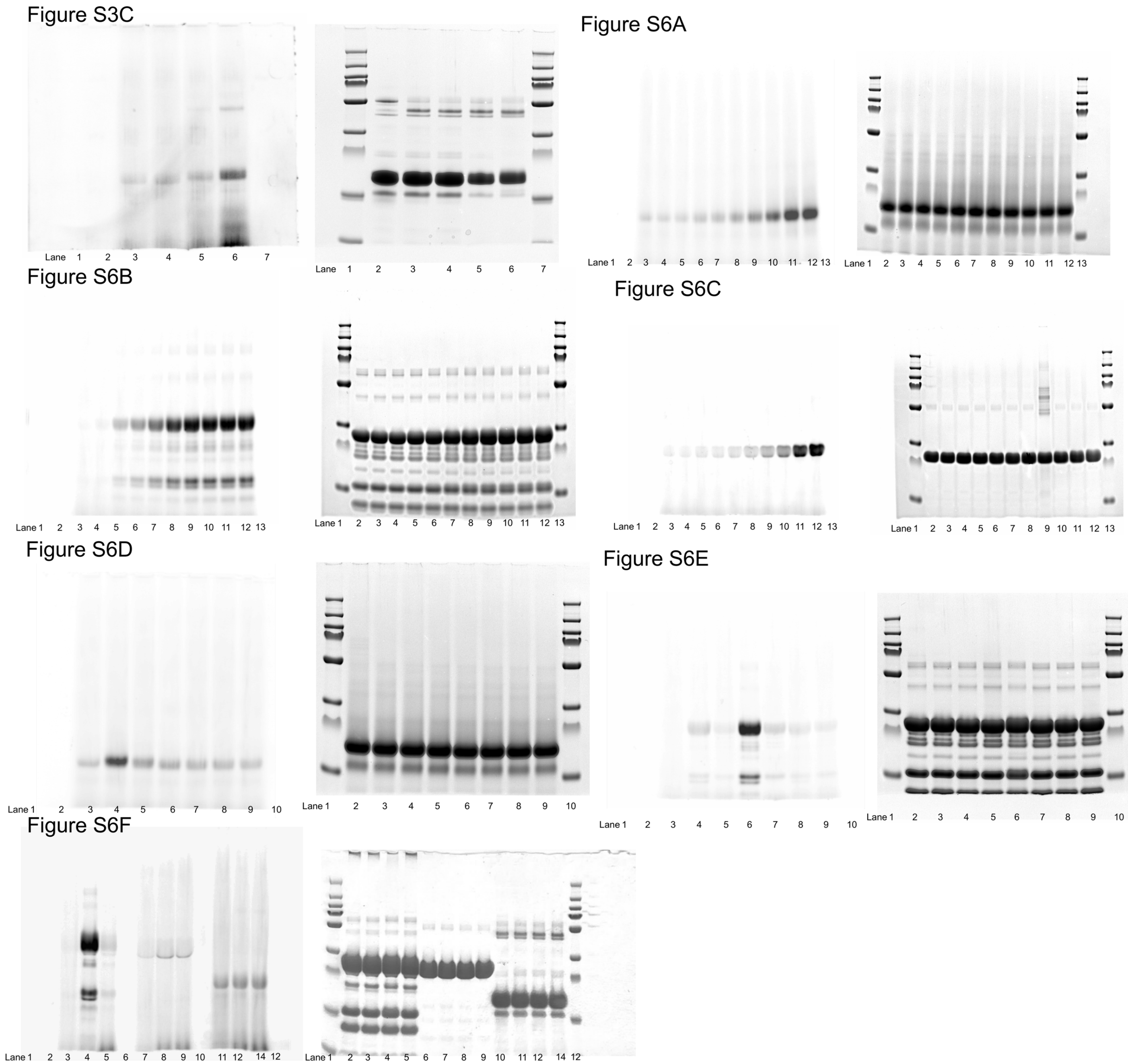

Figure S21. Full images of blots from Figures S3 and S6.

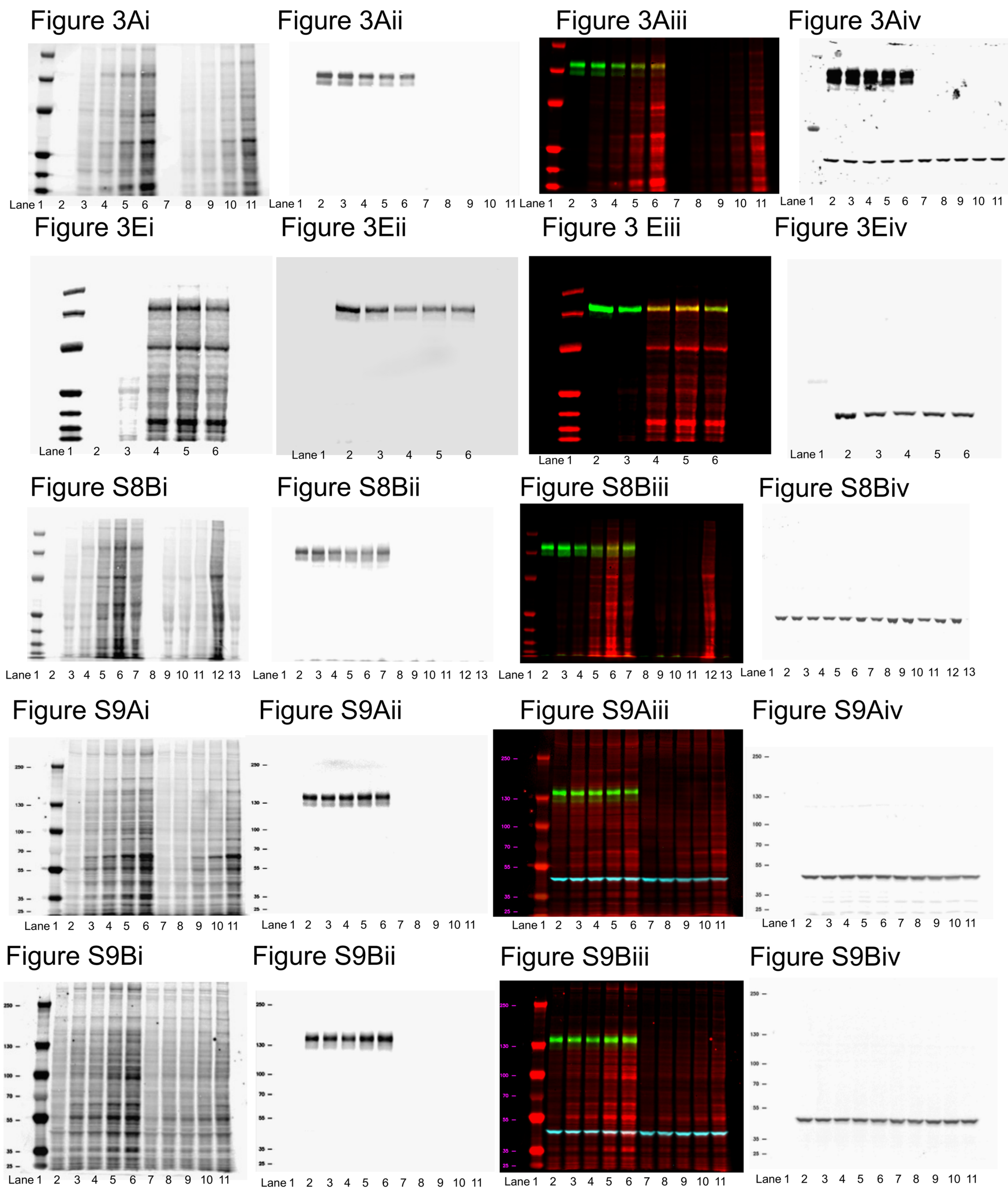

Figure S22. Full images of blots from Figures 3, S8, and S9.

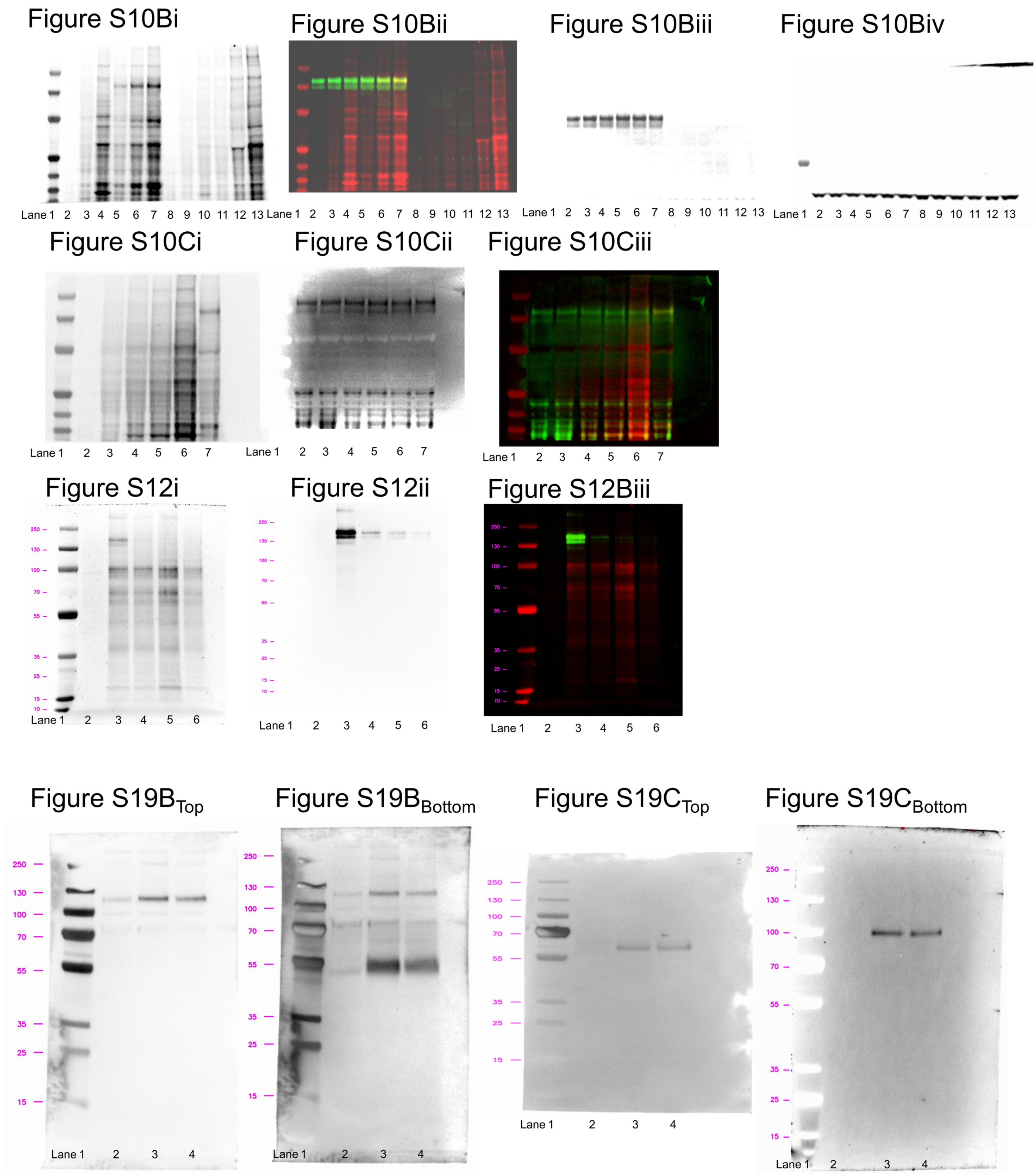

Figure S23. Full images of blots from Figures S10, S12, and S19.

Materials and Instrumentation

Chemistry Reagents and Solvents

Unless otherwise noted, reagents and solvents were used as received. General laboratory solvents were obtained from Fisher Scientific. Thin-layer chromatography (TLC) was performed on aluminum-backed silica gel plates (Sorbent Technologies), visualized under UV (254 nm) or with *p*-anisaldehyde staining, followed by gentle heating. Normal-phase column chromatography purification of the intermediates and final compounds was performed by a Teledyne ISCO CombiFlash Rf system using an appropriate silica gel pre-packed column, and a preparative reverse-phase Agilent Technologies 1200 HPLC system and a preparative Macherey-Nagel HPLC Column Isis 21 × 250 mm, 5 µm. Preparative runs were performed at a flow rate of 20.0 mL/min using a mixture of acetonitrile and Milli-Q water, both acidified with 0.1% TFA on a standard gradient (95% H_2O/5% ACN to 5% H_2O/95% ACN over 30 minutes). Activated carbon (Supelclean™ ENVI-Carb™ SPE Bulk Packing) was purchased from Millipore Sigma. The reported yields refer to chromatographically and spectroscopically isolated pure compounds unless otherwise noted.

NMR spectra were recorded on a Bruker Avance III 400 and an Avance III 500 with a cryoprobe. Chemical shifts were referenced to residual solvent peaks: CD_3CN (^1H, δ = 1.94 ppm; ^{13}C, δ = 118.3 ppm), CD_3OD (^1H, δ = 3.31 ppm; ^{13}C, δ = 49.00 ppm), D_2O (^1H, δ = 4.79 ppm), and $CDCl_3$ (^1H, δ = 7.26 ppm; ^{13}C, δ = 77.16 ppm). Chemical shifts (δ) are given in ppm. Spectra were analyzed using MestReNova 12.0.1. Signals are reported as follows: s = singlet, bs = broad singlet, d = doublet, t = triplet, quin. = quintet, dd = doublet of doublet, ddd = doublet of doublet of doublet, td = triplet of doublet, dt = doublet of triplet and m = multiplet. High-Resolution Mass Spectrometry (HRMS) analysis was performed using an electrospray ionization (ESI) source in positive- or negative-ion mode with a time-of-flight (TOF) analyzer on a Waters LCT Premier™ mass spectrometer, and the results are reported in m/z.

Cell Culture and Reagents

Daudi (CCL-213™, RRID: CVCL_0008) and Jurkat cells (TIB-152™, RRID:CVCL_0367) were purchased from the American Type Culture Collection. Both cell lines were cultured in RPMI-1640 medium supplemented with 10% fetal bovine serum (FBS), 100 U/mL penicillin, 100 µg/mL streptomycin, and 1.0 mM L-glutamine, and were maintained in a humidified incubator at 37 °C with 5% CO_2. Cell lines were maintained in T25 or T75 flasks and discarded after a maximum of 20 passages.

WGA and PNA were purchased from Vector Laboratories. ConA, Roswell Park Memorial Institute (RPMI) 1640 medium, penicillin-streptomycin, L-glutamine, fetal bovine serum (FBS), protease inhibitor, Pierce™ BCA protein assay kit, Pierce™ streptavidin magnetic beads, Click-iT® reaction cocktail, 3–8% NuPAGE™ Tris-acetate protein gels, 4–12% NuPAGE™ Bis-Tris protein gels, MES running buffer, Tris-buffered saline containing Tween-20 (TBST), β-actin polyclonal antibody (PA1-46296), Alexa Fluor 800-conjugated secondary antibody, Hanks' Balanced Salt Solution (HBSS), 7 kDa MWCO desalting column, TEAB buffer, TCEP, IAA, $CaCl_2$, and acetone were purchased from Thermo Fisher Scientific (Waltham, MA, US). 10 × PBS, bovine serum albumin (BSA), and Optima grade LC-MS water, acetonitrile, and formic acid were purchased from Fisher Scientific (Waltham, MA, US). RIPA buffer was purchased from Santa Cruz Biotechnology (Dallas, TX, US). DBCO-reagents were purchased from Broadpharm (San Diego, CA, US). AzDye 680 Azide was purchased from Vector Laboratories (Newark, CA, US). Anti-CD22 (E7L6Z) primary antibody was purchased from Cell Signaling Technology (Danvers, MA, US). 6′-sialyllactose and 3′-sialyllactose were purchased from Biosynth (Staad, Switzerland). Sequencing grade modified trypsin was purchased from Promega Corporation (Madison, WI, US).

Experimental Procedures

Synthetic Procedures

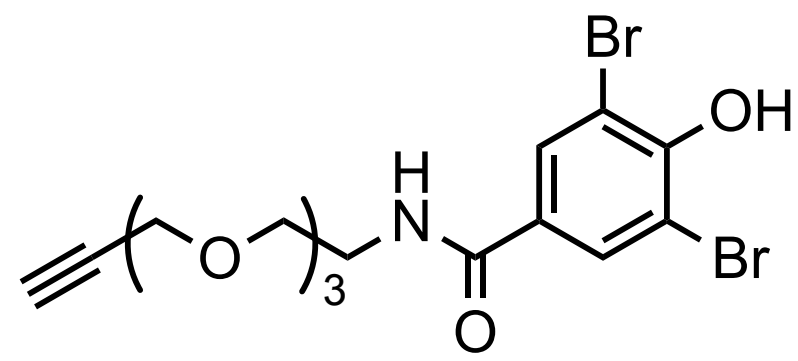

2

3,5-Dibromo-4-hydroxybenzoic acid (**1**, 350 mg, 1.2 mmol, 1 equiv.) was dissolved in 5 mL of dry DMF and cooled on ice. EDC·HCl (281 mg, 1.5 mmol, 1.2 equiv.) and HOBt (262 mg, 1.7 mmol, 1.4 equiv.) were added, followed by DIPEA (600 µL, 3.7 mmol, 3 equiv.) and Propargyl-PEG3-amine (250 mg, 1.3 mmol, 1.1 equiv.). The reaction was stirred overnight at room temperature and then concentrated under reduced pressure. The residue was redissolved in CH_2Cl_2 and poured into 1 N aq. HCl, and the aqueous phase was extracted with CH_2Cl_2 (3 × 20 mL). The combined organic layers were dried with Na_2SO_4, filtered, and concentrated under reduced pressure. The crude product **2** was used in subsequent steps without further purification; NMR data are reported for the crude material.

^{1}H NMR (400 MHz, $CDCl_3$) δ 7.97 (s, 2H), 4.17 (m, 2H), 3.76 – 3.62 (m, 12H), 2.48 (t, *J* = 2.3 Hz, 1H).
^{13}C NMR (101 MHz, $CDCl_3$) δ 164.6, 152.1, 131.3, 129.0, 110.00, 79.4, 74.7, 70.6, 70.3, 70.2, 69.7, 69.1, 40.0.

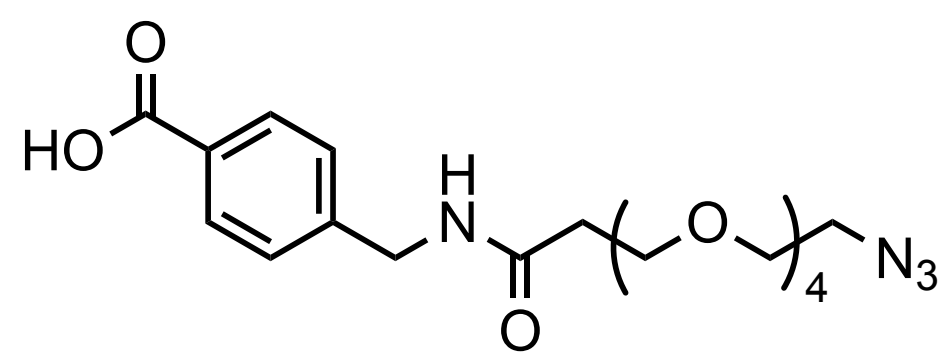

4

Azido-PEG4-NHS ester (200 mg, 0.515 mmol, 1 equiv.) was dissolved in 3 mL of dry DMF. 4-(Aminomethyl)benzoic acid (78 mg, 0.515 mmol, 1 equiv.) was added, followed by DIPEA (270 µL, 1.55 mmol, 3 equiv.), and the reaction was stirred overnight. The reaction mixture was then concentrated under reduced pressure, redissolved in CH_2Cl_2, and poured into 1 N aq. HCl, and the aqueous phase was extracted with CH_2Cl_2 (3 × 10 mL). The combined organic layers were washed with brine, dried with Na_2SO_4, filtered, and concentrated under reduced pressure. The crude product **4** was used in subsequent steps without further purification; NMR data are reported for the crude material.

^{1}H NMR (400 MHz, $CDCl_3$) δ 8.07 (d, *J* = 8.3 Hz, 2H), 7.68 (t, *J* = 5.9 Hz, 1H), 7.41 (d, *J* = 8.3 Hz, 2H), 4.59 (d, *J* = 5.9 Hz, 2H), 3.82 (t, *J* = 5.5 Hz, 2H), 3.72 – 3.55 (m, 17H), 3.39 (t, *J* = 5.0 Hz, 2H), 2.68 (t, *J* = 5.5 Hz, 2H).
^{13}C NMR (101 MHz, $CDCl_3$) δ 173.9, 170.8, 144.1, 130.6, 128.3, 127.5, 70.6, 70.5, 70.5, 70.4, 70.2, 70.1, 70.0, 66.9, 50.6, 43.3, 36.2.

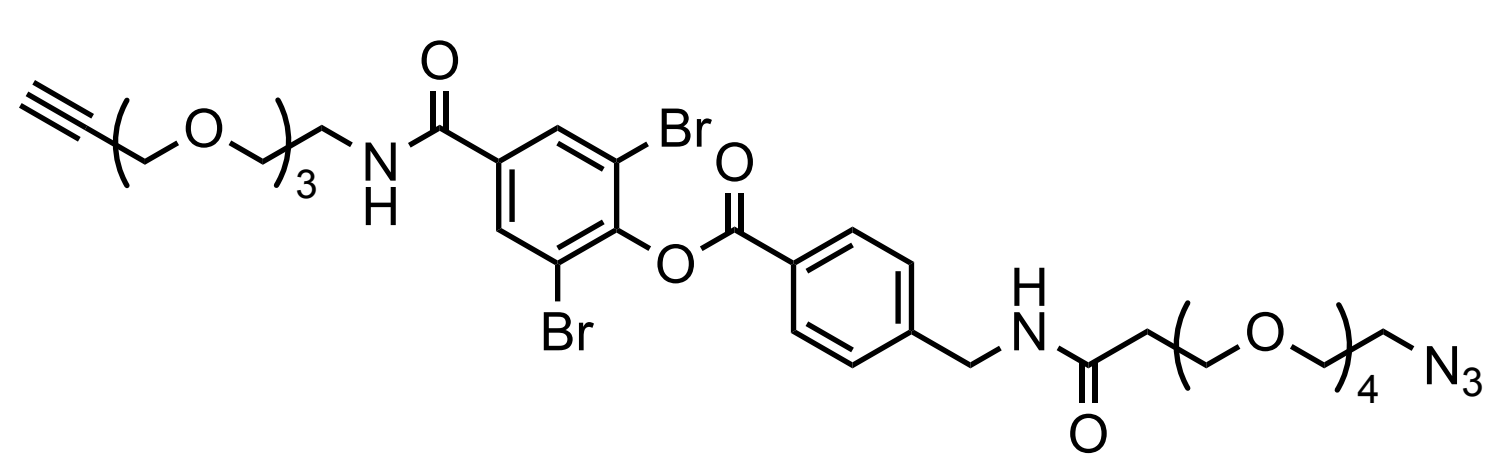

5

2 (400 mg, 0.86 mmol, 1 equiv.) and **4** (366 mg, 0.86 mmol, 1 equiv.) were dissolved in 3 mL of dry DMF. EDC·HCl (200 mg, 1.04 mmol, 1.2 equiv.) and DMAP (16 mg, 0.13 mmol, 0.15 equiv.) were added, and the reaction mixture was stirred overnight. The reaction mixture was directly purified by reverse-phase chromatography to afford **5** (651 mg, 85%) as a yellow oil.

^{1}H NMR (400 MHz, $CDCl_3$) δ 8.23 (d, *J* = 8.4 Hz, 2H), 8.10 (s, 2H), 7.50 (d, *J* = 8.4 Hz, 2H), 7.33 (t, *J* = 5.5 Hz, 1H), 7.06 (t, *J* = 5.1 Hz, 1H), 4.60 (d, *J* = 6.0 Hz, 2H), 4.17 (d, *J* = 2.4 Hz, 2H), 3.81 (t, *J* = 5.6 Hz, 2H), 3.76 – 3.57 (m, 27H), 3.39 (t, *J* = 5.1 Hz, 2H), 2.62 (t, *J* = 5.6 Hz, 2H), 2.44 (t, *J* = 2.3 Hz, 1H).
^{13}C NMR (101 MHz, $CDCl_3$) δ 172.3, 164.3, 162.6, 148.8, 134.9, 131.4, 130.9, 127.6, 126.9, 118.2, 79.4, 74.8, 70.6, 70.6, 70.5, 70.4, 70.4, 70.3, 70.3, 70.2, 70.0, 69.6, 69.1, 67.2, 58.4, 50.7, 43.0, 40.2, 36.8.
HRMS (ESI) calcd for $C_{35}H_{45}Br_2N_5O_{11}Na$ $[M+Na]^+$: 892.1380; found 892.1423.

General Procedure for Shoda Reagent Activation

Unprotected oligosaccharide (lactose or sialyllactose; 0.16 mmol, 1 equiv.) was dissolved in D_2O to a concentration of 250 mM (640 µL), NaN_3 (103 mg, 1.6 mmol, 10 equiv.), and DIPEA (330 µL, 1.9 mmol, 12 equiv.) were added, and the solution was cooled on ice. DMC (80 mg, 0.47 mmol, 3 equiv.) was added, and the solution was stirred on ice for 2 hours. After completion, the solution was evaporated to dryness, redissolved in DMF, and subsequently filtered. The filtrate was then concentrated under reduced pressure. The resulting residue was redissolved in water, washed with CH_2Cl_2 (3×), and evaporated to dryness. The crude product was purified using an activated carbon column. Spectral data match previously reported information.[70-71]

Activated Carbon Column Purification

The column was washed with 2 column volumes of CH_3CN, followed by 2 column volumes of H_2O. The sample was dissolved in minimal H_2O and loaded onto the column. Sample loading did not exceed 5 wt% relative to the activated carbon bed. The column was then washed with 2 column volumes of H_2O to remove salts and aqueous impurities. The product was then eluted by washing the column with 40:60 CH_3CN/H_2O (v/v; acidified with 0.1% TFA).

Synthesis of Monomeric Carbohydrate Ligands

The following carbohydrate monomers with azidopropyl linkers were synthesized as previously reported, and the spectral data match previously reported information:[72-73]

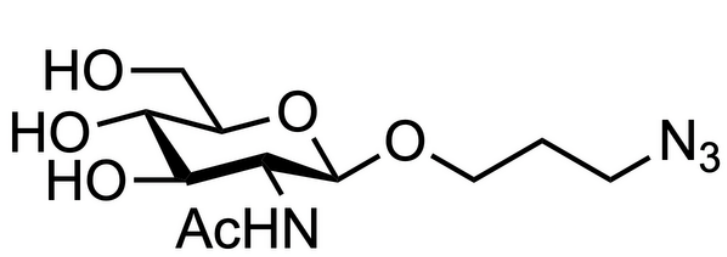

GlcNAc

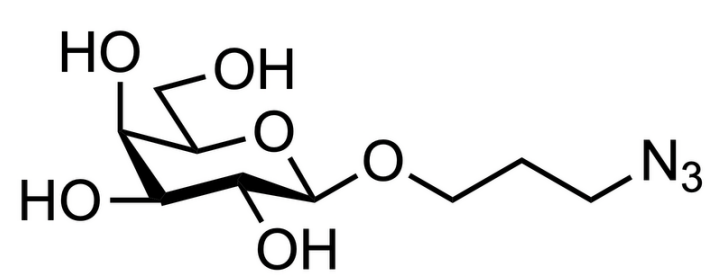

Gal

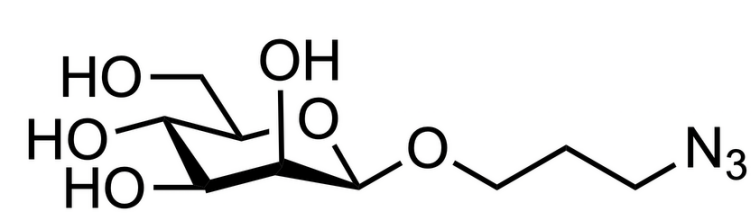

Man

General Click Procedure

5 (10 mg, 0.0115 mmol, 1 equiv.) and **Sugar-N_3** (0.0575 mmol, 5 equiv.) were dissolved in 1 mL of ACN:Water (1:1) and sparged with argon for 2 minutes. Sodium ascorbate (23 mg, 0.115 mmol, 10 equiv.) was added, and the reaction mixture was degassed for an additional minute. THPTA (5 mg, 0.0115 mmol, 1 equiv.) and $CuSO_4$ (2.4 mg, 0.0115 mmol, 1 equiv.) were added, and the reaction mixture was stirred at room temperature overnight under an inert argon atmosphere.[74-75] The reaction mixture was directly purified via HPLC.

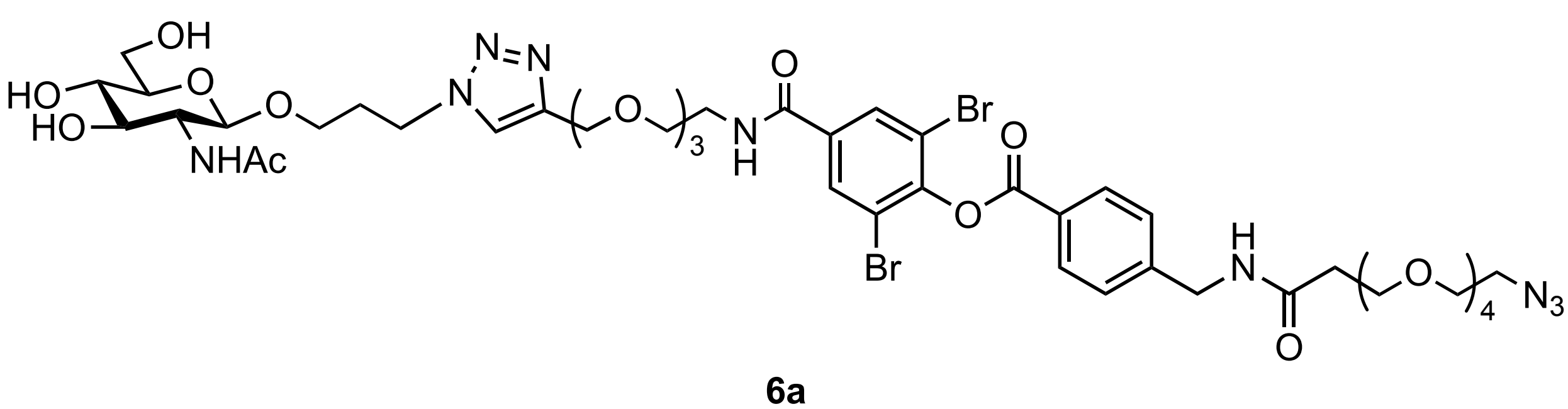

6a

(37%) **^{1}H NMR (400 MHz, CD_3CN)** δ 8.21 (d, *J* = 8.3 Hz, 2H), 8.15 (s, 2H), 7.78 (s, 1H), 7.54 (m, 3H), 7.29 (t, *J* = 6.0 Hz, 1H), 6.75 (d, *J* = 8.2 Hz, 1H), 4.58 (s, 2H), 4.52 (d, *J* = 6.1 Hz, 2H), 4.44 (m, 2H), 4.35 (d, *J* = 8.5 Hz, 1H), 3.87 – 3.72 (m, 5H), 3.68 – 3.50 (m, 30H), 3.44 – 3.36 (m, 4H), 3.34 – 3.22 (m, 2H), 2.50 (t, *J* = 5.9 Hz, 2H), 1.95 (s, 3H).
^{13}C NMR (126 MHz, CD_3CN) δ 172.0, 171.7, 164.4, 163.3, 148.9, 147.8, 135.9, 132.1, 131.0, 128.3, 126.8, 118.2, 101.6, 76.7, 75.9, 71.7, 70.7, 70.7, 70.6, 70.5, 70.0, 70.0, 69.6, 67.5, 66.0, 64.5, 62.3, 56.8, 51.0, 47.2, 42.9, 40.5, 37.0, 30.6, 23.0.
HRMS (ESI) calcd for $C_{46}H_{65}Br_2N_9O_{17}Na$ $[M+Na]^+$: 1196.2763; found 1196.2717.

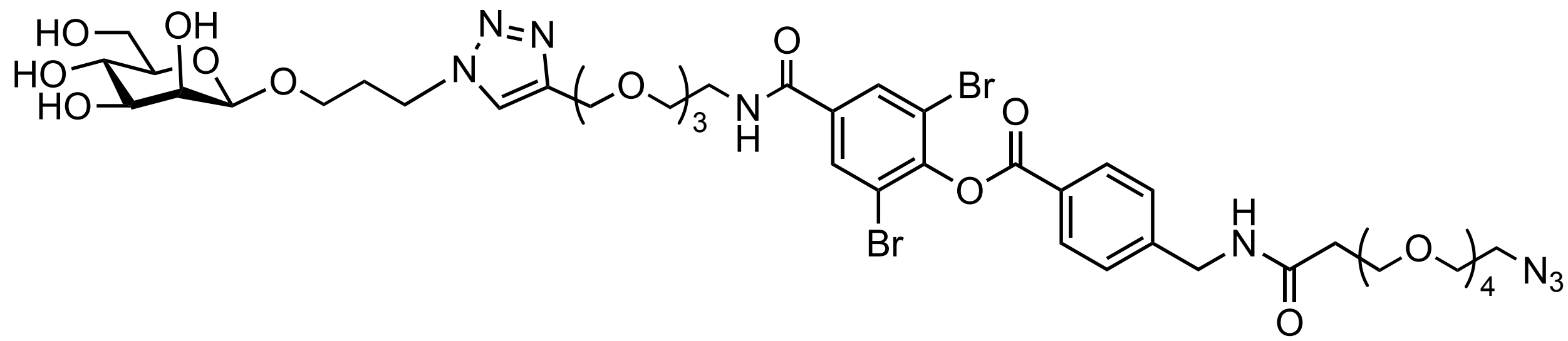

6b

(29%) **^{1}H NMR (400 MHz, CD_3CN)** δ 8.21 (d, *J* = 8.3 Hz, 2H), 8.15 (s, 2H), 7.75 (s, 1H), 7.56 (m, 3H), 7.32 (t, *J* = 6.0 Hz, 1H), 4.67 (d, *J* = 1.3 Hz, 1H), 4.58 (s, 2H), 4.52 (d, *J* = 6.1 Hz, 2H), 4.46 (t, *J* = 6.8 Hz, 2H), 3.75 (t, *J* = 5.7 Hz, 2H), 3.73 – 3.52 (m, 34H), 3.40 (m, 4H), 2.51 (t, *J* = 5.7 Hz, 2H), 2.15 (p, *J* = 6.4 Hz, 2H).
^{13}C NMR (126 MHz, CD_3CN) δ 172.2, 164.5, 163.3, 148.9, 147.7, 144.9, 135.8, 132.2, 131.0, 128.3, 126.9, 124.0, 118.2, 101.3, 100.6, 73.4, 71.9, 71.1, 70.6, 70.6, 70.6, 70.5, 70.5, 70.5, 70.4, 70.0, 69.9, 69.7, 68.3, 67.5, 64.6, 64.5, 62.3, 51.0, 48.0, 42.9, 40.4, 36.9, 30.3.
HRMS (ESI) calcd for $C_{44}H_{62}Br_2N_8O_{17}Na$ $[M+Na]^+$: 1155.2497; found 1155.2482.

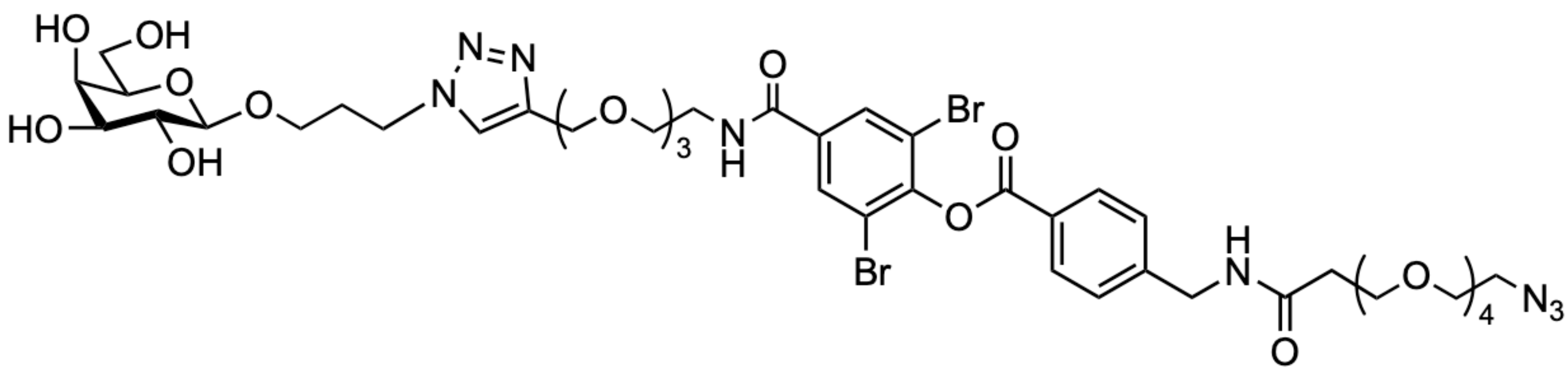

6c

(26%) **^{1}H NMR (400 MHz, CD_3CN)** δ 8.20 (d, *J* = 8.3 Hz, 2H), 8.15 (s, 2H), 7.83 (s, 1H), 7.56 (d, *J* = 8.3 Hz, 2H), 7.49 (t, *J* = 5.4 Hz, 1H), 7.27 (t, *J* = 6.1 Hz, 1H), 4.58 (s, 2H), 4.50 (m, 4H), 4.16 (d, *J* = 7.2 Hz, 1H), 3.85 – 3.35 (m, 43H), 2.50 (t, *J* = 5.8 Hz, 2H), 2.14 (p, *J* = 6.6 Hz, 2H).
^{13}C NMR (126 MHz, CD_3CN) δ 172.0, 164.4, 163.3, 148.9, 147.8, 145.0, 135.9, 132.1, 131.0, 128.3, 126.8, 124.4, 118.2, 103.9, 75.6, 74.0, 71.9, 70.7, 70.7, 70.5, 70.5, 70.0, 69.9, 69.6, 69.6, 67.5, 66.1, 64.5, 62.1, 51.0, 47.4, 42.9, 40.5, 37.0, 30.8.
HRMS (ESI) calcd for $C_{44}H_{62}Br_2N_8O_{17}Na$ $[M+Na]^+$: 1155.2497; found 1155.2499.

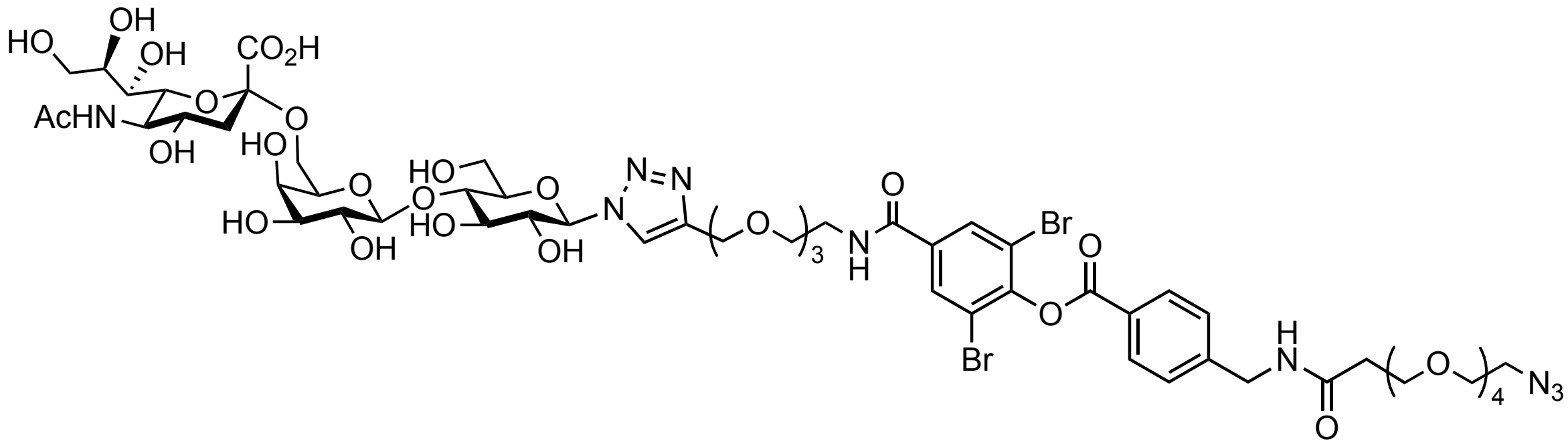

6d

(30%) **^{1}H NMR (400 MHz, D_2O)** δ 8.09 (s, 1H), 7.97 (d, *J* = 8.0 Hz, 2H), 7.89 (s, 2H), 7.29 (d, *J* = 8.0 Hz, 2H), 5.64 (d, *J* = 9.3 Hz, 1H), 5.23 (t, *J* = 4.8 Hz, 2H), 4.50 (s, 2H), 4.35 (m, 3H), 3.92 (m, 2H), 3.85 – 3.36 (m, 51H), 2.61 (dd, *J* = 12.5, 4.6 Hz, 1H), 2.46 (s, 2H), 1.91 (s, 3H), 1.73 (t, *J* = 12.2 Hz, 1H).
^{13}C NMR (126 MHz, D_2O) δ 174.8, 173.8, 172.2, 165.4, 163.6, 148.2, 145.9, 144.2, 134.1, 131.5, 130.7, 127.6, 125.9, 124.0, 117.6, 103.3, 99.5, 87.2, 78.7, 77.5, 74.7, 73.6, 72.7, 72.4, 71.9, 71.3, 70.7, 69.7, 69.6, 69.6, 69.3, 69.1, 68.8, 68.5, 68.4, 67.9, 66.9, 63.5, 63.1, 62.8, 59.9, 51.7, 50.1, 42.6, 39.9, 39.5, 36.2, 22.1.
HRMS (ESI) calcd for $C_{58}H_{82}Br_2N_9O_{29}$ $[M-H]^-$: 1526.3585; found 1526.3628.

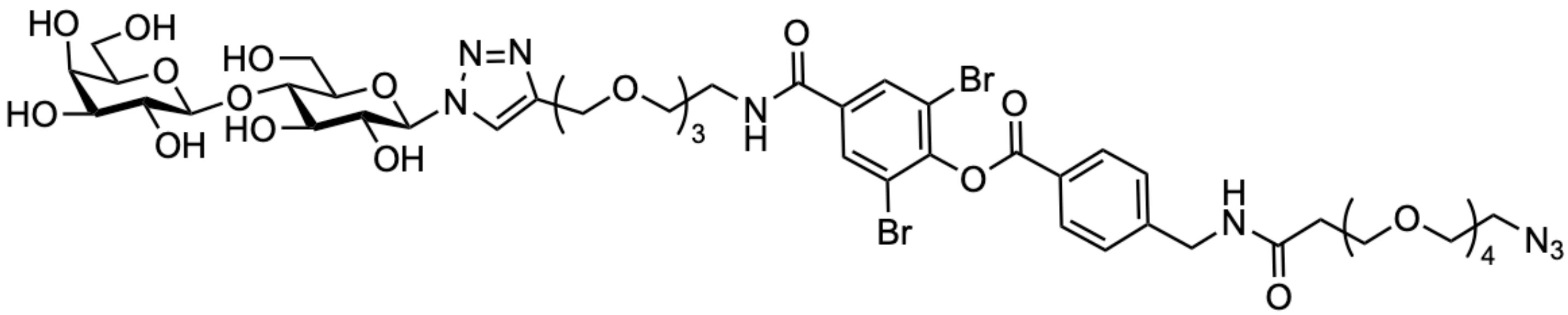

6e

(25%) **^{1}H NMR (400 MHz, D_2O)** δ 8.10 (s, 1H), 7.94 (d, *J* = 7.2 Hz, 2H), 7.89 (s, 2H), 7.26 (d, *J* = 7.6 Hz, 2H), 5.65 (d, *J* = 9.1 Hz, 1H), 4.50 (s, 2H), 4.42 (d, *J* = 7.7 Hz, 1H), 4.30 (br s, 2H), 3.95 (t, *J* = 8.9 Hz, 1H), 3.89 – 3.32 (m, 38H), 3.19 (t, *J* = 4.7 Hz, 2H), 2.44 (br s, 2H).
^{13}C NMR (126 MHz, D_2O) δ 173.6, 165.1, 163.3, 148.2, 144.2, 134.1, 131.5, 130.6, 127.6, 125.9, 124.0, 117.6, 102.9, 87.3, 77.7, 77.3, 75.4, 74.6, 72.6, 72.0, 70.9, 69.7, 69.7, 69.6, 69.3, 69.1, 68.8, 68.5, 66.9, 63.1, 61.0, 59.7, 50.1, 39.8, 36.2.
HRMS (ESI) calcd for $C_{47}H_{66}Br_2N_8O_{21}Na$ $[M+Na]^+$: 1259.2607; found 1259.2579.

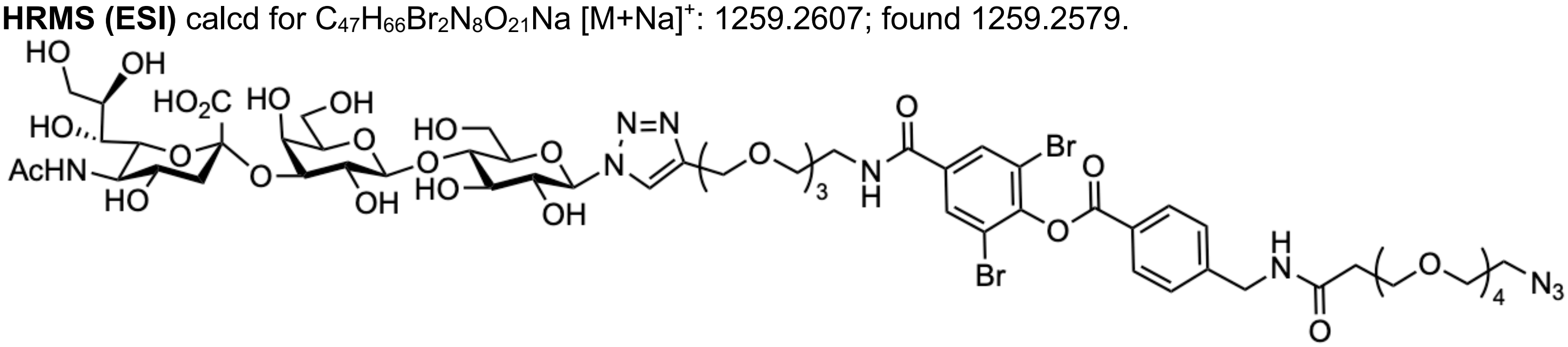

6f

(34%) **^{1}H NMR (400 MHz, D_2O)** δ 8.09 (s, 1H), 7.92 (d, *J* = 7.8 Hz, 2H), 7.87 (s, 2H), 7.25 (d, *J* = 7.8 Hz, 2H), 5.62 (d, *J* = 9.1 Hz, 1H), 4.49 (m, 3H), 4.29 (br s, 2H), 4.08 (dd, *J* = 9.9, 2.8 Hz, 1H), 3.98 – 3.87 (m, 3H), 3.84 – 3.32 (m, 52H), 3.19 (t, *J* = 4.8 Hz, 2H), 2.67 (dd, *J* = 12.4, 4.1 Hz, 1H), 2.43 (br s, 2H), 1.94 (s, 3H), 1.80 (t, *J* = 12.2 Hz, 1H).
^{13}C NMR (126 MHz, D_2O) δ 175.0, 173.7, 172.3, 165.2, 163.4, 148.2, 145.9, 144.1, 134.1, 131.5, 130.6, 127.6, 125.9, 124.1, 117.6, 102.6, 99.0, 87.3, 77.7, 77.2, 75.5, 75.1, 74.6, 73.1, 72.0, 71.2, 69.7, 69.6, 69.6, 69.4, 69.3, 69.1, 68.8, 68.1, 67.8, 67.6, 66.9, 63.1, 62.8, 60.9, 59.7, 51.7, 50.1, 42.6, 39.9, 39.0, 36.3, 22.1.
HRMS (ESI) calcd for $C_{58}H_{82}Br_2N_9O_{29}$ $[M-H]^-$:1526.3585; found 1526.3625.

Synthesis of 6′-Sialyllactose Alkyne CDLP (S5)

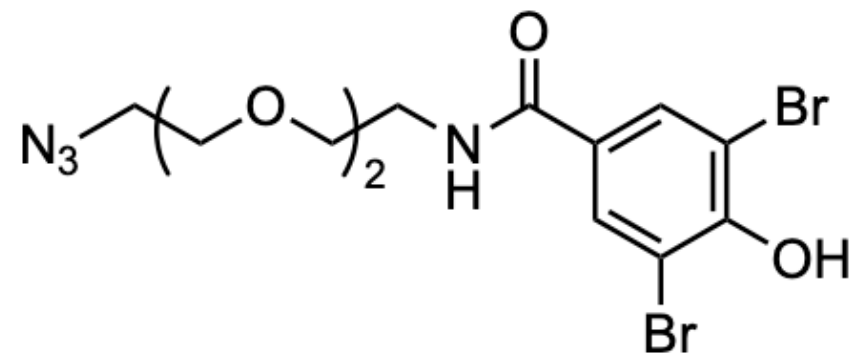

S1

A round-bottom flask was charged with 3,5-dibromo-4-hydroxybenzoic acid (2.5 g, 8.44 mmol, 1 equiv.) and dissolved in DMF (5 mL). After cooling the solution on ice, EDC·HCl (1.94 g, 10.13 mmol, 1.2 equiv.) and HOBt (1.81 g, 11.82 mmol, 1.4 equiv.) were added, followed by DIPEA (4.42 mL, 25.37 mmol, 3 equiv.) and Azido-PEG2-Amine (1.6 g, 9.3 mmol, 1.1 equiv.). The reaction mixture was stirred overnight, then concentrated under reduced pressure. The crude reaction mixture was resuspended in CH_2Cl_2, then poured into 1 N aq. HCl, and the aqueous phase was extracted with CH_2Cl_2 (3 × 10 mL). The combined organic layers were dried over Na_2SO_4, then filtered and concentrated under reduced pressure. The crude reaction mixture was purified using silica gel chromatography (5% CH_3OH/95% CH_2Cl_2), yielding **S1** (3.7 g, 96%).
^{1}H NMR (400 MHz, CDCl3) δ 7.94 (s, 2H), 3.75 – 3.63 (m, 11H), 3.41 (t, *J* = 5.0 Hz, 2H).
^{13}C NMR (101 MHz, CDCl3) δ 164.5, 152.0, 131.2, 129.1, 109.9, 89.0, 70.6, 70.3, 70.1, 69.7, 50.6, 40.0.
HRMS (ESI) calcd for $C_{13}H_{16}Br_2N_4O_4Na$ $[M+Na]^+$: 472.9436; found 472.9428.

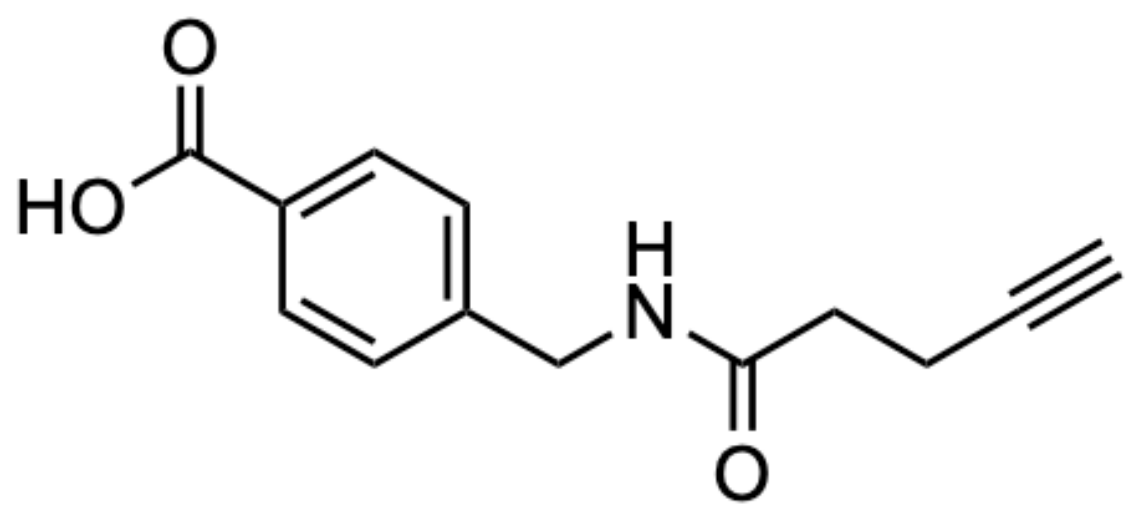

S2

4-Aminomethylbenzoic acid (229 mg, 1.5 mmol, 1 equiv.) and Na_2CO_3 (480 mg, 4.5 mmol, 3 equiv.) were dissolved in H_2O:THF (3:1, 20 mL). Pent-4-ynoic acid succinimidyl ester (295 mg, 1.5 mmol, 1 equiv.) was added in one portion and stirred for 2 hours at room temperature. Ethyl acetate (20 mL) and saturated aq. $NaHCO_3$ (2 mL) were added, and the aqueous phase was separated before being washed twice with ethyl acetate. The aqueous phase was acidified with 1 N aq. HCl until precipitation occurred, then extracted with ethyl acetate. The organic phase was washed with brine, dried over Na2SO4, filtered, and evaporated to yield **S2**. The crude product was used in subsequent steps without further purification (300 mg, 87%).
^{1}H NMR (400 MHz, CD_3OD) δ 7.97 (d, J = 8.5 Hz, 2H), 7.41 (d, J = 8.6 Hz, 2H), 4.45 (s, 2H), 2.49 (m, 4H), 2.29 (t, J = 2.5 Hz, 1H)**.**
^{13}C NMR (101 MHz, CD_3OD) δ 174.1, 169.7, 145.4, 130.9, 130.8, 128.4, 83.5, 70.4, 43.7, 36.0, 15.8.
HRMS (ESI) calcd for $C_{13}H_{13}NO_3$ $[M+H]^+$: 232.0968; found 232.0975.

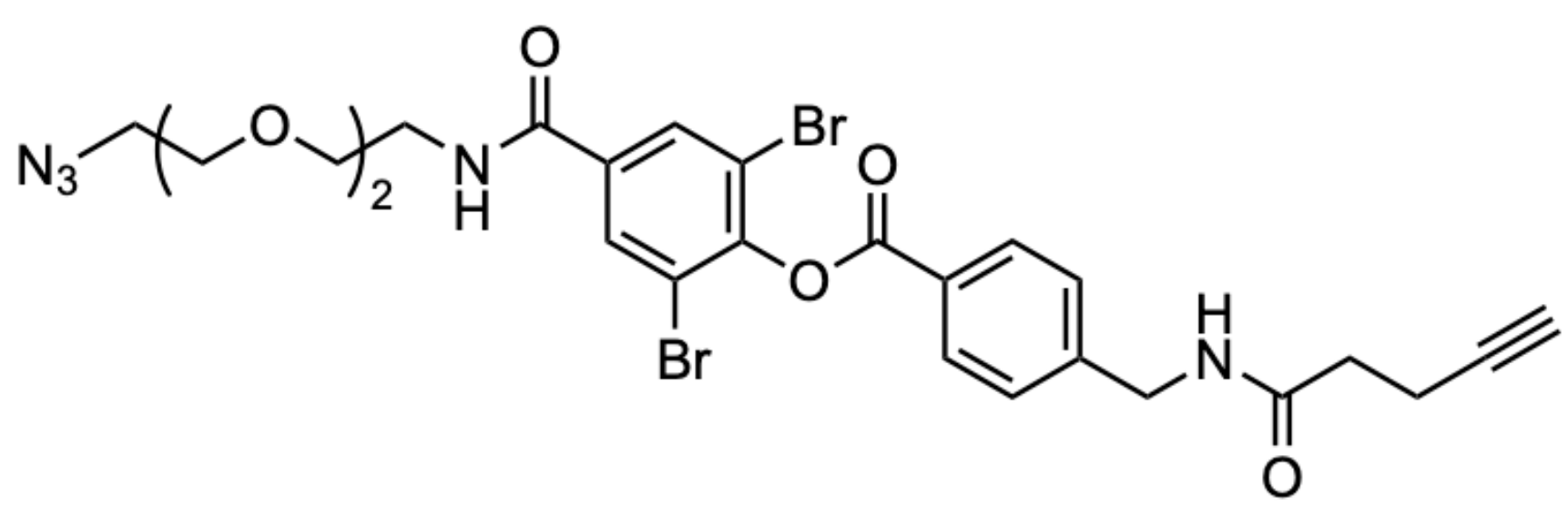

S3

In a 100 mL round-bottom flask, **S1** (397.5 mg, 0.87 mmol, 1 equiv.) was dissolved in DMF (40 mL) and cooled on ice. Then, **S2** (223.7 mg, 0.96 mmol, 1.1 equiv.) was added, followed by EDC·HCl (252.8 mg, 1.31 mmol, 1.5 equiv.) and DMAP (5 mg, 0.04 mmol, 0.05 equiv.). The reaction was stirred overnight at room temperature, then concentrated under reduced pressure. The crude substance was dissolved in CH_2Cl_2 and then transferred to 1 N aq. HCl. The organic layer was then separated, and the aqueous layer was extracted with CH_2Cl_2 (3 × 10 mL).

The combined organic extracts were dried over Na_2SO_4, filtered, and concentrated under reduced pressure, then purified by silica gel chromatography (1:1 Toluene:Acetone), yielding **S3** (280.8 mg, 48%).
^{1}H NMR (400 MHz, CDCl$_3$) δ 8.21 (d, *J* = 8.3 Hz, 2H), 8.02 (s, 2H), 7.47 (d, *J* = 8.3 Hz, 2H), 6.81 (m, 1H), 6.24 (t, *J* = 5.3 Hz, 1H), 4.58 (d, *J* = 6.1 Hz, 2H), 3.69 (m, 10H), 3.39 (t, *J* = 5.1 Hz, 2H), 2.59 (td, *J* = 6.3, 2.1 Hz, 2H), 2.49 (t, *J* = 6.9 Hz, 2H), 2.03 (t, *J* = 2.5 Hz, 1H).
^{13}C NMR (101 MHz, CDCl$_3$) δ 171.3, 164.4, 162.6, 148.9, 145.2, 135.0, 131.4, 131.2, 128.0, 83.0, 69.7.
HRMS (ESI) calcd for $C_{26}H_{27}Br_2N_5O_6$ $[M+Na]^+$: 686.0226; found 686.0245.

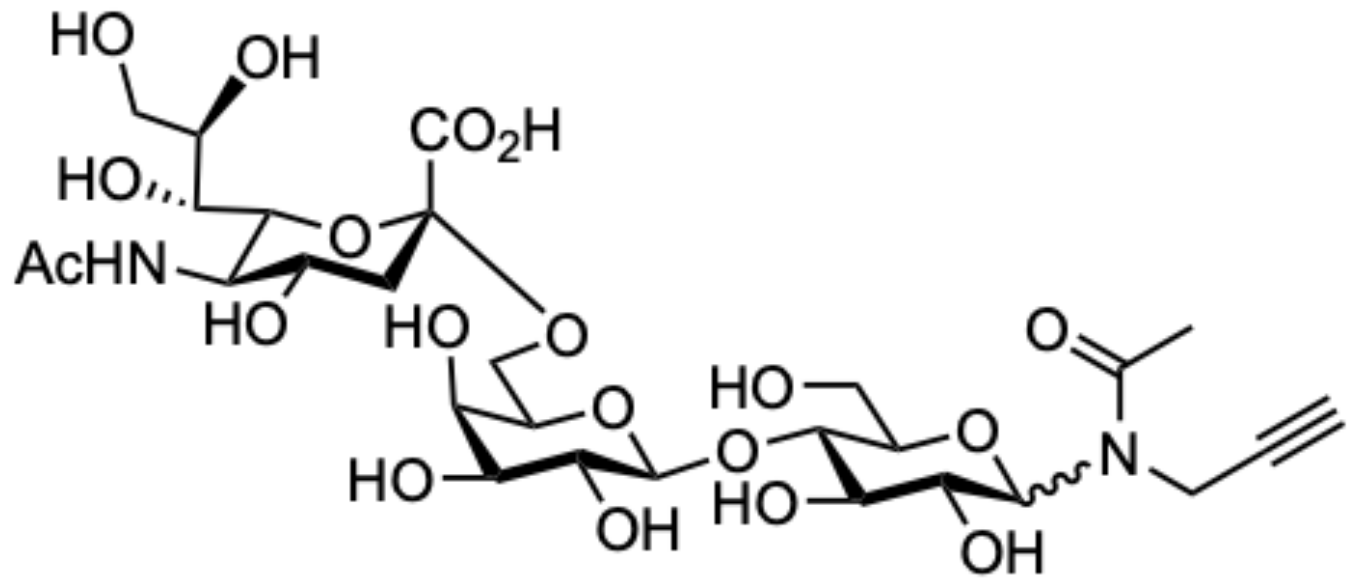

S4

6′-Sialyllactose (50 mg, 0.08 mmol, 1 equiv.) was dissolved in neat propargyl amine (150 µL, 2.34 mmol, 30 equiv.) and stirred for 48 h, monitoring for completion via TLC. Upon completion, the reaction mixture was concentrated under reduced pressure. The crude product was resuspended in dry CH_3OH (2 mL). Acetic anhydride (200 µL, 25 equiv.) was added, and the reaction mixture was stirred for 48 h. The reaction mixture was concentrated under reduced pressure, and the crude product was purified via flash chromatography (Isopropanol/H_2O/NH_4OH 7:2:1, v/v/v). Fractions containing the pure product were combined, acidified with 1 N aq. HCl to pH ≈ 4, and concentrated under reduced pressure. The product was further purified by passage through an activated carbon column to afford 6′SL-alkyne (**S4**) as a yellow solid (40 mg, 71%, α:β = 1:2). NMR integrations reflect an α/β anomeric mixture. Spectral data are consistent with previously reported information.[76]
^{1}H NMR (400 MHz, D$_2$O) δ 5.48 (d, *J* = 8.7 Hz, 1H, α isomer), 5.02 (d, *J* = 8.7 Hz, 1H, β isomer), 4.36 (d, *J* = 7.9 Hz, 1H), 4.25 – 4.05 (m, 2H), 4.00 – 3.90 (m, 9H), 3.99 – 3.91 (m, 2H), 3.88 – 3.52 (m, 21H), 3.50 – 3.44 (m, 3H), 2.68 (t, *J* = 2.3 Hz, 1H, α isomer), 2.62 (dd, *J* = 12.6, 4.5 Hz, 1H), 2.51 (t, *J* = 2.3 Hz, 1H, β isomer), 2.25 (s, 3H), 2.17 (s, 1H, α isomer), 1.95 (s, 2H, β isomer), 1.77 (t, *J* = 12.3 Hz, 1H).
^{13}C NMR (101 MHz, D$_2$O) δ 174.9, 174.7, 173.5, 103.2, 100.3, 86.3, 79.9, 79.0, 76.7, 76.6, 75.2, 73.7, 72.5, 72.4, 71.8, 71.7, 70.8, 69.9, 68.5, 68.4, 68.3, 63.6, 62.7, 60.1, 51.8, 48.9, 40.1, 32.8, 30.1, 22.1, 21.6, 21.2.

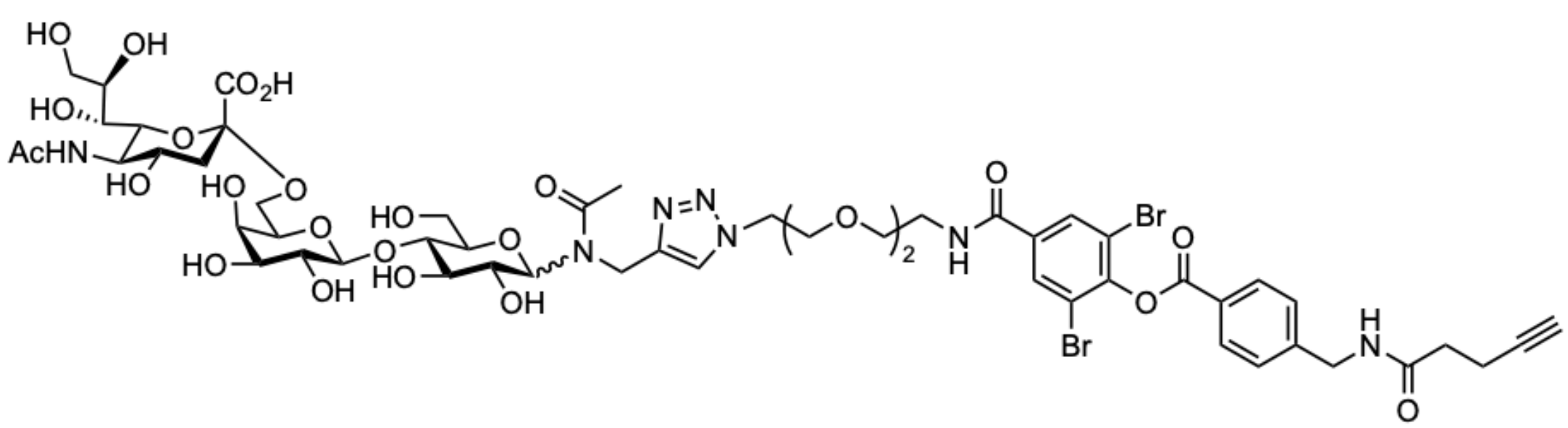

S5

S3 (10 mg, 0.015 mmol, 1 equiv.) and S4 (54 mg, 0.075 mmol, 5 equiv.) were dissolved in 1 mL of CH_3CN:Water (1:1) and degassed for 2 minutes. Sodium Ascorbate (30 mg, 0.15 mmol, 10 equiv.) was added, and the reaction mixture was degassed for an additional minute. THPTA (6.5 mg, 0.015 mmol, 1 equiv.) and $CuSO_4$ (2.4 mg, 0.015 mmol, 1 equiv.) were added, and the reaction mixture was stirred at room temperature overnight. The reaction mixture was purified directly by HPLC (6 mg, 29%).
^{1}H NMR (400 MHz, DMSO-d6) δ 8.83 (t, J = 5.3 Hz, 1H), 8.59 (t, J = 6.0 Hz, 1H), 8.24 (s, 2H), 8.17 (d, J = 8.3 Hz, 2H), 8.06 (s, 1H), 7.97 (m, 1H), 7.89 (s, 1H), 7.56 (d, J = 8.3 Hz, 2H), 5.49 (d, J = 9.4 Hz, 1H), 4.90 (d, J = 8.6 Hz, 0.7H), 4.59 – 4.40 (m, 6H), 4.26 (d, J = 6.1 Hz, 1H), 3.95 – 3.23 (m, 42H), 2.87 (t, J = 2.4 Hz, 1H), 2.47 – 2.35 (m, 4H), 2.11 (s, 2H), 1.98 (s, 1H), 1.88 (s, 3H), 1.64 – 1.53 (m, 1H).
^{13}C NMR (126 MHz, DMSO-d6) δ 172.1, 172.1, 172.0, 171.3, 171.1, 170.8, 163.5, 162.8, 148.2, 147.7, 145.6, 145.5, 135.4, 131.9, 130.8, 128.3, 126.1, 124.5, 124.3, 117.9, 104.1, 87.1, 84.2, 82.0, 80.5, 80.4, 77.5, 77.1,

75.6, 75.4, 74.0, 74.0, 73.9, 73.4, 73.4, 72.1, 71.7, 70.8, 70.7, 70.1, 70.0, 69.9, 69.1, 68.7, 67.1, 63.6, 63.2, 60.9, 60.8, 52.8, 52.7, 50.0, 49.8, 49.8, 49.7, 42.4, 34.7, 23.2, 22.8, 22.3, 14.8.
HRMS (ESI) calcd for $C_{54}H_{70}Br_2N_7O_{25}$ $[M-H]^-$: 1374.2788; found 1374.2763.

Lectin Labeling

Stock solutions of WGA/ConA/PNA (117 µM) and CDLPs **6a**–**c** (20 mM) were prepared in PBS and DMSO, respectively. Labeling experiments were performed by combining lectin (75 µM) with varying concentrations of CDLP in a total volume of 50 µL. The resulting samples were incubated at 37 °C for 4 h, then at room temperature in the dark for 1 h after the addition of DBCO-MB488 or DBCO-AF647 (1 mM). Samples were subsequently prepared for SDS-PAGE by adding 18 µL of 4× SDS loading buffer. A 10 µL aliquot of each sample was heated at 90 °C for 5 minutes and subsequently analyzed by SDS-PAGE (12% Bis-Tris, MES running buffer). Fluorescence was imaged at 488 nm or 647 nm before the gel was stained with Coomassie Blue overnight and then imaged again. [Note: Lectin concentrations are reported as protein concentration calculated from the supplier-provided molecular weight.]

Lectin Labeling Competition Experiments

Stock solutions of WGA/ConA/PNA (117 µM) and corresponding sugar (GlcNAc: 750 mM, Gal/Man: 2 M) were prepared in PBS. CDLPs **6a**-**c** (20 mM) were prepared in DMSO. Labeling experiments were performed by combining lectin (75 µM) and CDLP (150 µM) with varying sugar concentrations in a total volume of 50 µL. The resulting samples were incubated at 37 °C for 4 hours, then at room temperature in the dark for 1 hour after addition of DBCO-MB488 (1 mM). Samples were subsequently prepared for SDS-PAGE by adding 18 µL of 4× SDS loading buffer. 10 µL of each sample was heated at 90 °C for 5 minutes and subsequently analyzed by SDS-PAGE (12% Bis-Tris, MES running buffer). Fluorescence was imaged at 488 nm before the gel was stained with Coomassie blue overnight and imaged.

Lectin–CDLP Labeling Time-Course Assays

Time-course labeling experiments were performed to evaluate the kinetics of lectin labeling by matched CDLP probes. The matched lectin–probe pairs were WGA with GlcNAc-CDLP **6a**, ConA with Man-CDLP **6b**, and PNA with Gal-CDLP **6c**. For each reaction, lectin was combined with the corresponding CDLP probe in PBS to final concentrations of 75 µM lectin and 150 µM CDLP probe in a total reaction volume of 150 µL. Reactions were incubated at 37 °C.

At each indicated time point, a 10 µL aliquot was removed and combined with 1 µL of DBCO-AF647 stock solution (1 mM). The click-labeling reaction was incubated for 1 h at room temperature in the dark, after which samples were stored at −30 °C until analysis. Time points analyzed included 0, 0.25, 0.5, 1, 2, 4, 8, 16, and 24 h. Samples were prepared for SDS-PAGE by combining 6 µL of each aliquot with 2 µL of 4× SDS loading buffer, heating at 90 °C for 5 min, and resolving on a 12% Bis-Tris gel using MES running buffer. Gels were imaged for AF647 fluorescence and subsequently stained with Coomassie Blue.

Lectin Selectivity and Competition Assays

Selectivity and competition experiments were performed to evaluate preferential labeling of each lectin by matched and mismatched CDLP probes. Stock solutions of WGA, ConA, and PNA were prepared in PBS at 117 µM. Stock solutions of the corresponding free sugars, N-acetylglucosamine (GlcNAc), mannose, or galactose, were prepared in PBS at 400 mM. CDLP probes **6a**–**6c** were prepared in DMSO at 20 mM.

For each assay, the lectin was combined with the indicated CDLP probe in the absence or presence of the corresponding free-sugar competitor. Final reaction concentrations were 75 µM lectin, 150 µM CDLP probe, and, where included, 132 mM free-sugar competitor, in a total volume of 50 µL. WGA samples were competed with GlcNAc, ConA samples with mannose, and PNA samples with galactose. Reactions were incubated at 37 °C for 4 h.

After incubation, 10 µL of each sample was treated with 1 µL of DBCO-AF647 stock solution (1 mM) and incubated for 1 h at room temperature in the dark. Samples were prepared for SDS-PAGE by combining 6 µL of each aliquot with 2 µL of 4× SDS loading buffer, followed by heating at 90 °C for 5 min. Samples were resolved by SDS-PAGE using a 12% Bis-Tris gel with MES running buffer. Gels were imaged for AF647 fluorescence prior to Coomassie Blue staining.

General RP-HPLC Method for CDLP Stability and Degradation Assays

CDLP stability and degradation were monitored by reversed-phase HPLC using caffeine as an internal standard. Unless otherwise noted, aliquots of CDLP-containing samples were diluted into caffeine-spiked solvent and analyzed directly by RP-HPLC. Chromatographic separation was performed on a Kinetex C18 column, 2.6 µm, 100 Å, 100 × 3 mm. Mobile phase A was 0.1% TFA in H_2O, and mobile phase B was 0.1% TFA in CH_3CN. The gradient was held at 95:5 A:B for 2 min, then ramped to 5:95 A:B over 20 min. The injection volume was 3 µL, and chromatograms were monitored at 254 nm.

Probe degradation was quantified by integration of the parent CDLP peak and caffeine internal standard peak. The parent probe/caffeine peak area ratio was calculated for each time point. Percent degradation was calculated using the following equation:

$$\%\ Degradation = \frac{(Area\ Ratio)_{initial} - (Area\ Ratio)_{final}}{(Area\ Ratio)_{initial}} \times 100$$

where the area ratio corresponds to the integrated parent CDLP peak area divided by the integrated caffeine peak area.

Stability of Lac-CDLP 6e in PBS and in the Presence of N-Acetyllysine Methyl Ester

Lac-CDLP **6e** was prepared at 1 mM in PBS in a total volume of 100 µL. For lysine-reactivity samples, Lac-CDLP **6e** was prepared under the same conditions with N-acetyllysine methyl ester at a final concentration of 2.5 mM. Samples were incubated at 37 °C.

At each indicated time point, 5 µL of sample was removed and diluted to 50 µL with H_2O containing caffeine internal standard. The caffeine-spiked diluent contained 100 µM caffeine. The diluted sample was analyzed directly by RP-HPLC using the general method described above. Samples were analyzed at 0, 4, and 24 h. Degradation products observed in the HPLC chromatograms were assigned by LC-MS analysis of the corresponding samples.

Degradation of Lac-CDLP 6e in the Presence of RNase B

Lac-CDLP **6e** was prepared at 1 mM in PBS containing RNase B at a final concentration of 250 µM in a total volume of 100 µL. Samples were incubated at 37 °C. At each indicated time point, 5 µL of sample was removed and diluted to 50 µL with ice-cold CH_3CN containing caffeine internal standard. The caffeine-spiked CH_3CN contained 100 µM caffeine. Diluted samples were incubated on ice for 10 min and then centrifuged at 16,000 × g for 5 min at 4 °C. The supernatant was collected and analyzed directly by RP-HPLC using the general method described above.

A separate aliquot was removed for fluorescence labeling and SDS-PAGE analysis. For these samples, 1.5 µL of the reaction mixture was diluted to a final volume of 6 µL with DBCO-AF647 solution in PBS and incubated for 1 h at room temperature in the dark. Samples were then prepared for SDS-PAGE analysis as described above. Degradation products observed by HPLC were assigned by LC-MS analysis of the corresponding samples.

Degradation of 6′SL-CDLP 6d in Serum-Free RPMI Medium

6′SL-CDLP **6d** was prepared at 500 µM in serum-free RPMI medium supplemented with 0.45% glucose and 1% penicillin-streptomycin in a total volume of 100 µL. Samples were incubated at 37 °C. At each indicated time point, 5 µL of sample was removed and diluted to 50 µL with H_2O containing 100 µM caffeine and 0.1% TFA. The diluted samples were analyzed directly by RP-HPLC using the general method described above. Time points analyzed included 0, 2, 4, and 16 h.

To evaluate potential interference from media components, serum-free RPMI medium lacking 6′SL-CDLP 6d was processed and analyzed under the same RP-HPLC conditions. Degradation products observed in 6′SL-CDLP 6d samples were assigned by LC-MS analysis of the corresponding samples.

LC-MS Assignment of CDLP Degradation Products

Degradation products observed in the RP-HPLC assays were assigned by LC-MS analysis of the corresponding degradation samples. For Lac-CDLP **6e**, LC-MS was used to verify the formation of degradation products in the presence of N-acetyl lysine methyl ester and RNase B. For 6′SL-CDLP **6d**, LC-MS was used to assign products formed during incubation in serum-free RPMI medium. Proposed assignments were made by comparison of observed ions with calculated masses for hydrolysis products, ligand-release products, and amino acid-associated adducts derived from medium components.

The 6′SL-CDLP **6d** media-degradation products included assignments consistent with adducts or products associated with L-glutamine, L-lysine, L-histidine, L-arginine, L-asparagine, glycine, L-proline, L-valine, L-leucine, or L-isoleucine, as well as hydrolysis and ligand-release products.

Cell Assays and Sample Analysis

Immunoblotting Analysis of CDLP Binding Proteins

Daudi ($CD22^{+}$, RRID: CVCL_0008) and Jurkat ($CD22^{-}$, RRID: CVCL_0367) cells (3×10^6 cells) were incubated with various concentrations (50, 200, and 500 μM) of CDLPs 6d-f in serum-free RPMI medium supplemented with 0.45% glucose and 1% penicillin-streptomycin at 37 °C under 5% CO_2 for 16 h. The cells were harvested and washed three times with PBS. The cells were then incubated with 200 μM DBCO-AF647 in PBS at 4 °C for 2 h. After labeling, the cells were harvested and washed three times with PBS to remove unreacted DBCO-AF647. Cells were lysed using RIPA buffer supplemented with 1× protease inhibitor, and total protein concentrations were determined using the Pierce™ BCA Protein Assay Kit. Cell lysates were mixed with loading buffer and subjected to SDS-PAGE. Proteins were subsequently transferred onto nitrocellulose membranes. The blot was blocked with 5% BSA in Tris-buffered saline containing Tween-20 (TBST) at room temperature for 1 h to prevent nonspecific antibody binding. All antibody dilutions were prepared in TBST containing 5% BSA. Membranes were incubated with anti-CD22 primary antibody (1:1,000) at 4 °C overnight, followed by three washes with TBST (5 minutes per wash). The membranes were then incubated with Alexa Fluor 800-conjugated secondary antibody (1:2,000) at room temperature for 1 h and washed three times with TBST (5 minutes per wash). Next, membranes were incubated with a β-actin polyclonal antibody (1:1,000) at 4 °C overnight, washed three times with TBST (5 minutes per wash), and incubated with either an Alexa Fluor 800-conjugated secondary antibody (1:2,000) or an Alexa Fluor 488-conjugated secondary antibody (1:2,000) at room temperature for 1 h. After three additional washes with TBST (5 minutes per wash), membranes were imaged using the iBright FL1500 Imaging System. Immunoblot data were analyzed using iBright analysis software, and protein expression levels were normalized to β-actin. Statistical significance was assessed using a two-way ANOVA and a Dunnett's multiple-comparisons test. Calculations were carried out using GraphPad PRISM 8. $*P < 0.05$, $**P < 0.01$, $***P < 0.001$, $****P < 0.0001$. To test whether free 6′SL competition reduced 6d labeling, consistent with engagement of the CD22 carbohydrate-binding site, competition experiments were performed by pre-incubating cells with free 6′-sialyllactose (6′SL, 0–2 mM) for 30 minutes at 37 °C under 5% CO_2 prior to the addition of 6′SL-CDLP 6d. Subsequent labeling, lysis, and immunoblotting were performed as described above.

Experiments with CDLP S5 were carried out under identical conditions, except for fluorophore functionalization, which was performed as follows: After treating cells with varying concentrations of S5, cells were washed with PBS. The cells were then mixed with 0.5 mL Click-iT® reaction cocktail, containing AzDye 680 Azide (200 μM), for 30 minutes at room temperature. After labeling, the cells were harvested and washed three times with PBS to remove unreacted AzDye 680 Azide. Subsequently, the cells were handled as described above.

Flow Cytometry Analysis of 6′SL-CDLP Binding Proteins

2×10^5 Daudi ($CD22^+$) and Jurkat ($CD22^-$) cells were harvested and washed three times with PBS. Various concentrations (0, 50, 200, and 500 μM) of 6′SL-CDLP **6d** were prepared in serum-free RPMI medium supplemented with 0.45% glucose and 1% penicillin-streptomycin, and the cells were incubated at 4 °C for 1 h in the dark. After 1 h incubation, excess 6′SL-CDLP **6d** was removed by washing the cells three times with cold HBSS containing 1% (w/v) BSA. Cells were then incubated with 200 μM DBCO-Alexa Fluor 647 (DBCO-AF647) in HBSS at 4 °C for 2 h. Following labeling, the cells were washed three times with cold HBSS containing 1% (w/v) BSA to remove unreacted DBCO-AF647 and stained with 7-aminoactinomycin D (2.5 μg/mL, Tonbo Biosciences) or Ghost 780 (1 μL/mL Tonbo Biosciences) in flow buffer to assess viability. Finally, the stained cells were analyzed by flow cytometry using an Attune™ NxT Flow Cytometer. A minimum of 10,000 viable events per condition were analyzed. Statistical significance was assessed using a two-way ANOVA and a Dunnett's multiple-comparisons test. Calculations were carried out using PRISM 8. *$P < 0.05$, **$P < 0.01$, ***$P < 0.001$, ****$P < 0.0001$.

Live-cell 6′SL-CDLP time-course and washout-persistence assays

Daudi ($CD22^+$) and Jurkat ($CD22^-$) cells were used to evaluate the time-dependent accumulation of 6′SL-CDLP 6d labeling in live cells. For each condition, 2×10^5 cells were harvested, washed three times with PBS, and resuspended in serum-free RPMI medium supplemented with 0.45% glucose and 1% penicillin-streptomycin. 6′SL-CDLP 6d was added at final concentrations of 0, 50, 200, or 500 μM, and cells were incubated at 37 °C under 5% CO_2 in the dark for the indicated times. Time points analyzed were 1, 2, 4, 8, and 16 h.

At each time point, excess 6′SL-CDLP **6d** was removed by washing cells three times with cold HBSS containing 1% (w/v) BSA. CDLP-labeled cells were then incubated with 200 μM DBCO-Alexa Fluor 647 (DBCO-AF647) in HBSS at 4 °C for 2 h in the dark to fluorescently functionalize azide-tagged proteins by SPAAC. Following DBCO-AF647 labeling, cells were washed three times with cold HBSS containing 1% (w/v) BSA to remove unreacted fluorophore. Cells were then stained with 7-aminoactinomycin D (7-AAD; 2.5 μg/mL, Tonbo Biosciences) or Ghost 780 viability dye (1 μL/mL, Tonbo Biosciences) in flow buffer to assess viability.

For post-wash persistence experiments, Daudi cells were harvested, washed three times with PBS, and treated with 500 μM 6′SL-CDLP 6d in serum-free RPMI medium supplemented with 0.45% glucose and 1% penicillin-streptomycin at 37 °C under 5% CO_2 in the dark for 16 h. After probe treatment, cells were washed three times with cold HBSS containing 1% (w/v) BSA to remove excess 6′SL-CDLP **6d**, then returned to probe-free complete RPMI medium. Cells were maintained at 37 °C under 5% CO_2 and analyzed at 0, 24, 48, and 72 h after probe washout. At each post-wash time point, cells were harvested, washed, functionalized with 200 μM DBCO-AF647 in HBSS at 4 °C for 2 h in the dark, washed three times with cold HBSS containing 1% (w/v) BSA, and stained with 7-AAD or Ghost 780 viability dye as described above.

Samples were analyzed on an Attune™ NxT Flow Cytometer. Cells were first gated by forward- and side-scatter to exclude debris, then singlet-gated and nonviable cells were excluded using 7-AAD or Ghost 780 staining. AF647 median fluorescence intensity (MFI) was quantified from viable singlet cells. At least 10,000 viable events were collected for each condition. Time-course experiments were performed with three biological replicates, and post-wash persistence experiments were performed with four biological replicates. Data are plotted as individual biological replicates with mean ± SEM.

For the time-course analysis, statistical significance was assessed using a two-way repeated-measures ANOVA with Geisser–Greenhouse correction, followed by Tukey's multiple-comparisons test within each time point. For the post-wash persistence analysis, statistical significance was assessed using an ordinary one-way ANOVA followed by Dunnett's multiple-comparisons test comparing each post-wash time point to the 0 h time point. Statistical analyses were performed using GraphPad Prism 8. For all analyses, *$p < 0.05$, **$p < 0.01$, ***$p < 0.001$, and ****$p < 0.0001$.

Proteomic Profiling

Cell Treatment and Lysate Preparation

15 million Daudi cells were harvested and washed three times with PBS. Cells were treated separately with 6′SL-CDLP **6d** or Lac-CDLP **6e** at final concentrations of 500 μM and incubated in serum-free RPMI medium supplemented with 0.45% glucose and 1% penicillin-streptomycin at 37 °C under 5% CO_2 for 16 h. Three biological replicates were prepared for each condition. Following incubation, the cells were washed three times with PBS and subsequently reacted with 200 μM DBCO-PEG4-desthiobiotin in PBS at 4 °C for 2 h. After labeling, the cells were washed three times with PBS to remove unreacted DBCO-PEG4-desthiobiotin. Cells were lysed using RIPA buffer supplemented with 1× protease inhibitor, and total protein concentrations were quantified using the Pierce™ BCA Protein Assay Kit. For each condition, 1 mg of total protein was denatured, reduced, and alkylated. Disulfide bonds were reduced with tris(2-carboxyethyl)phosphine (TCEP; 10 mM final concentration) at 37 °C for 1 h in the dark, followed by alkylation with iodoacetamide (IAA; 40 mM final concentration) for 1 h at room temperature in the dark. TCEP and IAA solutions were prepared in 6 M urea and 100 mM ammonium bicarbonate. Then, TCEP and IAA were removed by buffer exchange into PBS using a 7 kDa MWCO desalting column.

To assess carbohydrate-specific binding of CDLPs, competition experiments were performed by pre-incubating cells with 30 mM of free 6′-sialyllactose (6′SL) or free lactose for 30 minutes at 37 °C under 5% CO_2 prior to the addition of 6′SL-CDLP **6d** or Lac-CDLP **6e**, respectively. Subsequent labeling, cell lysis, and lysate denaturation, reduction, and alkylation were performed as described above.

Streptavidin-Desthiobiotin Enrichment of CDLP-Binding Proteins

500 μL of streptavidin beads (Pierce™ Streptavidin Magnetic Beads) were washed three times with PBS. Denatured cell lysates, prepared as described above, were incubated with the beads at 4 °C overnight with gentle mixing to capture desthiobiotin-tagged proteins. The following day, the beads were immobilized using a magnetic rack, and the unbound protein was discarded. To remove non-specifically bound proteins, bead-bound proteins were subjected to sequential washing steps: three times with 1% SDS in PBS, three times with 6 M urea in 100 mM ammonium bicarbonate, three times with 1 M NaCl in PBS, and three times with 0.2% SDS in 50 mM HEPES (pH 8). Each wash was performed for 5 minutes with gentle rotation. After the washing step, proteins were eluted by incubating the beads with 40 μL of elution buffer (50 mM Tris, pH 8, 2% SDS, 20 mM DTT, and 2 mM biotin) at 37 °C for 4 h with rotation. Eluted samples were separated from the beads using a magnetic rack and collected for downstream analysis. Eluted samples were either analyzed by immunoblotting as described above or submitted for mass spectrometry-based proteomic analysis.

For streptavidin-enrichment validation experiments, CDLP-treated cells were functionalized with a desthiobiotin-affinity tag via SPAAC prior to streptavidin enrichment. Enriched fractions were separated by SDS-PAGE and transferred to nitrocellulose membranes. Total enriched CDLP-tagged proteins were detected by incubating the membranes with Alexa Fluor 647-conjugated streptavidin and then imaging the fluorescence. The same membranes, or parallel membranes prepared from the same enriched samples, were analyzed by target-specific immunoblotting using antibodies against CD22 or selected chemoproteomic candidate proteins. Thus, the AF647 channel in Figures S12 and S19 reflects streptavidin-AF647 detection of desthiobiotin-affinity-tagged proteins and does not correspond to DBCO-AF647 click labeling.

Sample Preparation for LC-MS/MS Analysis

Sample volumes were brought up to 50 μL using 50 mM TEAB. Disulfides were reduced by adding 5 μL of 50 mM TCEP (5 mM final) and incubating at 55 °C for 30 minutes. Cysteines were alkylated by adding 1.5 μL of 375 mM IAA (10 mM final) and incubating at room temperature in the dark for 30 minutes. Proteins were precipitated by adding 225 μL of cold acetone (4.5 volumes; final ~82% acetone) and incubating at −20 °C overnight. Samples were centrifuged at 14,000 × *g* for 10 minutes at 4 °C to pellet the proteins. The supernatant was removed, and proteins were air-dried on the benchtop for 15 minutes. Proteins were resuspended in 100

μL of 50 mM TEAB, pH 8, and 2 mM $CaCl_2$ and digested by incubating with 400 ng of trypsin (0.1 mg/mL) overnight at 37 °C and 500 rpm (Thermomixer, Eppendorf). Reactions were quenched by the addition of 10% formic acid (FA) to a final concentration of 1%. Digested samples were centrifuged at 10,000 × g for 10 minutes to remove particulates, and the supernatant was transferred to a fresh tube. Peptide concentrations were determined using a NanoDrop UV spectrophotometer (Thermo) at 205 nm.

LC-MS/MS Detection and Data Analysis

Samples were injected onto a Vanquish Neo (Thermo) nano-UPLC C18 trap column (PepMap Neo, 0.3 mm × 5 mm, 5 μm) under pressure loading. Peptides were eluted onto the separation column (Aurora Elite, 15 cm × 75 μm, 1.7 μm C18 particle size, IonOpticks™) heated to 40 °C prior to elution directly to the Orbitrap Ascend Tribrid MS (Thermo) equipped with a high-field asymmetric ion mobility spectrometry (FAIMS) source.[60] Briefly, peptides were loaded and washed for 5 minutes at a flow rate of 0.4 μL/min at 2% B (mobile phase A: 0.1% FA in water, mobile phase B: 80% CH_3CN, 0.1% FA in water). Peptides were eluted over 100 minutes from 2–25% mobile phase B, then ramped to 40% B in 20 minutes. The column was washed for 15 minutes at 100% B, then re-equilibrated to 2% B for the next injection. The data were collected by data-dependent acquisition, with the intact peptide detected in the Orbitrap at 120,000 resolving power from *m/z* 375 to 1,500. Peptides with a charge of +2 to +7 were selected for fragmentation by higher-energy collision dissociation (HCD) at 28% NCE and were detected in the ion trap using a rapid scan rate. Dynamic exclusion was set to 60 s after one instance. The mass list was shared between the FAIMS compensation voltages. FAIMS voltages were set at -45 V (1.4 s), -60 V (1 s), -75 V (0.6 s) for a total duty cycle time of 3 s. Source ionization was set to +1700 V, and the ion transfer tube temperature was set to 305 °C. Raw files were searched against the human protein database downloaded from UniProt on 03-21-2025 using SEQUEST in Proteome Discoverer 3.0.[77] Abundances, abundance ratios, and p-values were exported to Microsoft Excel for further analysis.

Protein-level abundances, abundance ratios, and abundance-ratio p-values were exported from Proteome Discoverer for downstream analysis in Microsoft Excel. Proteins were retained for candidate analysis if they were detected in at least two biological replicates within the relevant ligand-specific sample set. Competition-sensitive enrichment was calculated as $\log_2$[(probe)/(probe + competitor)] from the exported abundance ratios. Candidate proteins were prioritized using $\log_2$[(probe)/(probe + competitor)] ≥ 1 and nominal $p \leq 0.05$. Protein-level p-values were not adjusted for multiple testing; therefore, $p \leq 0.05$ is treated as a nominal threshold, and competition-sensitive candidate proteins are described as competition-sensitive candidate proteins rather than FDR-significant hits. No additional statistical testing or missing-value imputation was performed in Excel after export from Proteome Discoverer. Missing-value handling, if any, followed the Proteome Discoverer processing/export settings.

Data Availability Statement

The mass spectrometry data have been deposited to MassIVE (https://massive.ucsd.edu/). The accession number for the data reported in this paper is MSV000098943. It has also been submitted to ProteomeXchange (PXD067763).

Assessment of Prior Carbohydrate-Related Annotation

Competition-sensitive candidate proteins were assessed for prior “carbohydrate-related annotation” using a combined UniProtKB/GO/CAZy workflow. Briefly, the competition-sensitive candidate proteins (Tables S3–S4; unique UniProt accessions) were mapped to their UniProtKB entries (same UniProtKB release used for proteomics database searching, accessed on the analysis date). For each protein, we extracted: (i) UniProtKB Keywords; (ii) Gene Ontology (GO) annotations (molecular function and biological process); and (iii) CAZy

annotations (CAZy family assignment/cross-reference, when present). A protein was classified as carbohydrate-related if any of the following conditions were met:

1. UniProtKB contained a keyword consistent with carbohydrate/glycan binding (e.g., lectin/carbohydrate-binding/glycosaminoglycan- or heparin-binding) and/or glycan-active function (e.g., glycosyltransferase/glycosidase or related glycan-modifying activity); or
2. The UniProtKB-associated GO terms included functions/processes consistent with glycan binding (e.g., carbohydrate/glycan/glycoprotein/glycosaminoglycan binding) and/or glycan biosynthesis/modification/catabolism; or
3. The protein was assigned to a CAZy family (e.g., GT/GH/PL/CE/AA/CBM classes).

Proteins that lacked all three annotation types above were classified as "without prior carbohydrate-related annotation". This designation reflects the absence of curated carbohydrate/glycan-related functional annotation in these resources at the time of analysis and does not exclude the possibility of unrecognized or context-dependent glycan interactions.

STRING Network Analysis of CDLP-Captured Protein Profiles

To evaluate whether the competition-sensitive CDLP-captured protein profiles formed biologically structured networks, we analyzed the 6′SL-CDLP- and Lac-CDLP-competition-sensitive candidate protein sets using STRING/stringApp in Cytoscape. Networks were generated using the full STRING functional association network, with the organism set to Homo sapiens, a confidence cutoff of 0.7, and no added interactors. The CDLP-detected non-contaminant proteome was used as a custom enrichment background. This background contained 4,616 input gene symbols, of which 4,567 were represented in the STRING/stringApp background network.

The full 6′SL-CDLP set contained 95 competition-sensitive candidate proteins, of which 94 were represented in the STRING network. The KGD4 hit was represented using the STRING-recognized gene symbol MRPS36, and IFNAR2-IL10RB was queried using the Ensembl protein identifier ENSP00000388223, which resolved to the IL10RB/I10R2_HUMAN node in stringApp. HLA-DRB3/P79483 was not represented in the stringApp network and was excluded from STRING-based network and enrichment analyses. The full Lac-CDLP set contained 113 competition-sensitive candidate proteins, all of which were represented in the STRING network.

Full CDLP profiles showed significant PPI enrichment, indicating that the recovered proteins were more connected than expected by chance. The 6′SL-CDLP full network showed PPI enrichment of $p = 1.67 \times 10^{-12}$, and the Lac-CDLP full network showed PPI enrichment of $p = 9.1 \times 10^{-13}$. Probe-selective subsets also remained significantly PPI-enriched after removal of shared hits, with 6′SL-only $p = 7.52 \times 10^{-11}$ and Lac-only $p = 2.8 \times 10^{-9}$. In contrast, the 21-protein shared core was not significantly PPI-enriched in the supplied STRING run ($p = 0.1126$), although it retained enriched annotation terms associated with glycoproteins, signal peptides, immunoglobulin-like domains, receptors, and transmembrane proteins.

Functional enrichment analysis showed distinct biological signatures for the two CDLP profiles. The 6′SL-CDLP full and 6′SL-only sets were enriched for cell-surface, plasma membrane, adhesion, immunoglobulin domain, receptor, and immune-associated terms. In contrast, the Lac-CDLP full and Lac-only sets were enriched for glycosyltransferase activity, glycoprotein metabolic and biosynthetic processes, O-glycan biosynthesis, glycosaminoglycan metabolism, mitochondrial matrix, and broader metabolic terms. These results support the conclusion that 6′SL-CDLP and Lac-CDLP yield distinct, biologically structured, competition-sensitive capture profiles under the applied live-cell labeling conditions.

Importantly, STRING network connectivity and term enrichment do not establish direct glycan binding for every competition-sensitive candidate protein. Instead, these analyses provide a network-level prioritization framework for interpreting CDLP-captured candidates. Because 6′SL-CDLP and Lac-CDLP differ in glycan structure as well as size, charge, and likely lipophilicity, the observed enrichment and localization profiles should be interpreted as probe-captured profiles shaped by ligand recognition together with probe physicochemical properties, uptake/trafficking, and the 16 h labeling window.

Supplementary Tables

Table S1. LC-MS assignments of 6′SL-CDLP 6d degradation products formed in media

Retention time (min)	Proposed assignment	Neutral exact mass shown (Da)	Expected $[M+H]^+$	Observed m/z	Notes
3.936	L-glutamine or L-lysine adduct	552.2544/552.2908	553.26/553.30	553.15	Near-isobaric assignments; not distinguished by the QDa scan alone
3.936	L-histidine adduct	561.2547	562.26	562.11	Co-eluting assignment observed in the same scan
4.162	L-arginine adduct	580.2969	581.30	581.17	Amino acid adduct
4.348	L-glutamine or L-lysine adduct	552.2544/552.2908	553.26/553.30	553.16	Second chromatographic feature assigned to glutamine/lysine-type adduct
4.581	L-asparagine adduct	538.2387	539.25	539.08	Additional ion at m/z 561.11 is consistent with a sodium-associated ion
4.732	L-glutamine or L-lysine adduct	552.2544/552.2908	553.26/553.30	553.13	Third chromatographic feature assigned to glutamine/lysine-type adduct
5.130	Glycine adduct	481.2173	482.22	482.15	Amino acid adducts
5.267	Ligand-release product	1121.1811	1122.19	1124.05	Observed ion likely corresponds to the bromine-containing isotope cluster rather than the monoisotopic peak
5.957	Probe hydrolysis product	424.1958	425.20	425.15	Additional ion at m/z 447.12 is consistent with a sodium-associated ion
6.132	Proline adduct	521.2486	522.26	522.13	Additional ion at m/z 544.07 is consistent with a sodium-associated ion
7.031	L-valine adduct	523.2642	524.27	524.14	Additional ion at m/z 546.08 is consistent with a sodium-associated ion
7.855	L-leucine, L-isoleucine adduct	537.2799/537.2435	538.29/538.25	538.17	Isomeric/near-isobaric assignments; not distinguished by QDa scan alone
8.026	L-leucine, L-isoleucine adduct	537.2799/537.2435	538.29/538.25	538.16	Second chromatographic feature with the same nominal assignment

Observed m/z values were obtained from QDa positive-mode scans of 6′SL-CDLP 6d incubated in serum-free RPMI medium. Expected $[M+H]^+$ values were calculated from the neutral exact masses annotated in the LC-MS figure. Assignments involving near-isobaric or isomeric amino acids, including glutamine/lysine and leucine/isoleucine, should be considered putative without authentic standards or MS/MS confirmation.

Table S2. Residue distribution of dibromophenyl benzoate-modified peptide entries

Localization class	Count	Percent of modified peptide entries
Lys only	406	80.1%
Ser only	25	4.9%
Thr only	17	3.4%
Tyr only	12	2.4%
His only	4	0.8%
Ambiguous mixed localizations	43	8.5%
Total	507	100%

Ambiguous localization class (count)	Ambiguous localization class (count)
K/S (20)	K/Y (6)
K/T (6)	H/K (4)
T/Y (2)	S/Y (1)
H/Y (1)	K/S/T (1)
K/T/Y (1)	H/K/Y (1)

Residue assignments were tabulated from the “Modifications” column of Appendix 1 by extracting the residue annotation associated with “Dibromophenyl Benzoate.” Entries such as [K17], [S3], [T4], [Y2], and [H5] were counted as unambiguous Lys, Ser, Thr, Tyr, or His localizations, respectively. Entries reported as mixed residue classes, such as [K/S], [K/T], [K/Y], or [H/K], were counted separately as ambiguous mixed localizations. Counts are reported per modified peptide entry, not per unique protein, unique peptide sequence, or PSM. These data indicate that dibromophenyl benzoate adduct localization is strongly lysine-favored under the present analysis conditions but is not restricted to lysine.

Table S3. Competition-Sensitive Protein Candidates from 6′SL-CDLP 6d Profiling

Accession	Gene	Functional Group*
Q9NR28	DIABLO	Apoptosis & cell-death regulators
O14737	PDCD5	Apoptosis & cell-death regulators
E7EWD3	ADAM23	Cell adhesion & structural cell-surface receptors
Q13740	ALCAM	Cell adhesion & structural cell-surface receptors
X5DQS5	CADM1	Cell adhesion & structural cell-surface receptors
P14209	CD99	Cell adhesion & structural cell-surface receptors
P78310	CXADR	Cell adhesion & structural cell-surface receptors
Q14118	DAG1	Cell adhesion & structural cell-surface receptors
P98172	EFNB1	Cell adhesion & structural cell-surface receptors
Q6PCB8	EMB	Cell adhesion & structural cell-surface receptors
Q9Y624	F11R	Cell adhesion & structural cell-surface receptors
Q8TB96	ITFG1	Cell adhesion & structural cell-surface receptors
P13612	ITGA4	Cell adhesion & structural cell-surface receptors
Q9H1U4	MEGF9	Cell adhesion & structural cell-surface receptors
O95297	MPZL1	Cell adhesion & structural cell-surface receptors
Q92859	NEO1	Cell adhesion & structural cell-surface receptors
Q9Y639	NPTN	Cell adhesion & structural cell-surface receptors
P16284	PECAM1	Cell adhesion & structural cell-surface receptors
O15031	PLXNB2	Cell adhesion & structural cell-surface receptors
Q5JXI8	FHL1	Chaperones, cytoskeleton & trafficking
O00461	GOLIM4	Chaperones, cytoskeleton & trafficking
P61604	HSPE1	Chaperones, cytoskeleton & trafficking
Q9UK76	JPT1	Chaperones, cytoskeleton & trafficking
O60925	PFDN1	Chaperones, cytoskeleton & trafficking
Q9HBM1	SPC25	Chaperones, cytoskeleton & trafficking
Q96JI7	SPG11	Chaperones, cytoskeleton & trafficking
P16949	STMN1	Chaperones, cytoskeleton & trafficking
E5RJD8	TBCA	Chaperones, cytoskeleton & trafficking
P20273	CD22	Glycan-binding lectins & GAG-binding receptors
P20138	CD33	Glycan-binding lectins & GAG-binding receptors
Q9UKY7	CDV3	Glycan-binding lectins & GAG-binding receptors
O60449	LY75	Glycan-binding lectins & GAG-binding receptors
P20916	MAG	Glycan-binding lectins & GAG-binding receptors
Q13332	PTPRS	Glycan-binding lectins & GAG-binding receptors
Q96LC7	SIGLEC-10	Glycan-binding lectins & GAG-binding receptors
Q9UHN6	CEMIP2	Glycan-modifying enzymes
Q9NRB3	CHST12	Glycan-modifying enzymes
O95461	LARGE1	Glycan-modifying enzymes
Q6NSJ0	MYORG	Glycan-modifying enzymes
P15529	CD46	Immune receptors & complement/MHC/Fc system
B1AP13	CD55	Immune receptors & complement/MHC/Fc system
Q9UIB8	CD84	Immune receptors & complement/MHC/Fc system

P20023	CR2	Immune receptors & complement/MHC/Fc system
P31994	FCGR2B	Immune receptors & complement/MHC/Fc system
Q96LA6	FCRL1	Immune receptors & complement/MHC/Fc system
A0A140T9N7	HLA-DMA	Immune receptors & complement/MHC/Fc system
P79483	HLA-DRB3	Immune receptors & complement/MHC/Fc system
P17181	IFNAR1	Immune receptors & complement/MHC/Fc system
H0Y3Z8	IFNAR2-IL10RB	Immune receptors & complement/MHC/Fc system
P11215	ITGAM	Immune receptors & complement/MHC/Fc system
A0A0G2JQ15	LILRB1	Immune receptors & complement/MHC/Fc system
Q96DU3	SLAMF6	Immune receptors & complement/MHC/Fc system
G3V2U7	ACYP1	Mitochondrial & metabolic enzymes
Q9NZK5	ADA2	Mitochondrial & metabolic enzymes
P54709	ATP1B3	Mitochondrial & metabolic enzymes
Q6P587	FAHD1	Mitochondrial & metabolic enzymes
P00505	GOT2	Mitochondrial & metabolic enzymes
Q96CN7	ISOC1	Mitochondrial & metabolic enzymes
P82909	KGD4	Mitochondrial & metabolic enzymes
P40926	MDH2	Mitochondrial & metabolic enzymes
Q7RTP0	NIPA1	Mitochondrial & metabolic enzymes
P62341	SELENOT	Mitochondrial & metabolic enzymes
A0A8V8TLM6	TFRC	Mitochondrial & metabolic enzymes
Q9C005	DPY30	Nuclear, chromatin & RNA-associated proteins
A6NMX2	EIF4E1B	Nuclear, chromatin & RNA-associated proteins
Q8WW12	PCNP	Nuclear, chromatin & RNA-associated proteins
H7C2N1	PTMA	Nuclear, chromatin & RNA-associated proteins
P54725	RAD23A	Nuclear, chromatin & RNA-associated proteins
P62306	SNRPF	Nuclear, chromatin & RNA-associated proteins
Q6P179	ERAP2	Proteases & protease inhibitors
O43240	KLK10	Proteases & protease inhibitors
Q8IW75	SERPINA12	Proteases & protease inhibitors
O75635	SERPINB7	Proteases & protease inhibitors
P03973	SLPI	Proteases & protease inhibitors
H0YAX8	TMPRSS4	Proteases & protease inhibitors
U3KQB3	CSNK1G1	Signaling receptors, kinases & adaptor/scaffold proteins
B8ZWD1	DBI	Signaling receptors, kinases & adaptor/scaffold proteins
Q8NDZ4	DIPK2A	Signaling receptors, kinases & adaptor/scaffold proteins
A0A590UJD9	DLG1	Signaling receptors, kinases & adaptor/scaffold proteins
Q5T5H1	ENSA	Signaling receptors, kinases & adaptor/scaffold proteins
P48060	GLIPR1	Signaling receptors, kinases & adaptor/scaffold proteins
Q6UXM1	LRIG3	Signaling receptors, kinases & adaptor/scaffold proteins
P14174	MIF	Signaling receptors, kinases & adaptor/scaffold proteins
Q7Z6M3	MILR1	Signaling receptors, kinases & adaptor/scaffold proteins
Q92542	NCSTN	Signaling receptors, kinases & adaptor/scaffold proteins
P46531	NOTCH1	Signaling receptors, kinases & adaptor/scaffold proteins

Q04721	NOTCH2	Signaling receptors, kinases & adaptor/scaffold proteins
Q96HC4	PDLIM5	Signaling receptors, kinases & adaptor/scaffold proteins
A0A8V8TP51	PTPRJ	Signaling receptors, kinases & adaptor/scaffold proteins
Q9H3S1	SEMA4A	Signaling receptors, kinases & adaptor/scaffold proteins
Q92854	SEMA4D	Signaling receptors, kinases & adaptor/scaffold proteins
O75368	SH3BGRL	Signaling receptors, kinases & adaptor/scaffold proteins
Q9H299	SH3BGRL3	Signaling receptors, kinases & adaptor/scaffold proteins
Q6UWD8	C16orf54	Uncharacterized / other
K7EK56	C19orf25	Uncharacterized / other

*Functional grouping was done using a combination of UniProt keyword(s)/GO terms/CAZy annotation.

Table S4. Competition-Sensitive Protein Candidates from Lac-CDLP 6e Profiling

Accession	Gene	Functional Group*
O76095	JTB	Apoptosis, autophagy & stress response
Q8IVP5	FUNDC1	Apoptosis, autophagy & stress response
A0A3B3IRL2	CREG1	Apoptosis, autophagy & stress response
P48060	GLIPR1	Apoptosis, autophagy & stress response
A0AAQ5BGP1	DSC2	Cell adhesion & cell-surface interaction
P16284	PECAM1	Cell adhesion & cell-surface interaction
O94985	CLSTN1	Cell adhesion & cell-surface interaction
Q8TB96	ITFG1	Cell adhesion & cell-surface interaction
Q9H1U4	MEGF9	Cell adhesion & cell-surface interaction
A0A994J439	ITIH3	Cell adhesion & cell-surface interaction
Q9H0X4	FAM234A	Cell adhesion & cell-surface interaction
P61604	HSPE1	Chaperones, trafficking & cytoskeleton
A0AAG2TEA1	CANX	Chaperones, trafficking & cytoskeleton
Q9Y5U9	IER3IP1	Chaperones, trafficking & cytoskeleton
P11021	HSPA5	Chaperones, trafficking & cytoskeleton
Q8N129	CNPY4	Chaperones, trafficking & cytoskeleton
Q6NSJ0	MYORG	Glycan biosynthesis, modification & catabolism
P04066	FUCA1	Glycan biosynthesis, modification & catabolism
Q01459	CTBS	Glycan biosynthesis, modification & catabolism
P20933	AGA	Glycan biosynthesis, modification & catabolism
P54802	NAGLU	Glycan biosynthesis, modification & catabolism
O00587	MFNG	Glycan biosynthesis, modification & catabolism
P52848	NDST1	Glycan biosynthesis, modification & catabolism
O95461	LARGE1	Glycan biosynthesis, modification & catabolism
P15291	B4GALT1	Glycan biosynthesis, modification & catabolism
H0YAH3	GALNT7	Glycan biosynthesis, modification & catabolism
O60512	B4GALT3	Glycan biosynthesis, modification & catabolism
Q8NBJ5	COLGALT1	Glycan biosynthesis, modification & catabolism
Q10472	GALNT1	Glycan biosynthesis, modification & catabolism
Q96A29	SLC35C1	Glycan biosynthesis, modification & catabolism
Q8N6G5	CSGALNACT2	Glycan biosynthesis, modification & catabolism
Q11201	ST3GAL1	Glycan biosynthesis, modification & catabolism
A0A8I5KNB7	POMGNT1	Glycan biosynthesis, modification & catabolism
Q9NYU2	UGGT1	Glycan biosynthesis, modification & catabolism
Q14697	GANAB	Glycan biosynthesis, modification & catabolism
A0A8I5KTR7	EDEM3	Glycan biosynthesis, modification & catabolism
Q96LC7	SIGLEC-10	Glycan-binding lectins & GAG-binding receptors
G8JL96	PTPRS	Glycan-binding lectins & GAG-binding receptors
P15260	IFNGR1	Immune receptors & complement/MHC/Fc system
H0Y3Z8	IFNAR2-IL10RB	Immune receptors & complement/MHC/Fc system
P04839	CYBB	Immune receptors & complement/MHC/Fc system
P17181	IFNAR1	Immune receptors & complement/MHC/Fc system

Q96LA6	FCRL1	Immune receptors & complement/MHC/Fc system
Q92854	SEMA4D	Immune receptors & complement/MHC/Fc system
A0A8V8TP51	PTPRJ	Immune receptors & complement/MHC/Fc system
P32970	CD70	Immune receptors & complement/MHC/Fc system
P08575	PTPRC	Immune receptors & complement/MHC/Fc system
B7ZKQ9	SCARB1	Immune receptors & complement/MHC/Fc system
O14763	TNFRSF10B	Immune receptors & complement/MHC/Fc system
O95498	VNN2	Immune receptors & complement/MHC/Fc system
Q9ULF5	SLC39A10	Membrane transporters & vesicle trafficking
Q9UHW9	SLC12A6	Membrane transporters & vesicle trafficking
Q9Y2Q0	ATP8A1	Membrane transporters & vesicle trafficking
P19634	SLC9A1	Membrane transporters & vesicle trafficking
O15439	ABCC4	Membrane transporters & vesicle trafficking
Q96DM3	RMC1	Membrane transporters & vesicle trafficking
Q9UFN0	NIPSNAP3A	Membrane transporters & vesicle trafficking
P61019	RAB2A	Membrane transporters & vesicle trafficking
A0A0G2JN29	NOMO1	Membrane transporters & vesicle trafficking
Q5JPE7	NOMO2	Membrane transporters & vesicle trafficking
Q9BV40	VAMP8	Membrane transporters & vesicle trafficking
A0A8Q3SIA0	TIMM17B	Mitochondrial & metabolic enzymes
Q96BP2	CHCHD1	Mitochondrial & metabolic enzymes
P04179	SOD2	Mitochondrial & metabolic enzymes
H0YIC4	CS	Mitochondrial & metabolic enzymes
Q16595	FXN	Mitochondrial & metabolic enzymes
P00505	GOT2	Mitochondrial & metabolic enzymes
Q6NVY1	HIBCH	Mitochondrial & metabolic enzymes
P40926	MDH2	Mitochondrial & metabolic enzymes
P78540	ARG2	Mitochondrial & metabolic enzymes
P07954	FH	Mitochondrial & metabolic enzymes
O15382	BCAT2	Mitochondrial & metabolic enzymes
Q16854	DGUOK	Mitochondrial & metabolic enzymes
E9PF18	HADH	Mitochondrial & metabolic enzymes
P50440	GATM	Mitochondrial & metabolic enzymes
Q9NWU1	OXSM	Mitochondrial & metabolic enzymes
Q12931	TRAP1	Mitochondrial & metabolic enzymes
P55809	OXCT1	Mitochondrial & metabolic enzymes
Q8NCN5	PDPR	Mitochondrial & metabolic enzymes
Q8TB37	NUBPL	Mitochondrial & metabolic enzymes
Q9UKU7	ACAD8	Mitochondrial & metabolic enzymes
Q8NCC3	PLA2G15	Mitochondrial & metabolic enzymes
P42126	ECI1	Mitochondrial & metabolic enzymes
Q13011	ECH1	Mitochondrial & metabolic enzymes
Q9NZK5	ADA2	Mitochondrial & metabolic enzymes
Q9BW60	ELOVL1	Mitochondrial & metabolic enzymes

O95479	H6PD	Mitochondrial & metabolic enzymes
P59103	DAOA	Mitochondrial & metabolic enzymes
Q6YP21	KYAT3	Mitochondrial & metabolic enzymes
Q9NPJ3	ACOT13	Mitochondrial & metabolic enzymes
G3V4T6	GSTZ1	Mitochondrial & metabolic enzymes
Q96CN7	ISOC1	Mitochondrial & metabolic enzymes
O15229	KMO	Mitochondrial & metabolic enzymes
H7C3T0	COPS6	Nuclear, chromatin, RNA & cell cycle
Q9HBM1	SPC25	Nuclear, chromatin, RNA & cell cycle
Q04837	SSBP1	Nuclear, chromatin, RNA & cell cycle
O14578	CIT	Nuclear, chromatin, RNA & cell cycle
Q13112	CHAF1B	Nuclear, chromatin, RNA & cell cycle
P62306	SNRPF	Nuclear, chromatin, RNA & cell cycle
A0A1B0GVL4	FAM193A	Nuclear, chromatin, RNA & cell cycle
Q9NRG0	CHRAC1	Nuclear, chromatin, RNA & cell cycle
Q7L2Z9	CENPQ	Nuclear, chromatin, RNA & cell cycle
A6NMX2	EIF4E1B	Nuclear, chromatin, RNA & cell cycle
A0A8I5KXC3	MOV10	Nuclear, chromatin, RNA & cell cycle
Q9NZI8	IGF2BP1	Nuclear, chromatin, RNA & cell cycle
Q9NQH7	XPNPEP3	Proteases, nucleases & protease inhibitors
O00115	DNASE2	Proteases, nucleases & protease inhibitors
Q6UWW8	CES3	Proteases, nucleases & protease inhibitors
Q99574	SERPINI1	Proteases, nucleases & protease inhibitors
G3V180	DPP3	Proteases, nucleases & protease inhibitors
O60262	GNG7	Signaling receptors, kinases & adaptor/scaffold proteins
P36894	BMPR1A	Signaling receptors, kinases & adaptor/scaffold proteins
Q4KWH8	PLCH1	Signaling receptors, kinases & adaptor/scaffold proteins

*Functional grouping was done using a combination of UniProt keyword(s)/GO terms/CAZy annotation.

NMR Spectra

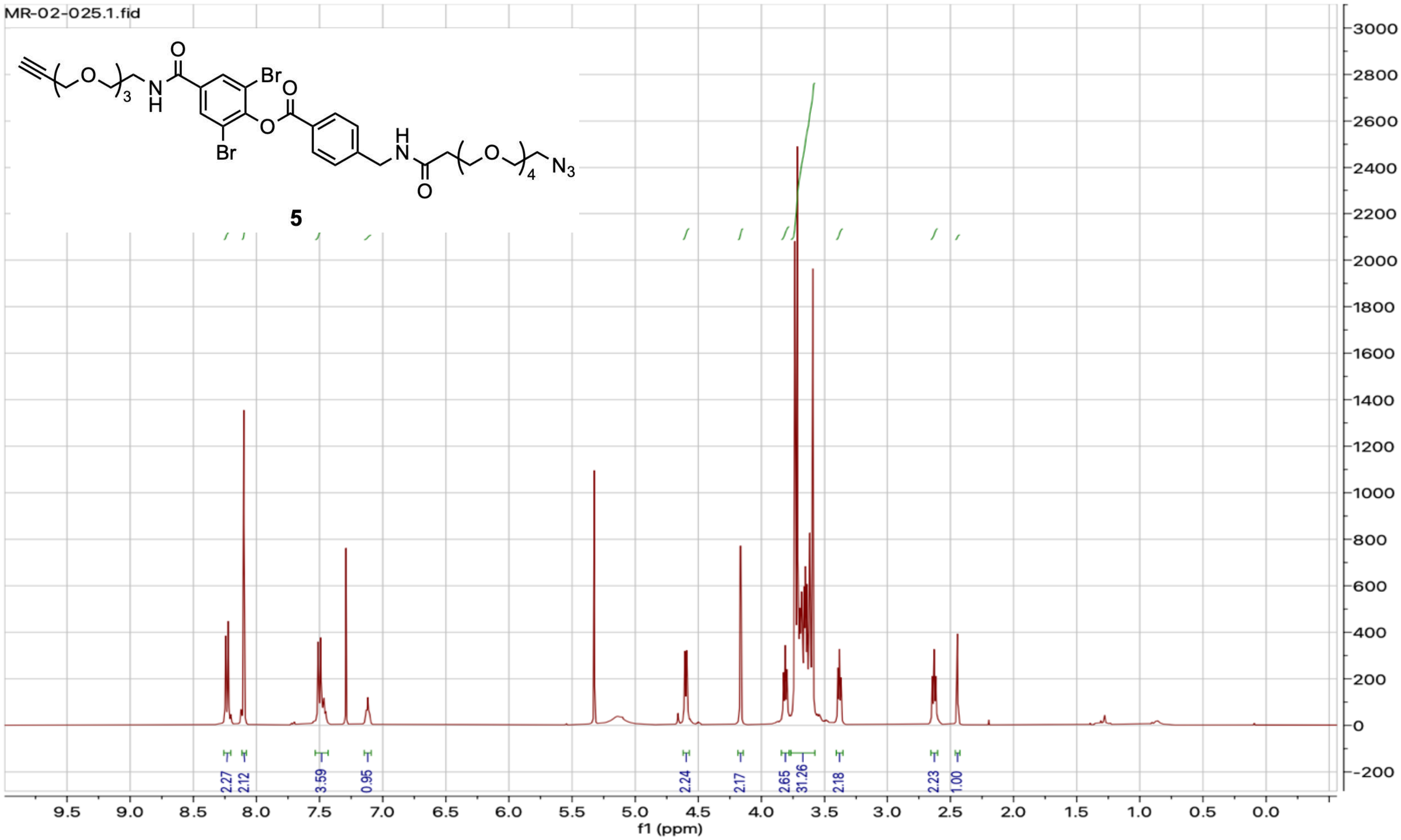
MR-02-025.1.fid
5
2.27
2.12
3.59
0.95
2.24
2.17
2.65
31.26
2.18
2.23
1.00
f1 (ppm)

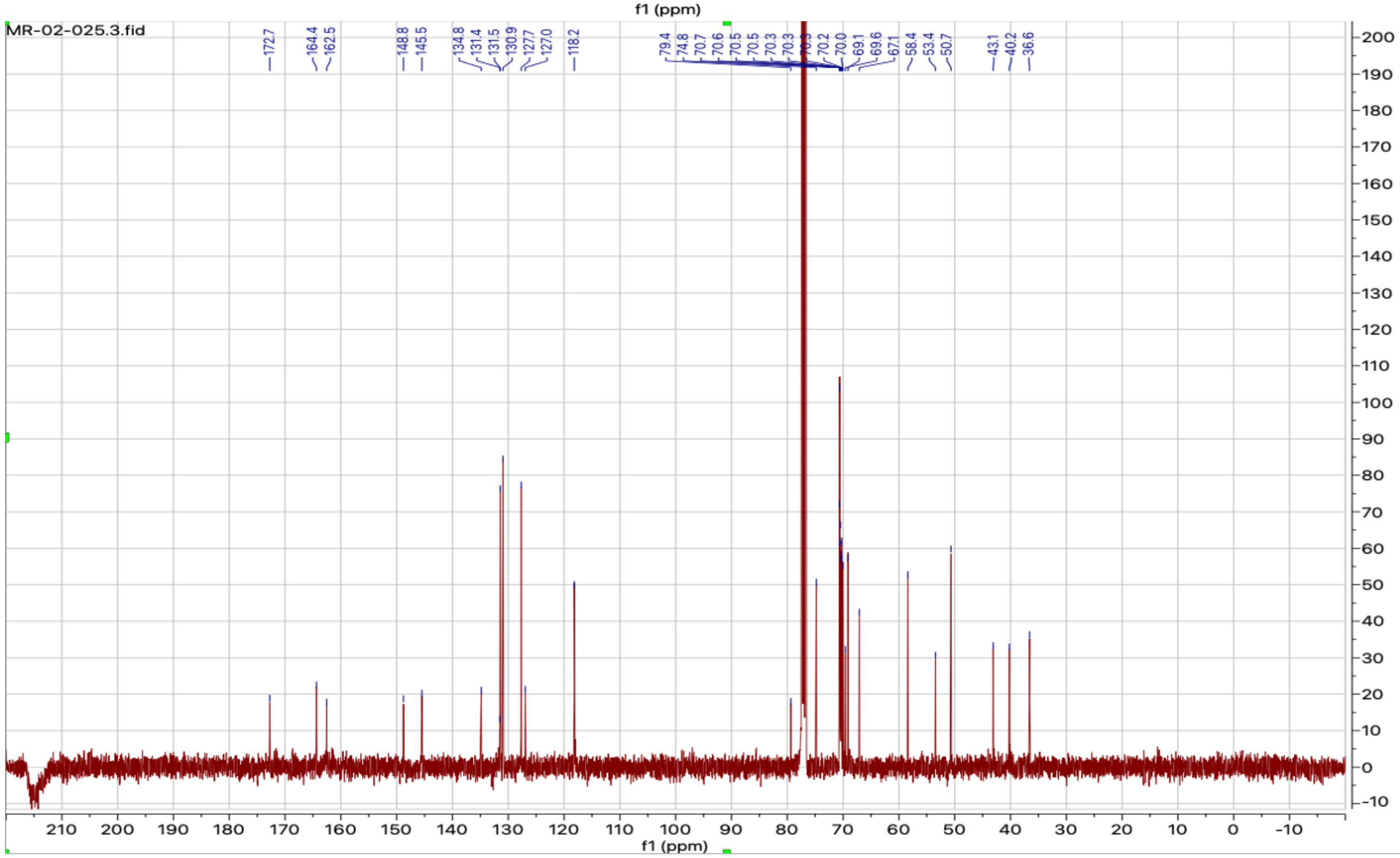
MR-02-025.3.fid
172.7
164.4
162.5
148.8
145.5
134.8
131.4
131.5
130.9
127.7
127.0
118.2
79.4
74.8
70.7
70.6
70.5
70.5
70.3
70.3
70.2
70.0
69.1
69.6
67.1
58.4
53.4
50.7
43.1
40.2
36.6
f1 (ppm)

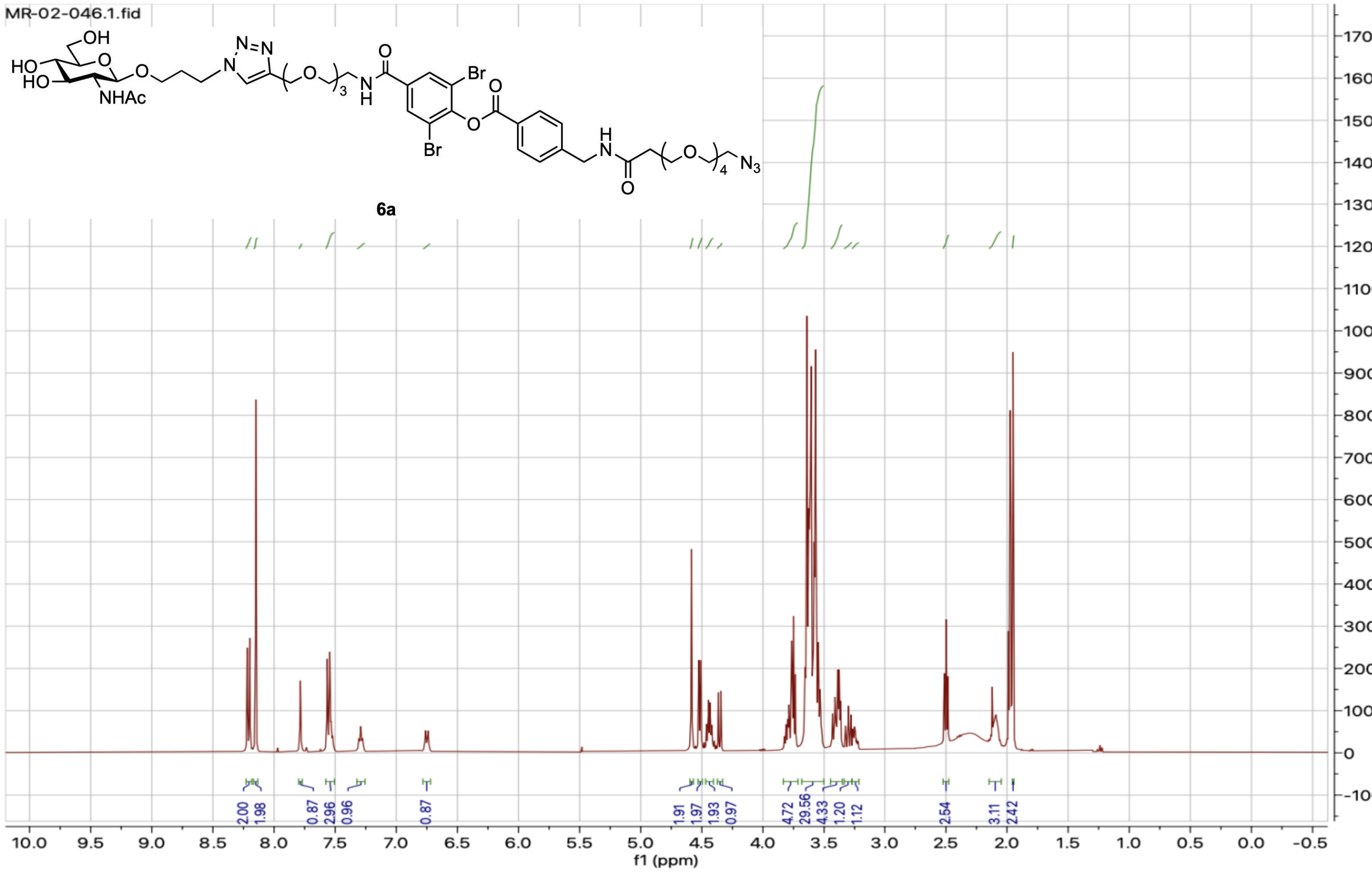
MR-02-046.1.fid
6a
2.00
1.98
0.87
2.96
0.96
0.87
1.91
1.97
1.93
0.97
4.72
29.56
4.33
1.20
1.12
2.54
3.11
2.42
f1 (ppm)

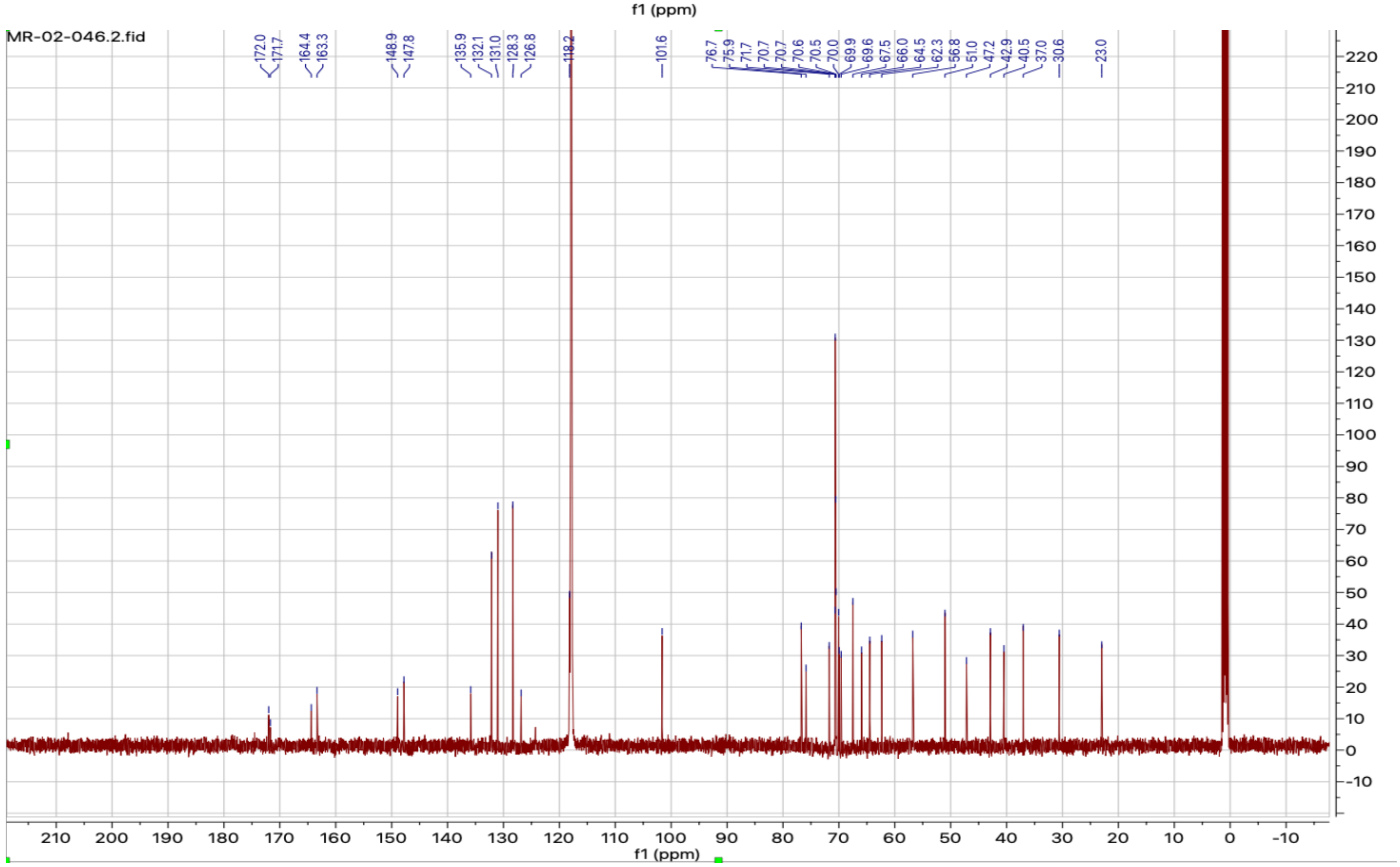
MR-02-046.2.fid
172.0
171.7
164.4
163.3
148.9
147.8
135.9
132.1
131.0
128.3
126.8
118.2
101.6
76.7
75.9
71.7
70.7
70.7
70.6
70.5
70.0
69.9
69.6
67.5
66.0
64.5
62.3
56.8
51.0
47.2
42.9
40.5
37.0
30.6
23.0
f1 (ppm)

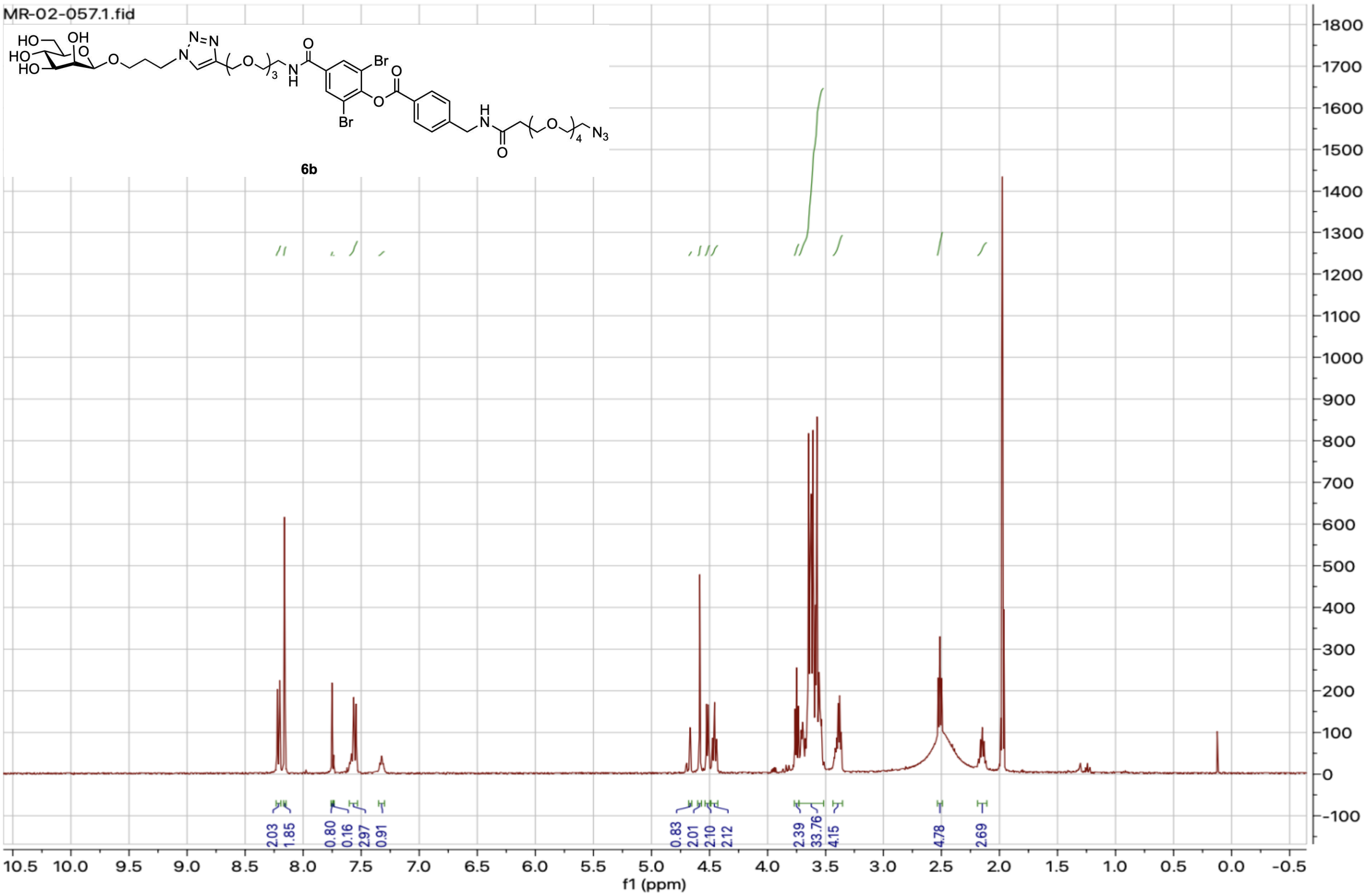
MR-02-057.1.fid
6b
f1 (ppm)

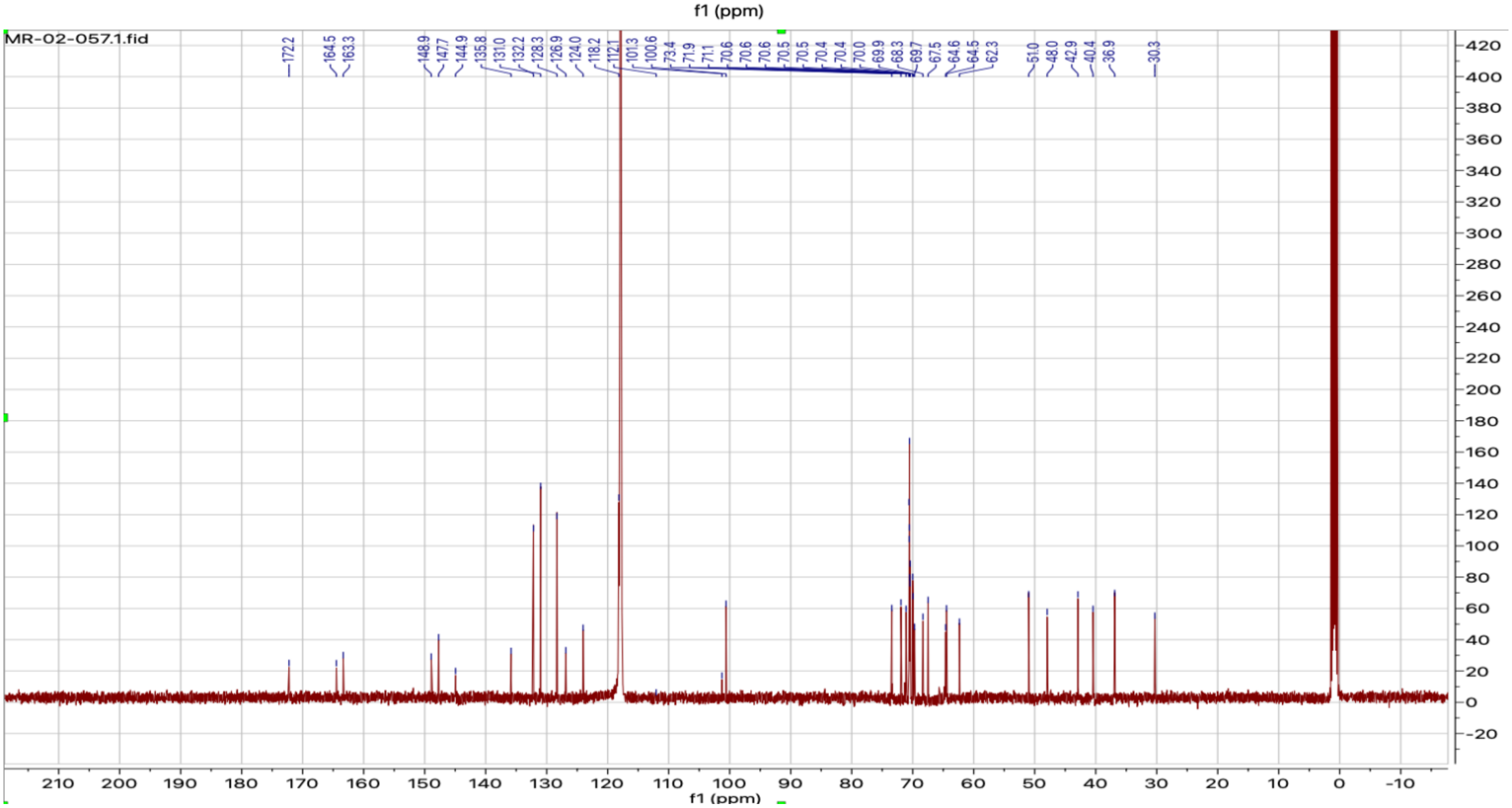
MR-02-057.1.fid
f1 (ppm)

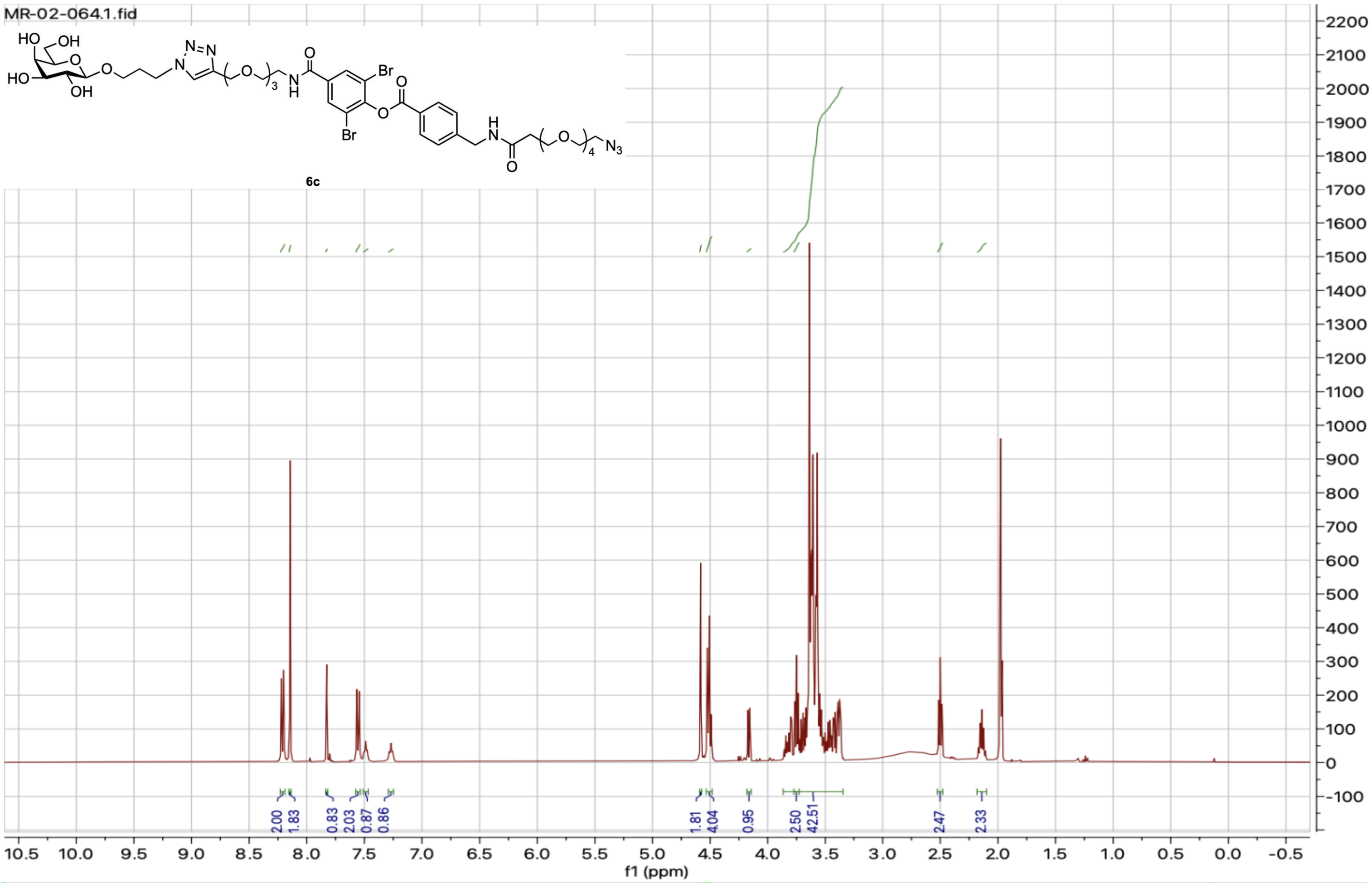
MR-02-064.1.fid
6c
f1 (ppm)

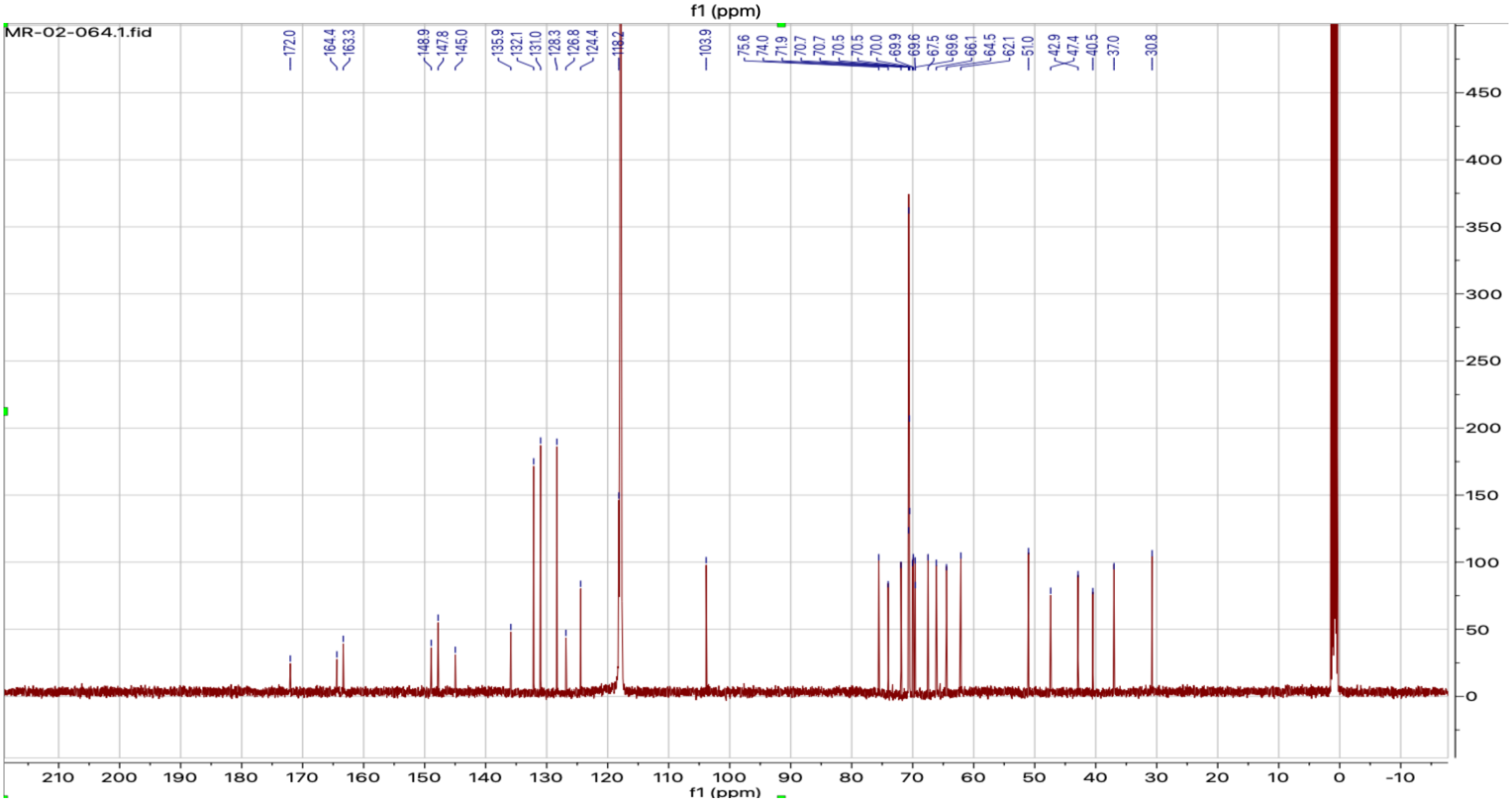
MR-02-064.1.fid
f1 (ppm)

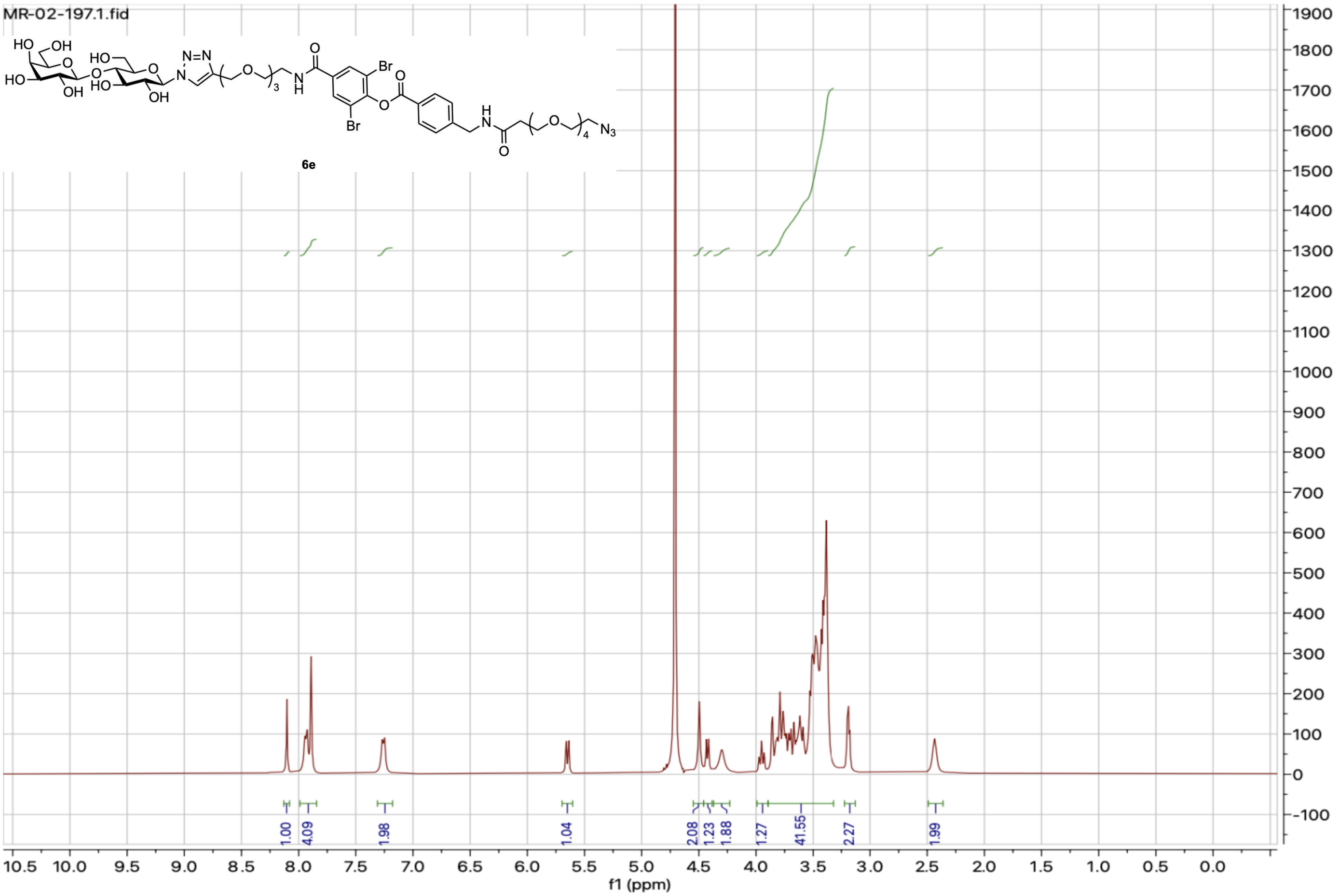
MR-02-197.1.fid
6e
1.00
4.09
1.98
1.04
2.08
1.23
1.88
1.27
41.55
2.27
1.99
f1 (ppm)

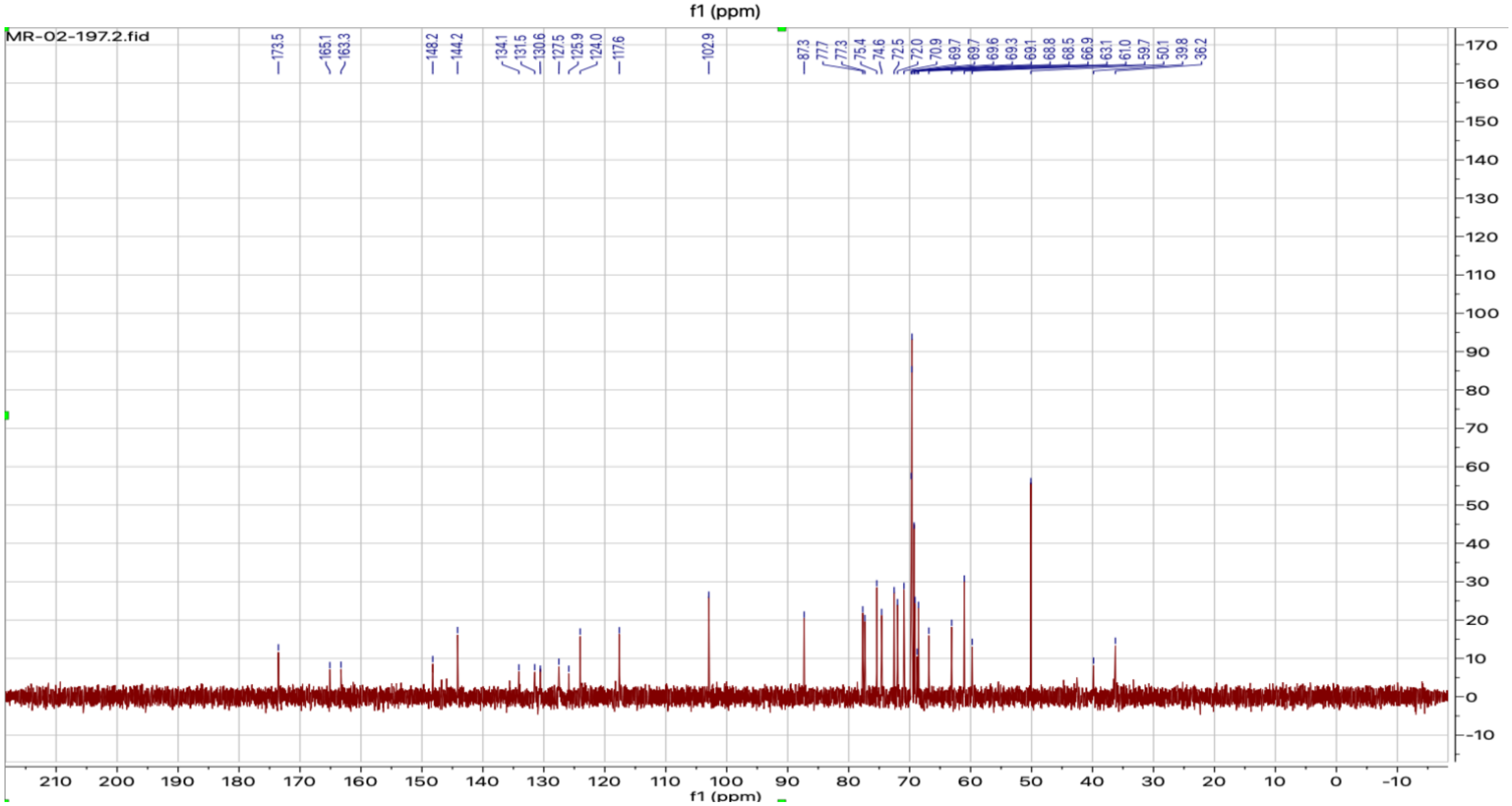
MR-02-197.2.fid
173.5
165.1
163.3
148.2
144.2
134.1
131.5
130.6
127.5
125.9
124.0
117.6
102.9
87.3
77.7
77.3
75.4
74.6
72.5
72.0
70.9
69.7
69.7
69.6
69.3
69.1
68.8
68.5
66.9
63.1
61.0
59.7
50.1
39.8
36.2
f1 (ppm)

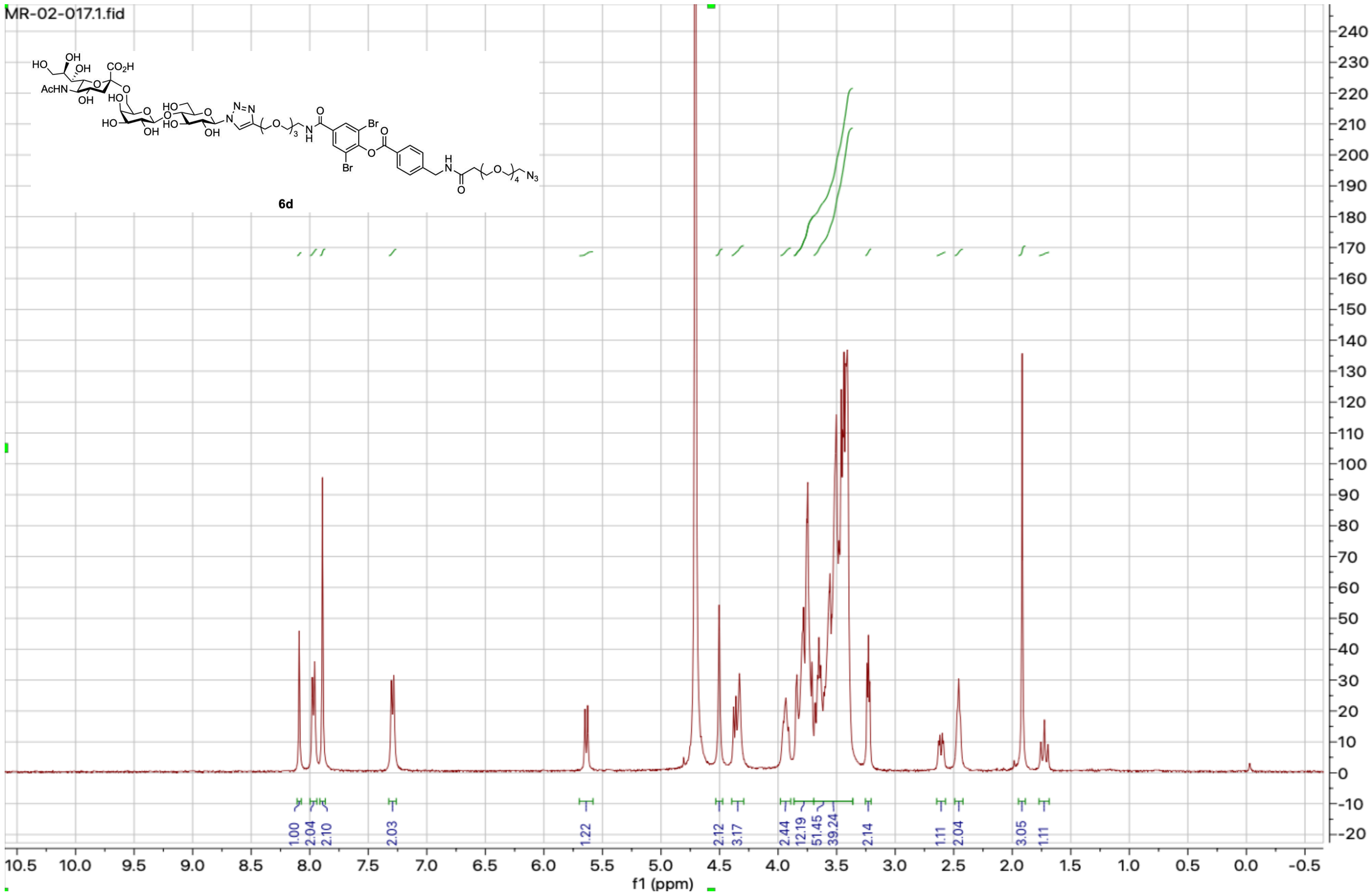
MR-02-017.1.fid
6d
f1 (ppm)

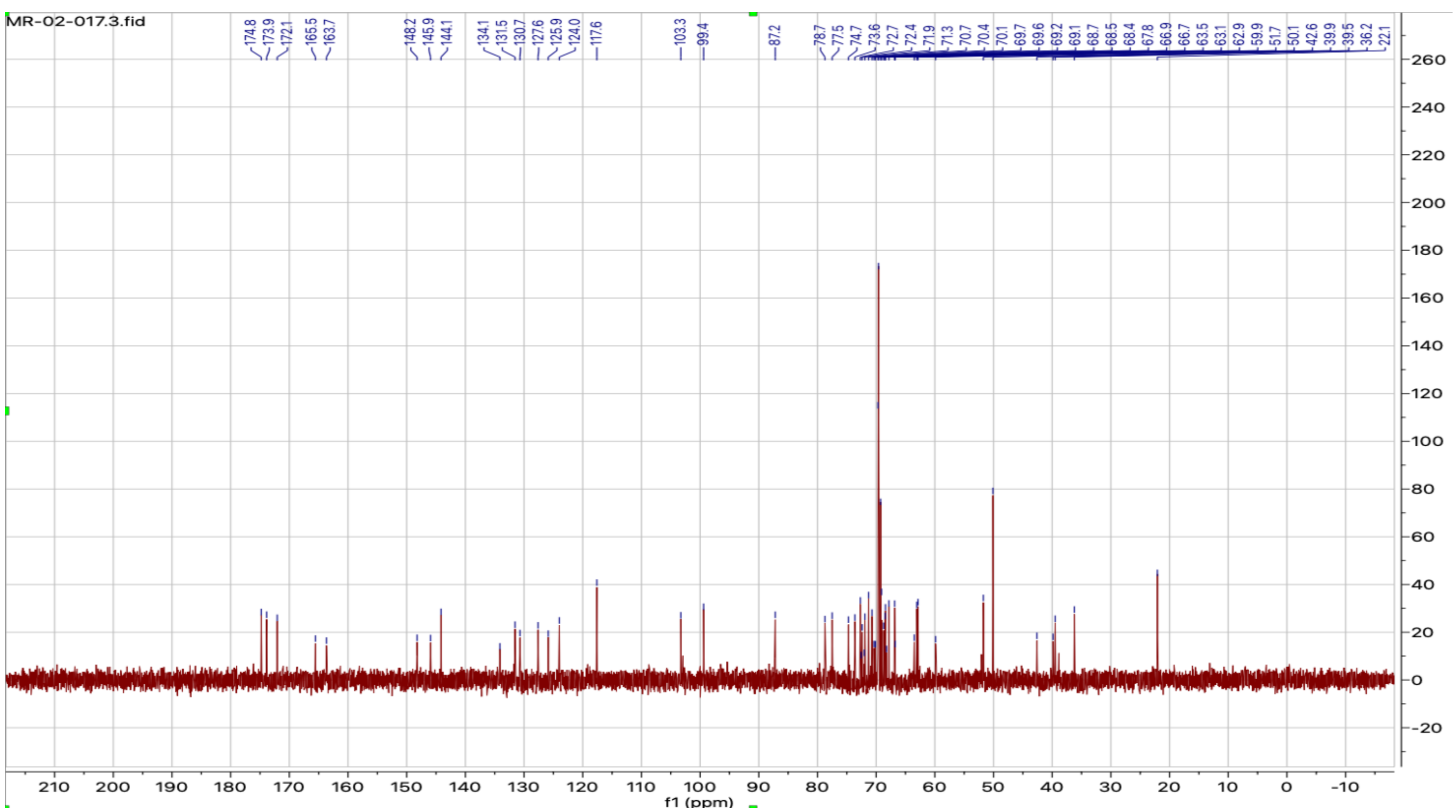
MR-02-017.3.fid
f1 (ppm)

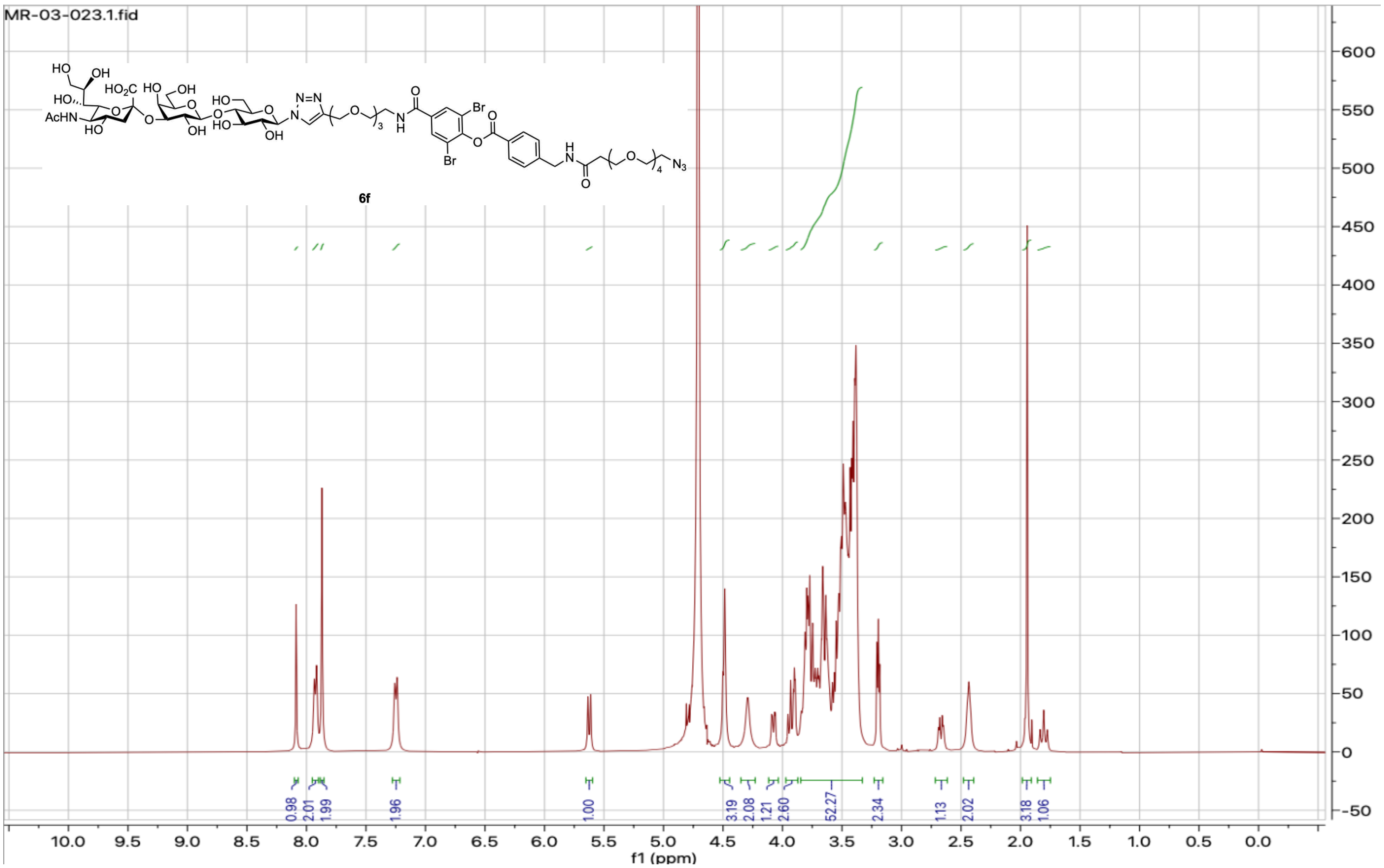
MR-03-023.1.fid
6f
f1 (ppm)
0.98 2.01 1.99 1.96 1.00 3.19 2.08 1.21 2.60 52.27 2.34 1.13 2.02 3.18 1.06

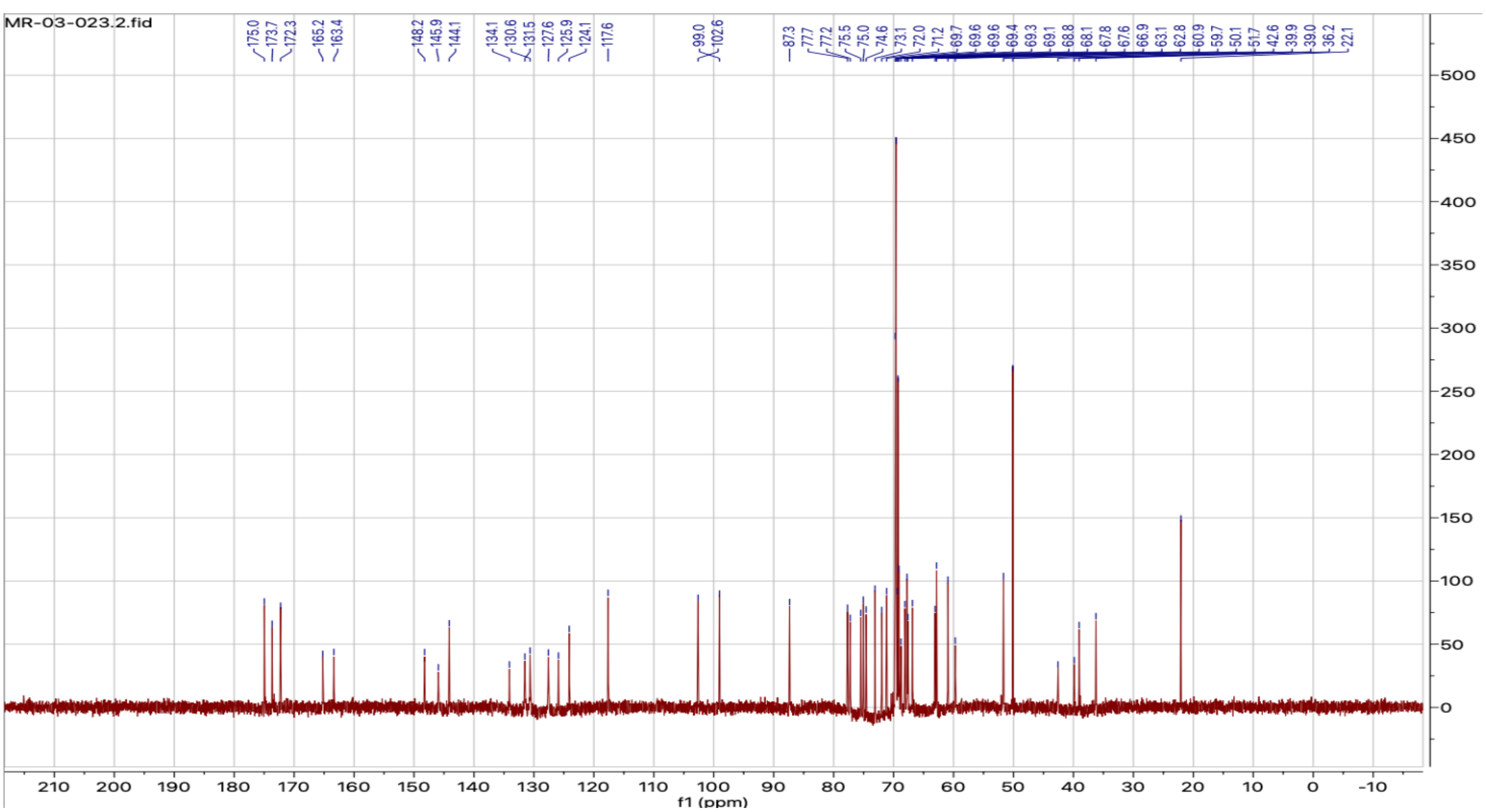
MR-03-023.2.fid
175.0 173.7 172.3 165.2 163.4 148.2 145.9 144.1 134.1 130.6 131.5 127.6 125.9 124.1 117.6 99.0 102.6 87.3 77.7 77.2 75.5 75.0 74.6 73.1 72.0 71.2 69.7 69.6 69.6 69.4 69.3 69.1 68.8 68.1 67.8 67.6 66.9 63.1 62.8 60.9 59.7 50.1 51.7 42.6 39.9 39.0 36.2 22.1
f1 (ppm)

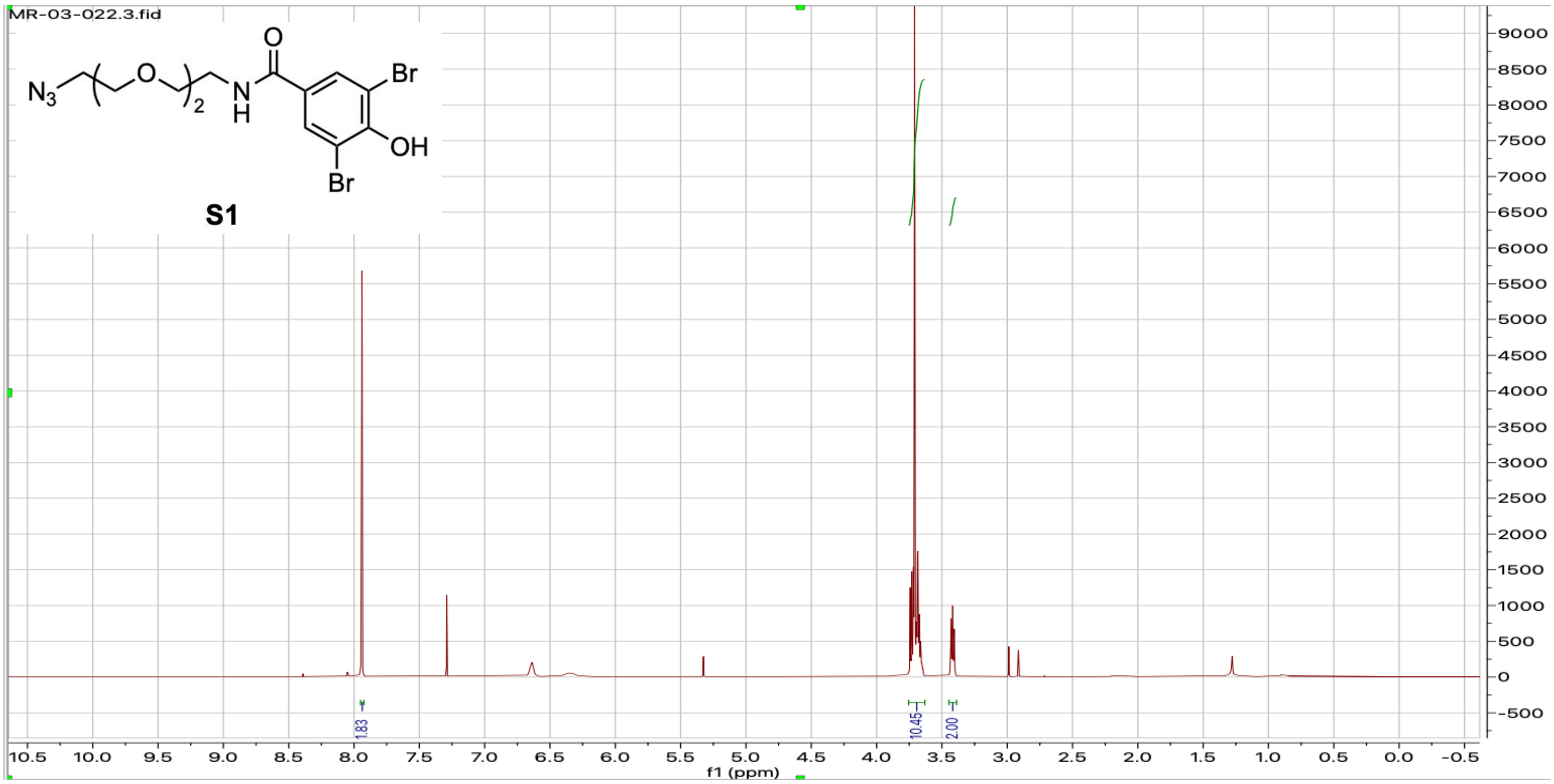
MR-03-022.3.fid
S1
1.83
10.45
2.00
f1 (ppm)

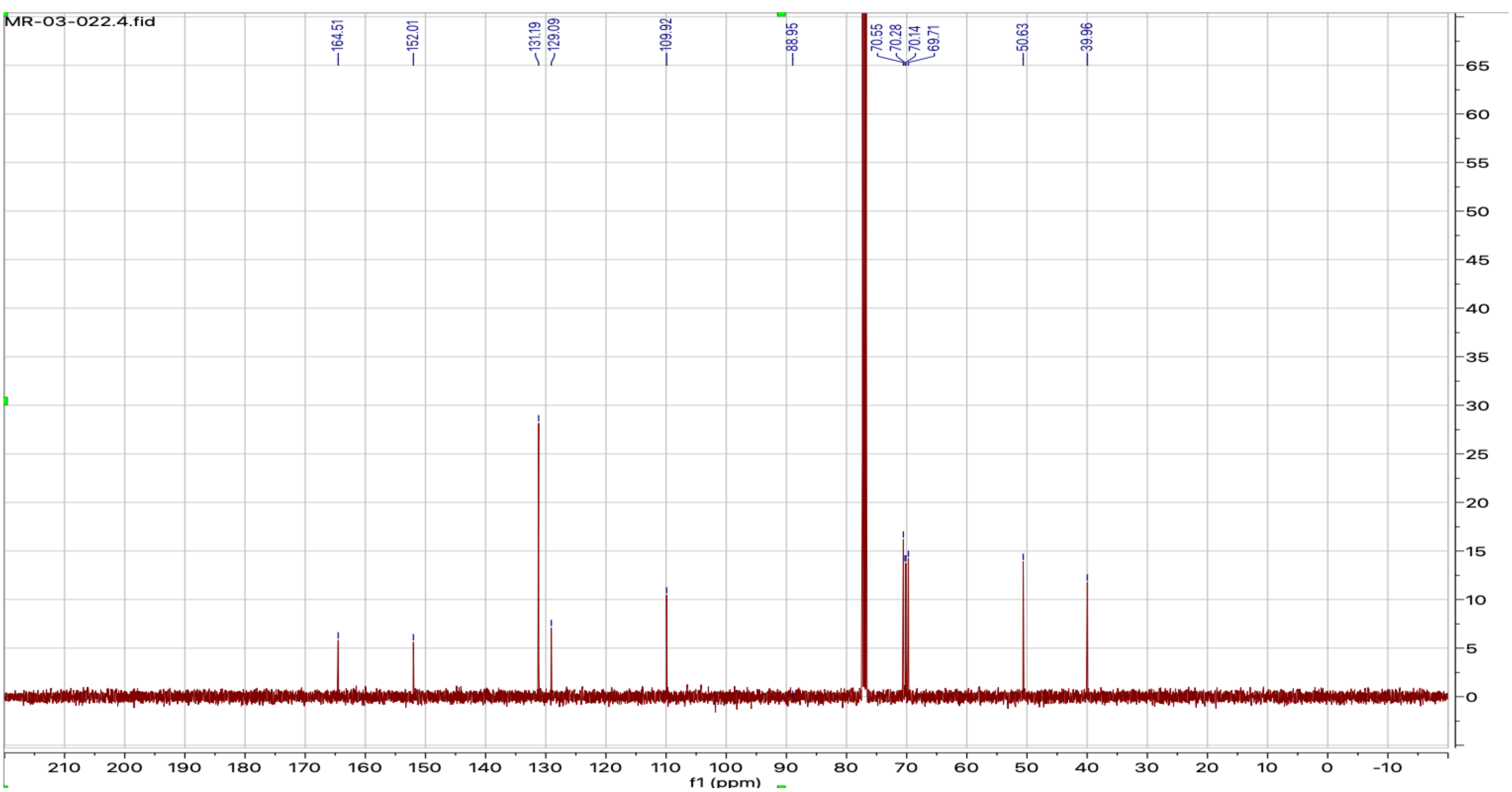
MR-03-022.4.fid
164.51
152.01
131.19
129.09
109.92
88.95
70.55
70.28
70.14
69.71
50.63
39.96
f1 (ppm)

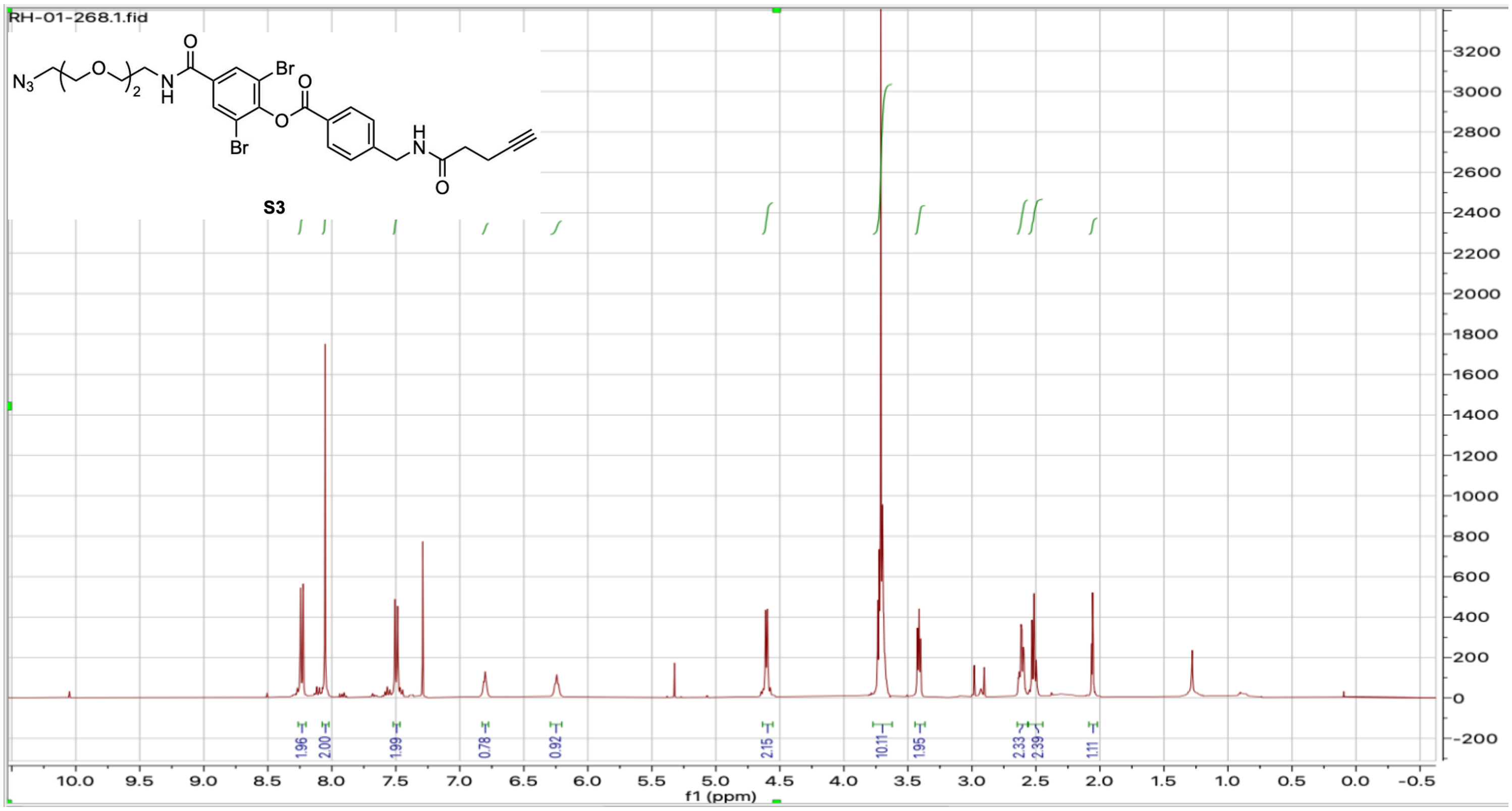
RH-01-268.1.fid
S3
1.96
2.00
1.99
0.78
0.92
2.15
10.11
1.95
2.33
2.39
1.11
f1 (ppm)

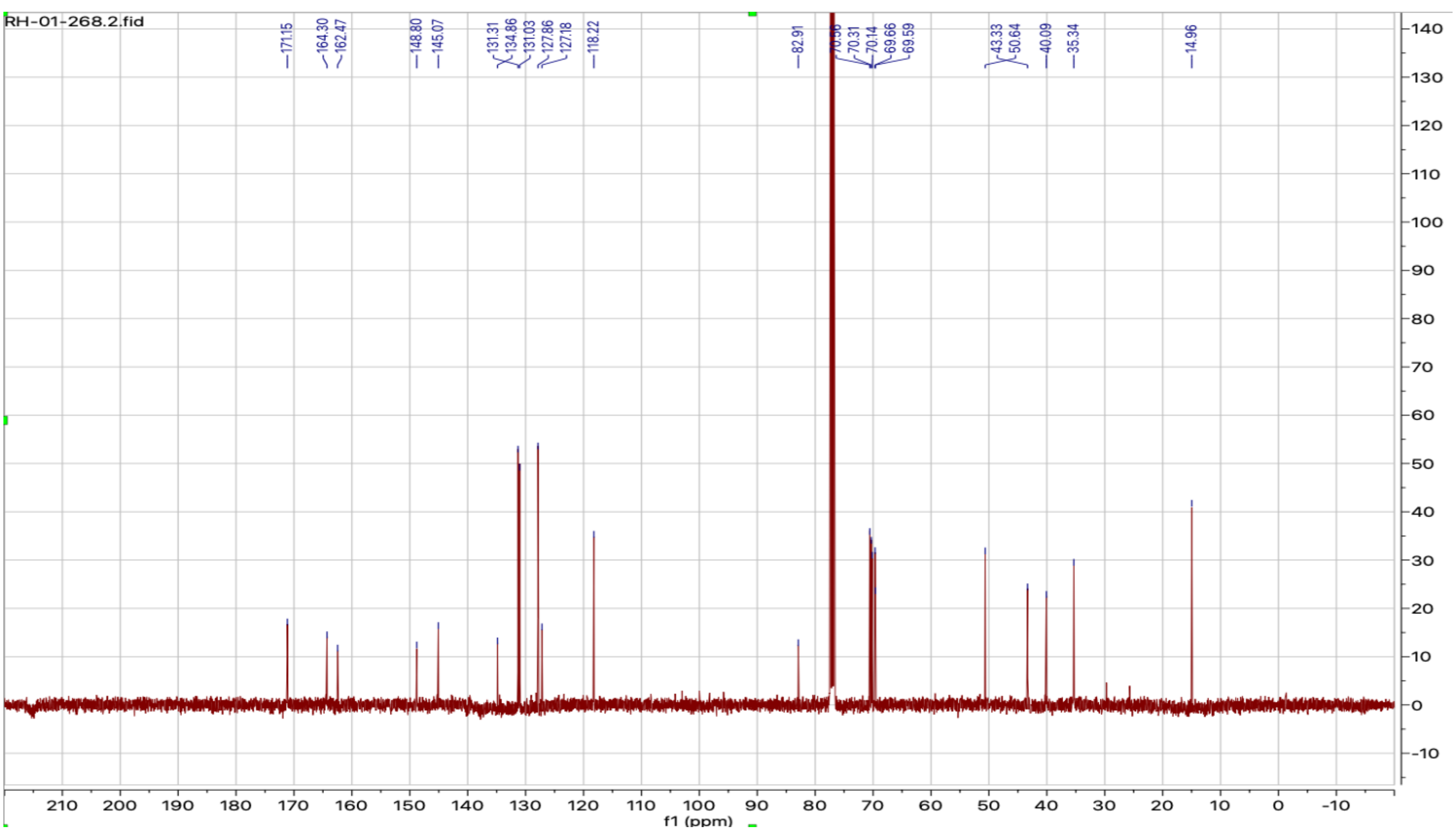
RH-01-268.2.fid
171.15
164.30
162.47
148.80
145.07
131.31
134.86
131.03
127.86
127.18
118.22
82.91
70.31
70.14
69.66
69.59
43.33
50.64
40.09
35.34
14.96
f1 (ppm)

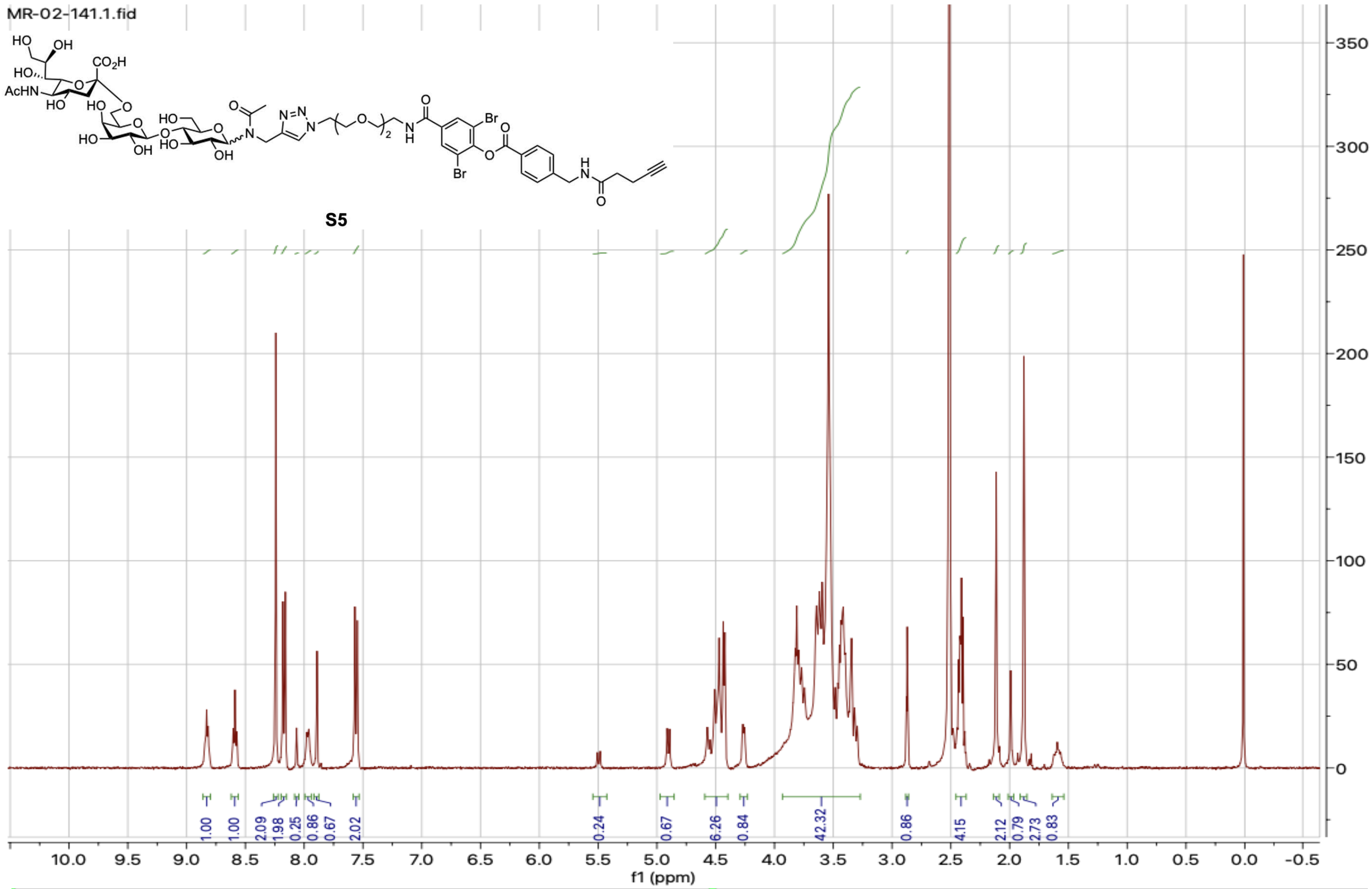
MR-02-141.1.fid
S5
1.00
1.00
2.09
1.98
0.25
0.86
0.67
2.02
0.24
0.67
6.26
0.84
42.32
0.86
4.15
2.12
0.79
2.73
0.83
f1 (ppm)

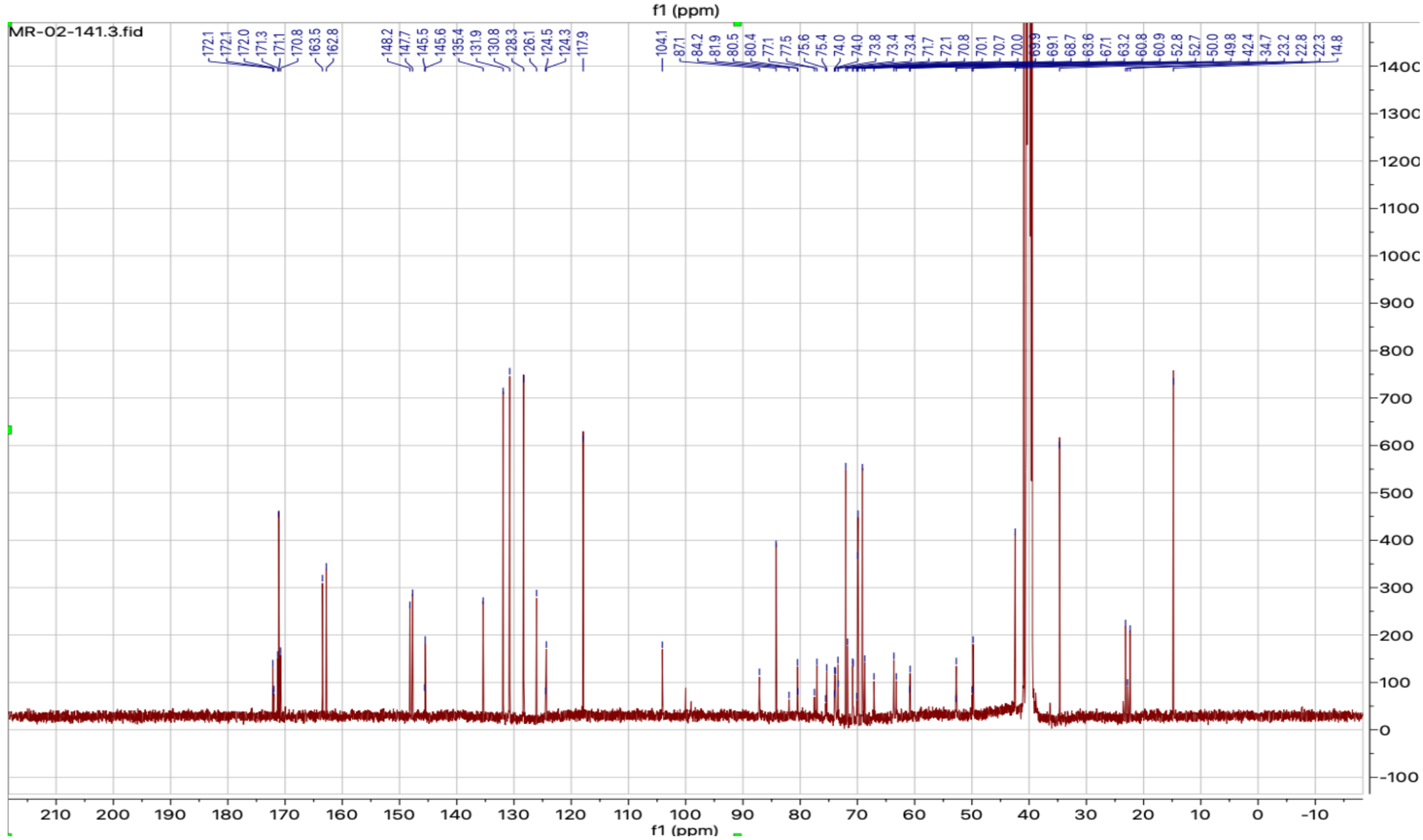
MR-02-141.3.fid
172.1
172.1
172.0
171.3
171.1
170.8
163.5
162.8
148.2
147.7
145.5
145.6
135.4
131.9
130.8
128.3
126.1
124.5
124.3
117.9
104.1
87.1
84.2
81.9
80.5
80.4
77.1
77.5
75.6
75.4
74.0
74.0
73.8
73.4
73.4
71.7
72.1
70.8
70.1
70.7
70.0
69.9
69.1
68.7
63.6
67.1
63.2
60.8
60.9
52.8
52.7
50.0
49.8
42.4
34.7
23.2
22.8
22.3
14.8
f1 (ppm)
